# Targeted sequencing enhances detection of pangolin trafficking hotspots and dynamics of both domestic and global trade markets

**DOI:** 10.1371/journal.pbio.3003762

**Published:** 2026-05-07

**Authors:** Sean P. Heighton, Jérôme Murienne, Mukesh Thakur, Alain Didier Missoup, Wirdateti Wirdateti, Chabi Sylvestre Djagoun, Sery Bi Gonedelé, Gabriel Ngua Ayecaba, Brice Roxan Momboua, Flobert Njiokou, Anne-Lise Chaber, Helen C. Nash, Barbora Černá Bolfíková, Sylvain Dufour, Guy T. Gembu, Ayodeji Olayemi, Jordi Salmona, Amaia Iribar, Yves Cuenot, Philippe Gaubert

**Affiliations:** 1 Centre de Recherche sur la Biodiversité et l’Environnement (CRBE), Université de Toulouse, CNRS, IRD, Toulouse INP, Toulouse, France; 2 Mission Pour l’Expertise Scientifique, MOY1698, UMR M2C, CNRS, Université de Caen Normandie, Caen, France; 3 Zoological Survey of India, New Alipore, Kolkata, West Bengal, India; 4 Zoology Unit, Laboratory of Biology and Physiology of Animal Organisms, Faculty of Science, University of Douala, Douala, Cameroon; 5 Research Center for Biosystematics and Evolution, National Research and Innovation Agency (BRIN), KST Soekarno, Cibinong, West Java, Indonesia; 6 Laboratoire d’Ecologie Appliquée, Faculté des Sciences Agronomiques, Université d’Abomey-Calavi, Cotonou, Benin; 7 Laboratoire de Biotechnologie, Agriculture et Valorisation des Ressources Biologiques, UFR Biosciences, Université Félix Houphouët Boigny d’Abidjan-Cocody, Abidjan, Côte d’Ivoire; 8 Centre Suisse de Recherches Scientifiques en Côte d’Ivoire, Abidjan, Côte d’Ivoire; 9 Amigos de la Naturaleza y del Desarrollo de Guinea Ecuatorial (ANDEGE), Barrio Ukomba, S/N, Bata, Equatorial Guinea; 10 Agence Nationale des Parcs Nationaux, Libreville, Gabon; 11 Département de Biologie, Faculté des Sciences, Université des Sciences et Techniques de Masuku, Franceville, Gabon; 12 Laboratoire de Parasitologie et Ecologie, Faculté des Sciences, Universite de Yaoundé I, Yaoundé, Cameroon; 13 School of Animal and Veterinary Sciences, University of Adelaide, Adelaide, Australia; 14 Department of Biological Sciences, National University of Singapore, Singapore, Singapore; 15 Institute of Zoology, Zoological Society of London, Regent’s Park, London, United Kingdom; 16 Faculty of Tropical AgriSciences, Czech University of Life Sciences, Prague, Czech Republic; 17 SYLVATROP Consulting, Conches sur gondoire, France; 18 Département d’Ecologie et de Biodiversité des Ressources Terrestres, Centre de Surveillance de la Biodiversité, Faculté des Sciences de l’Université de Kisangani, Kisangani, Democratic Republic of Congo; 19 Natural History Museum, Obafemi Awolowo University, Ile-Ife, Nigeria; 20 Centro Interdisciplinar de Investigação Marinha e Ambiental (CIIMAR), Universidade do Porto, Terminal de Cruzeiros do Porto de Leixões, Porto, Portugal; Sun Yat-Sen University, CHINA

## Abstract

Pangolins have become emblematic of the global wildlife trade crisis due to intense trafficking for consumption and traditional medicine. Coupled with habitat loss, the illicit trade in pangolins has severely threatened wild populations. Genetic identification of distinct pangolin populations is an imperative step toward guiding effective and informed conservation management. These populations can serve as a reference for assigning seized individuals to their geographic origins, and thus tracing trafficking networks. However, pangolin population genetics studies have been hindered by limited sampling of geo-referenced individuals, largely due to the species’ elusive nature. To address this, we developed a tailored gene-capture approach targeting 671 loci totaling 627 kb with high evolutionary and adaptive value across all eight pangolin species. We optimized the approach for low-quality DNA, including samples from museum collections and wildlife trade, such as bushmeat and scale seizures. We reassessed range-wide population delineations for the three most traded species, the white-bellied (*Phataginus tricuspis*), Sunda (*Manis javanica*), and Chinese (*M. pentadactyla*) pangolins, highlighting the need for biogeographically consistent lineage nomenclature and spatially aware analyses to support coherent conservation planning. The unprecedented geo-referenced DNA database for the three species yielded snapshot insights into pangolin trafficking hotspots and trade dynamics of both domestic markets and global trade seizures, the former providing novel insights into bushmeat trade. Domestic trade reflects local and occasional cross-border sourcing, averaging 454 km across the three species, while international trafficking seizures in mostly scales point to broader, regional procurement. However, common sourcing regions between the two trade market types indicate their interconnectivity, suggesting that local trade may contribute to international trade supply. Our study identified significant international trade hotspots for the white-bellied, Sunda, and Chinese pangolins, centered around southwestern Cameroon, southwestern Borneo Island, and Myanmar, respectively. Addressing geo-referenced sampling gaps and increasing local-to-global seizure data over time may offer deeper spatiotemporal insights into pangolin trade dynamics. Our study design may serve as a replicable model for enabling authorities and practitioners to implement intelligence-driven, geographically targeted interventions, by identifying the key regions most implicated in pangolin trafficking.

## Abstract 2

Pangolins are pursued globally for consumption and traditional medicine, however the key regions most implicated in trafficking remain unknown. By decoding DNA from museum specimens and seizures, this study reveals hidden pangolin trade routes, trafficking hotspots and distinct populations that can be used to guide targeted conservation management and disrupt the global illegal wildlife trade.

## Introduction

The illegal wildlife trade (IWT) contributes significantly to transnational crime, with over 140,000 seizures of 4,000 plant and animal species recorded across 162 countries between 2015 and 2021 [1]. Apart from population decline and local extirpation [2], the IWT has additional far-reaching consequences, such as disease epidemics [3] and the spread of exotic species [4]. These not only inversely impact wildlife conservation but also have major socio-economic consequences affecting people’s livelihoods and contributing to GDP losses [5]. Despite these drastic impacts, interventions to counter-wildlife trafficking still lack evidence-based approaches [1].

Population genomics has a pivotal role in the characterization of distinct conservation management units [6,7], providing practitioners with crucial insights for informed management policies—such as translocations, genetic rescue, captive breeding, and delineation of species strongholds—which can counter the deleterious impacts of the wildlife trade [8,9]. These insights additionally provide the means for employing a DNA-based tool known as geographic traceability or provenance assignment testing [10]. The method is based on the ability to assign a sample of unknown geographic provenance to its population or geographic region of origin and has been applied to ivory trafficking [11], fishery fraud [12], and timber trade [13].

A major limitation in geographic traceability and conservation genomics is the need for a sufficient number of geo-referenced samples spanning the species’ range [7,10]. Achieving this, especially from an elusive or endangered species, is challenging, resource-intensive, and time-consuming. Since museums contain specimens collected from across the globe, tapping into this resource can reduce these logistical and cost-related burdens when accurate specimen location data are available [14]. However, low yield and compromised DNA with high levels of contamination (exogenous DNA) in archival specimens, and indeed wildlife trade samples from bushmeat markets and trade seizures, render them less valuable for obtaining accurate insights at low cost [15,16]. Gene-capture, also referred to as hybridization-based targeted sequencing, can lighten this concern as small RNA probes are developed to enrich small fragments of degraded, endogenous DNA, thus increasing the relative DNA yield of the target species for Next-Generation Sequencing (NGS); [17,18]. The method has been implemented in evolutionary history and monitoring studies on high-conservation-concern mammalian species from suboptimal DNA samples (i.e., archival and eDNA samples: [19,20]).

Pangolins, an unfortunate poster child for the illicit wildlife trade, are a clade of highly divergent mammals consisting of eight, currently recognized species (but possibly more, see: [21–23]). Their trade can be characterized by either meat products for local, domestic consumption (e.g., bushmeat) or scales for traditional medicine that is frequently traded internationally [1]. The bushmeat trade is highly localized across Asian and African range states and represents an important source of nutrition for local communities adjacent to forests [24,25]. Beyond subsistence use, however, it also represents a broader domestic trade, often supplying bushmeat and body parts for local traditional medicine to major urban markets sourcing products from across the region [25,26]. The bushmeat trade is significant, with an estimated 0.4‒2.7 million individuals hunted and traded annually in local-to-regional markets in central African forests alone [26]. Despite its scale and socio-economic importance, the extent to which the domestic bushmeat trade is connected to the international trade in pangolin scales remains unclear [25]. The latter is almost exclusively linked to Asian traditional medicine end-consumer markets and has increased sharply since 2014, reaching over 100 tons in 2019 alone [1,27]. Pangolins rank among the taxa most frequently detected in international wildlife trafficking seizures worldwide, accounting for approximately 28% of recorded animal seizure incidents in recent years, according to UNODC’s Standardised Seizure Index [1]. Due to the recent decline of Asian pangolins, the African white-bellied pangolin (*Phataginus tricuspis*) has become the most heavily trafficked species in Asian markets, followed by the Asian Sunda (*Manis javanica*) and Chinese (*M. pentadactyla*) pangolins [27–29]. The two Asian species have historically been the most heavily trafficked [28].

The existential threat posed by both domestic and international trade, combined with uncertain impacts from habitat modification and climate change, has made pangolins a conservation priority. However, pangolins remain significantly understudied [30], complicating genetics-based efforts to develop effective conservation and anti-trafficking strategies. Initial attempts at range-wide population delineation [31,32] and subsequent population-based traceability of large seizures using mitochondrial DNA [33,34], or microsatellites [35–37] have focused on the white-bellied pangolin. Recently, genomic data have been used to gain higher geographic traceability resolution in the three most trafficked species [38–42]. However, most genomic studies thus far have been limited in delivering range-wide population delineation and geographic traceability, largely due to restricted geographic scope and insufficient reference samples. In addition, they have typically focused on population-level assignment rather than individual-level geographic assignment (however, see [38]), which predicts the spatial origin of each traded individual in continuous geographic space. This not only impedes accurate assignment of seized samples but also weakens the geographic and genomic foundation necessary for effective management planning.

Using both archival (museum) and recently collected samples across an unprecedented geographical range for the three most traded species (white-bellied, Sunda, and Chinese pangolins), we aim to (i) delineate and characterize populations for informed conservation management, as well as (ii) trace the local-to-global trafficking to uncover a snapshot in pangolin trade dynamics. To achieve this, we developed and applied a gene-capture approach capable of targeting all eight currently recognized pangolin species, contributing to the growing pangolin genetic toolkit and establishing a baseline for future management and trade intervention efforts.

## Results

We sequenced 711 gene-captured samples of the three targeted species at an average depth of 24×, capturing 671 loci representing approximately 607‒627 kbp of nuclear DNA for African and Asian species, respectively. Capture efficiency, the ratio between accurately mapped reads to the target region and total number of sequenced reads, was lower for the white-bellied pangolin when compared to the two Asian species (S1A Fig). Museum samples, which represent 17.16% of our data (*n* = 122), had similar capture efficiency compared to fresh samples (S1B Fig). However, museum samples exhibited lower target-region coverage at ≥10× depth (mean = 50.2%, SD = 36.2%) than fresh samples (mean = 68.5%, SD = 29.9%). An average of 65.4% (SD = 31.9%; median = 78.8%) of the target region was covered at ≥10× depth, with consistent declines in target region coverage across depth thresholds for the three targeted species (S1C Fig).

The 711 gene-captured samples were also supplemented with 92 whole-genome sequenced samples from previous studies, totaling 803 samples, which were delineated into the following site-types: reference samples of known localities (82 from field sites and 162 from small, restricted-range markets sourcing from surrounding forests) and samples of unknown localities for domestic and international trade tracing (351 from long-range markets representing domestic trade in urban hubs, 186 international trade seizures by authorities, and 22 samples with no given location data). All samples were mapped to the gene-capture reference of their respective continental clade, genotypes were called and filtered for each species, and the resulting datasets were used for population delineation and individual-level geographic assignment analyses (Refer to the S1 Text for more details on site-type definitions and seizures used, S1 Appendix for information on each sample, and S2 Fig for the distribution map of these samples).

### Population delineation

Nuclear DNA SNP datasets for each species were analyzed for population structure using Principal Component Analysis (PCA) and ADMIXTURE clustering. The influence of isolation-by-distance (IBD) on inferred structure was assessed using Mantel tests and triangle-plot analyses.

**Fig 1:**
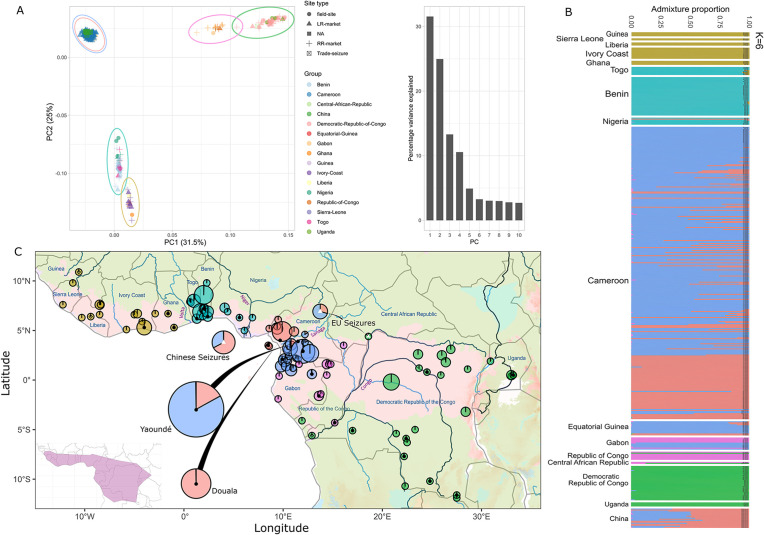
Population structure and stratification of the white-bellied pangolin (*Phataginus tricuspis*) using single-nucleotide polymorphisms (SNPs). **(A)** Principal Component Analysis (PCA; along with a histogram of variance explained per axis) for the top two axes of differentiation based on 8,520 SNPs and 427 individuals. Colored circles are reflective of clusters in ADMIXTURE. **(B)** Clustering bar graph for the most likely *K*-value (6) in which samples belonging to countries are clumped and each site type is labeled (8,312 SNPs, 456 individuals, 17.67% missing genotype data). Samples are ordered (from top down) from South-East to North-West in each country. **(C)** Geographic distribution of the six clusters: Upper Guinea (gold), Dahomey Gap (turquoise), Lower Guinea—north of the Sanaga river (red), Lower Guinea—south of the Sanaga river (blue), western Congolia (pink), and eastern Congolia (green). Pie-charts are size-scaled by the number of samples, whereby the admixture proportions of samples within 1.11 km were averaged. The two major urban bushmeat markets in Cameroon (Yaoundé and Douala) and seizures from China are placed in the sea due to the large sample size. EU Seizures in the map are nestled within their respective source countries in the PCA and ADMIXTURE plots. Pies with symbols are either long-range markets (black circle), trade seizures (white triangle), or unknown (unfilled diamond). Base layers include country and river data from Natural Earth (http://www.naturalearthdata.com; darker blue indicates larger rivers—key rivers are labeled in purple), elevation from GlobalSolarAtlas-v2 (https://globalsolaratlas.info, in deep red), ecological land units from U.S. Geological Survey (https://data.usgs.gov, each color represents an ecoregion), and the modeled species’ range from occurrence data previously collated (https://data.nhm.ac.uk/dataset/natalie-cooper; [43]). For a colorblind friendly version of the figure, see S7 Fig. The underlying numerical data are provided in S1 Data.

Axes PC1 and PC2 of the PCA for the white-bellied pangolin (*P. tricuspis*) separates genomic variance into three major geographic clusters, corresponding to the western (Guinea to the western bank of Niger River), central (eastern bank of Niger to the western bank of Congo River), and eastern (east of Congo River, with one exception from littoral Congo) parts of the species’ range (Fig 1A). Further sub-structuring within these major geographic clusters can be observed with the ADMIXTURE clustering output (most likely number of clusters *K* = 6; S3 Fig) and the PCA when zoomed in or when accounting for PC3 and PC4 (S4 and S5 Figs). The western cluster separates into Upper Guinea (from Guinea to Ghana) and Dahomey Gap (from Togo to Nigeria west of Niger River), while the eastern cluster separates into western Congolia (from central Gabon to the Central African Republic) and eastern Congolia (from the Republic of Congo to Uganda). The central cluster, corresponding to Lower Guinea, splits into two genomic groups separated by the Sanaga River in Cameroon, where some level of intermediate ancestry is noticeable. These individuals with intermediate ancestry from Lower Guinea and other clusters consistently showed low interclass heterozygosity in the triangle-plot analysis (S6B Fig), suggesting spatially continuous variation rather than recent admixture between discrete populations. Genetic and geographic distances were positively correlated, consistent with IBD (Mantel test, *r* = 0.73, *p* = 0.001; S6A Fig). However, the overall IBD pattern shows substantial genetic differentiation over short geographic distances and a plateau at larger distances, consistent with effects of geographic barriers and historical refugia.

Samples from Nigeria, Gabon, and the Republic of Congo are found in more than one cluster (Fig 1B and 1C). Both PCA and clustering results assign local trade seizures and long-range markets to their population clusters; examples include the Accra market (Ghana) to Upper Guinea cluster, Cotonou market (Benin) to Dahomey Gap cluster, and Douala and Yaoundé markets (Cameroon) to Lower Guinea cluster. With regards to international trafficking, seizures from both China and Europe identified the Lower Guinea cluster as a major source (see below for individual-level geographic tracing).

**Fig 2:**
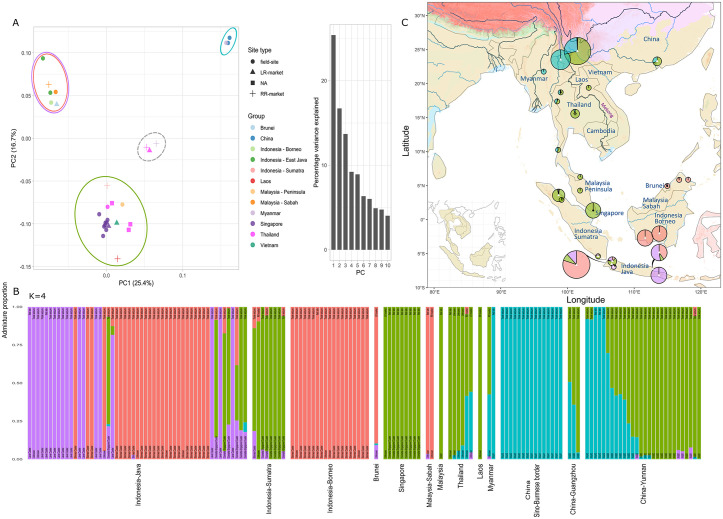
Population structure and stratification of the Sunda pangolin (*Manis javanica*) using single-nucleotide polymorphisms (SNPs). **(A)** Principal Component Analysis (PCA; along with a histogram of variance explained per axis) for the top two axes of differentiation based on 4,418 SNPs and 149 individuals (pictured are only the geographically referenced samples). Colored circles are reflective of clusters in ADMIXTURE, while the gray dashed circle is indicative of a cluster not aligned to ADMIXTURE. **(B)** Clustering bar graph for the most likely *K*-value (4) in which samples belonging to islands are clumped and each site type is labeled on the top part of each bar (4,162 SNPs, 148 individuals, 21.85% missing genotype data). Previously published clusters are labeled at the bottom of each bar [39,40]. Samples are ordered (from top down) from South-East to North-West in each island. **(C)** Geographic distribution of the four clusters: Borneo Island (red), Java Island (purple), Sumatra-Indochina (green), and Northeastern Montane region (turquoise). Pie-charts are size-scaled by the number of samples, whereby the admixture proportions of samples within 1.11 km were averaged. The two major seizures in eastern Java (Bogor—left and Surabaya—right) are placed in the sea on either side of the island due to the large sample size. Pies with symbols are either long-range markets (black circle), trade seizures (white triangle), unknown (unfilled diamond). See Fig 1 for the full legend for the base layers. For a colorblind friendly version of the figure, see S10 Fig. The underlying numerical data are provided in S1 Data.

When seizure samples are included, the PCA for the Sunda pangolin (*M. javanica*) does not indicate a clear population clustering pattern, with clines across the genomic space throughout PC1–PC4 (S8A–S8C Fig). Solely using geographically referenced samples, a separation into four clusters is noticeable, including the southern (Borneo and Java islands), central-eastern (Sumatra to Laos, including southern Thailand), central-western (northern Thailand to southern Myanmar), and north-western (northern Myanmar to western China) parts of the species’ range (Fig 2A). ADMIXTURE clustering results also suggest four clusters, largely overlapping PCA-based delimitations (best *K* = 4; S9 Fig). These include Borneo Island, Java Island, Sumatra-Indochina (Sumatra Island, Singapore, Peninsular Malaysia, southern Thailand, Vietnam, Laos, southern Myanmar), and the Northeastern Montane region (northern Thailand, Myanmar, Sino-Burmese border, Yunnan-China; Fig 2B and 2C). In line with the PCA-based projections, we observe admixture between the Borneo, Java, and Sumatra-Indochina clusters, mainly from seizures in Java and Sumatra. An admixture cline between the clusters Sumatra-Indochina and Northeastern Montane from south-east Thailand towards southern Myanmar is also noticeable in both the PCA (central-western individuals from Thailand and Myanmar) and clustering (with intermediary genotypes from Thailand and Myanmar), which is reflected in Yunnan seizures. The positive IBD trend (Mantel test, *r* = 0.68, *p* = 0.001) is consistent with overall clinal patterns (S6C Fig). However, the triangle-plot analysis shows elevated heterozygosity in intermediate individuals between Sumatra-Indochina and Northeastern Montane clusters, consistent with admixture rather than purely IBD-driven differentiation (S6D Fig). Other intermediate individuals between clusters Borneo, Java and Sumatra-Indochina show low heterozygosity and overlap, indicating shallow differentiation.

**Fig 3:**
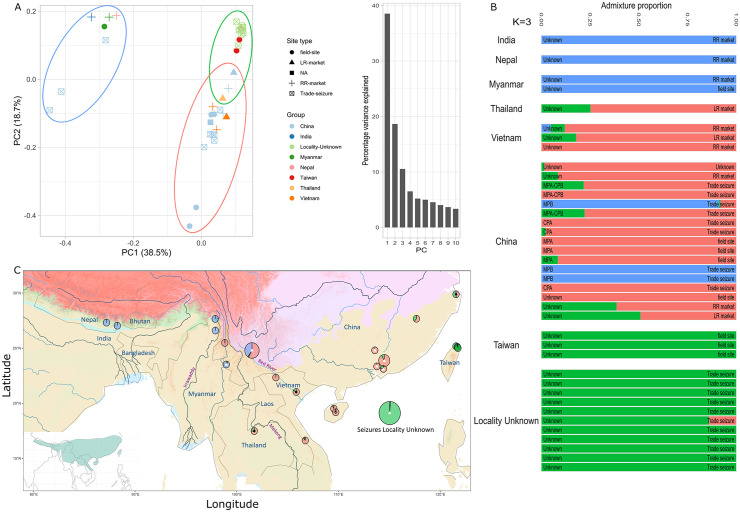
Population structure and stratification of the Chinese pangolin (*Manis pentadactyla*) using single-nucleotide polymorphisms (SNPs). **(A)** Principal Component Analysis (PCA; along with a histogram of variance explained per axis) for the top two axes of differentiation based on 840 SNPs and 39 individuals. Colored circles are reflective of clusters in ADMIXTURE. **(B)** Clustering bar graph for the most likely *K*-value (3) in which samples belonging to countries are clumped and each site type is labeled on the right of each bar (816 SNPs, 39 individuals, 24.06% missing genotype data). Previously published clusters are labeled on the left of each bar [39,41]. Samples are ordered (from top down) from South-East to North-West in each country. **(C)** Geographic distribution of these three clusters: Sino-Himalayan region (blue), Chinese-Indochinese region (red), and Taiwan (green). Pie-charts are size-scaled by the number of samples whereby the admixture proportions of samples within 1.11 km were averaged. Samples coming from an unknown locality were placed in the sea. Pies with symbols are either long-range markets (black circle), trade seizures (white triangle), unknown (unfilled diamond). See Fig 1 for the full legend for the base layers. For a colorblind friendly version of the figure, see S12 Fig. The underlying numerical data are provided in S1 Data.

Four PCA clusters are suggested for the Chinese pangolin (*M. pentadactyla*; Fig 3A), including western (India to Myanmar), central (western China), and southern-eastern (central Thailand to Taiwan on the southern range border) regions of the species’ range, plus an unknown locality (samples seized in Yunnan, China) showing genomic affinities with the western cluster. Clustering results suggest three clusters (best *K* = 3; S11 Fig) as the most likely structuring pattern. These include the Sino-Himalayan region (India, Nepal, Myanmar), the Chinese-Indochinese region (Thailand, Vietnam, China), and Taiwan (also including samples from long-range markets in eastern China; Fig 3B and 3C). There is a significant number of individuals with intermediate genotypes between the Taiwan and Chinese-Indochinese clusters (in the southernmost and easternmost range), which fits with PCA’s southern-eastern range grouping. The positive IBD trend is consistent with these clinal patterns but has a wider confidence interval, likely due to fewer samples (Mantel test, *r* = 0.50, *p* = 0.003; S6E Fig). The triangle-plot analysis indicates low interclass heterozygosity, suggestive of limited gene flow and strong barriers (S6F Fig). The PCA results mirror the Taiwanese and Sino‒Himalayan clustering, but separate the Chinese-Indochinese cluster between the Sino-Burmese border and the rest of the cluster, as well as the unknown cluster that contains two Chinese sample seizures. Using both PCA and clustering, seizures from China could be associated with the Sino‒Himalayan (seized in Yunnan and Sino-Burmese border) and Chinese-Indochinese clusters (seized in Guangdong and Yunnan provinces of China), whilst those collected/seized in an unknown location were associated with the Taiwanese cluster (Fig 3).

### Geographic origin tracing of traded samples

Individual-level geographic assignment based on nuclear and mitochondrial SNP data was performed using a deep-learning framework [44]. Species-specific models trained on geo-referenced individuals were used to predict origins for traded and unassigned samples. To assess the accuracy of origin-tracing predictions, we measured three complementary metrics. Prediction variability quantifies the spatial dispersion of bootstrap predictions to identify zones of confidence in assignment, one-third hold-out predictive error captures overall model robustness to data loss across the species’ range, and Leave-One-Out Cross-Validation (LOOCV) predictive error highlights spatially localized uncertainty driven by uneven or sparse regional sampling.

Prediction variability, the distances among all 100 bootstrapped predicted points and their centroid, was lower for the white-bellied pangolin (mean = 60.19 km; 95% CI = 8.72–154.78 km) compared to the Sunda (mean = 294.94 km; 95% CI = 37.55–948.61 km) and Chinese (mean = 411.03 km; 95% CI = 34.14–1366.97 km) pangolins (S13 Fig and S1 Table). One-third hold-out predictive error, the distance of true locality *versus* predicted locality when withholding around one-third of the reference samples across each species’ range from training data at once, was again lower for the white-bellied pangolin (mean = 132.35 km; 95% CI = 39.38–293.99 km) compared to the Sunda (mean = 415.17 km; 95% CI = 45.50–885.09 km) and Chinese (mean = 1032.03 km; 95% CI = 280.71–2126.19 km) pangolins (S13 and S14 Figs and S1 Table). LOOCV predictive error, the distance of true locality *versus* predicted locality when iteratively withholding each reference sample from the training data per run, saw similar trends for the white-bellied (mean = 180.1 km; 95% CI = 20.45–436.56 km), Sunda (mean = 575.52 km; 95% CI = 43.40–1527.27 km) and Chinese (mean = 1055.72 km; 95% CI = 82.16–2040.77 km) pangolins (S13 Fig and S1 Table).

**Fig 4:**
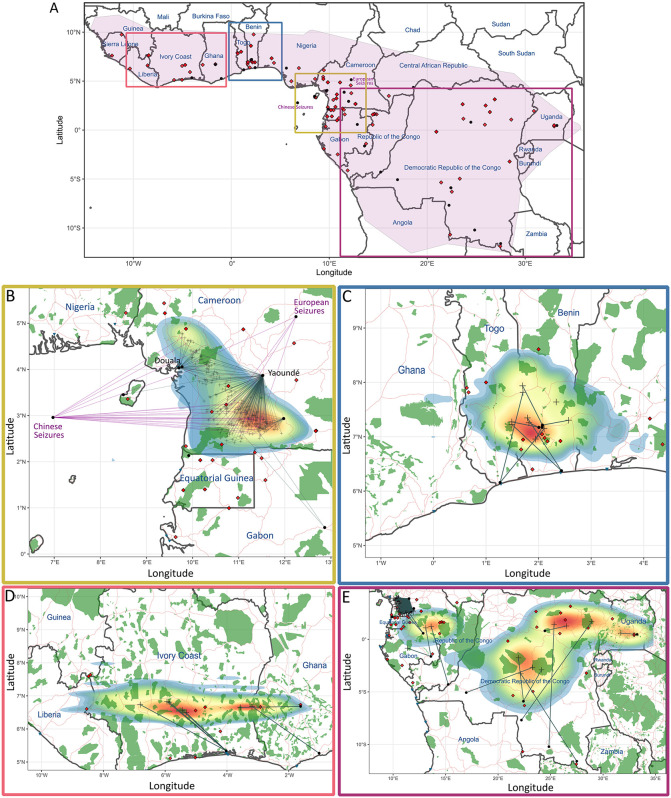
Origin tracing of traded white-bellied pangolins (*Phataginus tricuspis*). **(A)** Sampling map across the entire species’ range as well as detailed origin tracing maps for **(B)** Lower Guinea, **(C)** Dahomey Gap, **(D)** Upper Guinea, and **(E)** Congolia cluster regions (see S16 Fig for origin tracing across the entire species’ range map). Training locations are red diamonds and seized samples (black circles) are linked to their inferred origins (gray crosses) via gray (domestic) or purple (international) lines. The heatmap highlights likely sourcing density, with red indicating hotspots and heatmap bounds reflecting bootstrap confidence. Base layers include country, road (orange) and port (blue triangles) data from Natural Earth (http://www.naturalearthdata.com), protected areas (green) from the World Database on Protected Areas (https://www.protectedplanet.net), and the modeled species’ range from occurrence data previously collated (https://data.nhm.ac.uk/dataset/natalie-cooper; [43]). Further details on prediction accuracy are provided in S1 Table. The underlying numerical data are provided in S1 Data.

With exactly 34% of reference samples excluded in the white-bellied pangolin dataset (one-third hold-out), we were still able to identify the true localities of samples as low as within 19.69 km and as high as 424.09 km (S13 and S14 Figs and S1 Table). The most accurate estimate using the LOOCV approach for the white-bellied pangolin was within 8.67 km of the true sampling locality, whereas the least precise was within 1660.35 km (S13 Fig and S1 Table). These higher estimates were as a result of samples from the Democratic Republic of Congo (DRC), which had higher prediction variability (min = 1.32 km; mean = 161.61 km) compared to the overall for the species (min = 0.31 km; mean = 60.19 km; Figs 4E and S14). In terms of tracing from urban (long-range) bushmeat markets for the white-bellied pangolin, the domestic trade is generally localized, with sources of pangolins often found within the same country and area surrounding the markets themselves. Trade distances averaged 136 km (22–1,279 km, *n* = 275; S15 Fig and S2 Table) between the predicted sources and these markets (Fig 4). Despite localized distances, evidence of transnational trade between neighboring countries was found in Ghana (source: Côte d’Ivoire), Togo (source: Benin), Benin (source: Togo), south-western Nigeria (source: Benin), Gabon (source: Cameroon), Congo (source: Gabon), and Equatorial Guinea continental and Bioko Island (source: Cameroon; S16 Fig). Compared to all other clusters, pangolins traded in central Africa (Congolia cluster) were supposedly sourced from greater distances (but see above and the Discussion; Figs 4E and S15A). The main sources feeding the domestic trade in white-bellied pangolins could be traced to central-southern Côte d’Ivoire, south-western Ghana, southern Togo and Benin, south-western Cameroon, north-eastern Gabon, and central DRC. Dense sampling in Cameroon allows us to describe trade patterns more accurately for the two largest bushmeat markets in the country. The Douala market was almost exclusively sourcing from within its administrative division (Littoral Region), in and around the Ebo Wildlife Reserve (Fig 4B). In contrast, Yaoundé market was fed from a wider variety of sources, including a main source within the South Region near Ebolowa, the Centre Region near Eseka, and from the same area as Douala (Fig 4B). With regards to international trafficking, seizures from both China and Europe indicated sourcing within Cameroon, which overlapped the sourcing regions of Yaoundé and Douala (Fig 4B).

**Fig 5:**
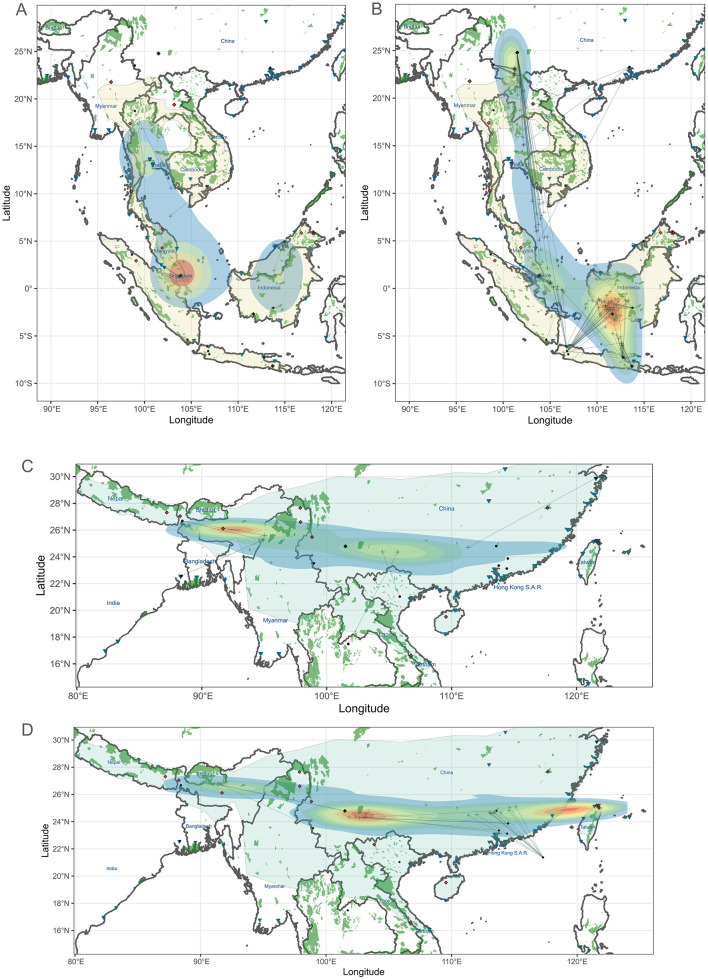
Origin tracing of trade in Sunda (*Manis javanica*) pangolins from (A) domestic and (B) international seizures, and Chinese (*Manis pentadactyla*) pangolins from (C) domestic and (D) international seizures (here, samples coming from an unknown locality were placed in the sea). See Fig 4 for the full legend, including the base layers. The underlying numerical data are provided in S1 Data.

For the Sunda and Chinese pangolins, our results suggest that source populations differ between the long-range market (domestic trade) and international seizures (Fig 5). For the Sunda pangolin, long-range markets are mostly sourced locally within areas like Singapore/Peninsular Malaysia and Borneo Island, though transnational sourcing may have occurred in Vietnamese and Thai markets (Fig 5A). The long-range, domestic market trade distances averaged 465 km (range 0.7–999 km, *n* = 4; S15C Fig and S2 Table). In contrast, international seizures revealed trafficking over greater distances (average 769 km; range 50–2,545 km, *n* = 121; S15C Fig and S2 Table) with major cities being supplied from distinct sourcing regions (Fig 5B). This includes seizures from Java (Jakarta and Surabaya harbors) being sourced from southwestern Borneo, and seizures from the Sino-Burmese border (China) being sourced from Yunnan province and possibly Myanmar and Laos. Conversely, seizures from Yunnan and Guangdong (China) regroup pangolins from a widespread range of source areas, including Yunnan, northern Vietnam, Thailand, Sumatra/Singapore, and Peninsular Malaysia.

Higher trade inference uncertainty is found for Chinese pangolins due to a more limited geo-referenced database (S13C and S14C Figs and S1 Table). However, long-range markets showed evidence of transnational trade in Chinese pangolins outside or at the edge of the species’ range, from Burma to northeastern India and from southwestern China to northern Vietnam and Thailand (Fig 5C). International seizures in China seem to have originated both from outside and within the country, with evidence of a network from northeastern India around Arunachal Pradesh and Assam (and possibly Bhutan) feeding Yunnan, while Yunnan was a potential source for Guangdong (Fig 5D).

## Discussion

To our knowledge, this is the first population genomics study jointly addressing the three most traded species of pangolins, whereas previous studies focused on the white-bellied pangolin [38], or partial ranges of Chinese and Sunda pangolins [39–42]. Additionally, the geo-referenced DNA database presented in this study is unprecedented in sampling density and geographic scale, leading to detailed population genomic insights and informative domestic and international trade tracing estimates at a range-wide scale for each species.

### Targeted sequencing optimizes genomic resolution in conservation studies

Our targeted sequencing through gene-capture approach enabled the effective use of degraded archival and seizure samples, thereby bolstering the geographic coverage of our geo-referenced DNA database. Gene-capture has proven particularly valuable in conservation genomics due to its ability to recover data from degraded samples with substantial levels of exogenous DNA [18,19]. We leveraged this feature to access valuable archival material, including 122 specimens (17.16% of the total 711 samples) from 15 museums, which remain underutilized in population genetic studies [14]. The higher capture efficiency observed in museum samples likely reflects the more stringent library preparation and capture protocols applied, whereas the lower target-region coverage reflects archival DNA degradation patterns [15]. By mapping whole-genome data to the bait reference, we integrated an additional 92 samples from previous studies [39,41] and shallow whole-genome sequencing. Together, these data sources expanded our geographic and population genomic scope to include hard-to-access regions and seized materials from key trade and consumer hotspots, a necessity for improved conservation of elusive species [17].

To minimize the impact of evolutionary distance on capture efficiency [45,46], we employed a dual-reference bait design (using one Asian and one African species) and conducted *in silico* testing across all eight pangolin species. This approach helped offset potential efficiency loss due to the deep phylogenetic divergence (*c*.40 million years) within Pholidota [22]. Our bait design thus permitted single-reaction multiplexing of all pangolin species whilst capturing sufficient endogenous DNA content. It therefore circumvents the need for prior species identification and for the development of multiple species-specific assays, thus reducing hands-on processing time and laboratory costs. This is especially useful for seizures containing multiple species, pangolin derivatives (scales, claws, bones), and pre-processed products (scale powder) [47]. By being a single, standardized pangolin assay, it also simplifies implementation across laboratories and facilitates broader uptake in conservation and forensic settings.

Compared to SNP arrays, gene-capture is more expensive per sample, but performs better on degraded DNA and recovers full sequence information that can be implemented in downstream analyses such as mutation load, coding *versus* non-coding variation, and evolutionary or functional patterns (for an in-depth comparison, see: [48]). These downstream analyses are more informative with WGS but remain cost-prohibitive when considering the high levels of exogenous DNA in museum and trade samples [18,19]. Our gene-capture kit for all eight pangolin species, also offers a key advantage over SNP arrays, which are typically designed for single species or closely related taxa and may perform poorly with highly divergent pangolin species. Importantly, our gene-capture data can be used to refine existing SNP panels [38] and to design future low-cost SNP arrays or targeted amplicon panels for highly informative and routine monitoring of seizures. However, the absence of technical replicates in this study precluded direct per-site estimates of genotyping error, which will be an important future consideration for interpreting gene-capture results and for designing SNP-panels and arrays.

### Genomics-informed population delineation in pangolins

Accurate population delineation is an essential precursor to informed population management and illegal trade tracing [6,7]. The delineation of six cryptic lineages of the white-bellied pangolin by Gaubert, Njiokou [31] provided the first necessary geo-referenced DNA database to which pangolins from seizures could be globally traced [33,34]. Our analysis reveals fine-scale regional structuring across each species’ range, with broad concordance to earlier delimitation patterns.

Overall, we provide a refined understanding of the white-bellied pangolin phylogeography, revealing five distinct regional clusters that closely align with major biogeographic regions of tropical Africa [49]: Upper Guinea (Guinea to Ghana), Dahomey Gap (Togo to southwestern Nigeria), Lower Guinea (southeastern Nigeria to northern Gabon), western Congolia (central Gabon to the Central African Republic), and eastern Congolia (the Republic of Congo to Uganda). The two clusters identified in Cameroon that show a cline of genetic variation were not supported by previous studies using mtDNA [31,50], but are supported by a genomic study [38]. However, our spatially aware analyses suggest that these subclusters reflect fine-scale spatial structure driven by IBD that is potentially amplified by an overrepresentation of samples from Cameroon rather than discrete evolutionary units [51,52]. We therefore suggest that these remain a single cluster (Lower Guinea), rather than two separate clusters as previously described [38].

Previous mtDNA analyses have split the Congolia cluster into western and eastern parts along the Congo River [31,50], which was described as a single cluster using genomic data from limited sample representation across its region [38]. With a more robust sample representation of Congolia our nuclear gene-capture dataset aligns with the mtDNA split along the Congo River. We also note a mito-nuclear discordance in the species, with original mtDNA clusters in Ghana and Gabon [31] not recovered in the nuclear gene-capture DNA, likely due to historical introgression with neighboring clusters (see [35,36]).

Our study also offers a more comprehensive, range-wide view of Sunda pangolin phylogeography, identifying four genetic clusters that align closely with major biogeographic barriers: Borneo Island, Java Island, Sumatra-Indochina (Sumatra Island, Singapore, Peninsular Malaysia, southern Thailand, Vietnam, Laos, southern Myanmar), and the Northeastern Montane region (northern Thailand, Myanmar, Sino-Burmese border, Yunnan-China). The pronounced IBD pattern indicates that much of the observed genomic variation is spatially continuous across the species’ range. However, admixture, rather than purely IBD-related differentiation, was observed among individuals with intermediate ancestry between the Sumatra-Indochina and Northeastern Montane populations. While these patterns can also arise under IBD during historical spatial expansion, their concordance with PCA and ADMIXTURE clines suggests that gene flow likely contributes to the observed patterns in this region. These gene flow signals may reflect secondary contact or long-term connectivity along montane corridors, in line with phylogeographic patterns in other forest-dependent mammals from this region [53,54]. These individuals were seized in Yunnan and Guangzhou and have been identified in previous studies (see MJB3 in: [39]). The Sumatra-Indochina and Northeastern Montane clusters align with the broadscale clusters identified as MJB and MJA, respectively [39]. However, the subclustering proposed by Hu, Hao [39] (MJA1–2 and MJB1–3) was not clearly recovered in our study, nor was it recovered in a recent genomic study [55]. The Sumatra-Indochina cluster combines the MJB1 (likely Peninsular Malaysia and southern Thailand), MJB2 (likely Vietnam and Laos), and MJB3 (likely northern Thailand and southern Myanmar) clusters [39]. It also closely aligns with the MJ3 cluster recently delineated by Li, Li [55]. The Northeastern Montane cluster [56] combines the two MJA subclusters (MJA1 and MJA2) and is in agreement with the western mtDNA clade of Banterng, Ewart [57]. Our split between the Northeastern Montane and Sumatra-Indochina clusters confirms the hypothesis of an east–west biogeographic division in Thailand for the Sunda pangolin [57], however, recent evidence may indicate this to be a single population [55]. While the Borneo and Java clusters align directly with those of Nash, Wirdateti [40], our extended sampling expands the Sumatra-Singapore cluster further north to southern Myanmar (here named the Sumatra-Indochina cluster). In contrast to a previous mtDNA study [32], we did not observe a split between northeastern and western Borneo (Sabah *versus* Sarawak/Kalimantan and also including peninsular Malaysia). Rather, Borneo, Java, and Sumatra-Indochinese peninsula constituted distinct but shallow populations, indicating mito-nuclear discordance. The mtDNA pattern may reflect refugial history across the Sunda Shelf during glacial periods [58], whilst the nDNA signature suggests recent isolation patterns, or that historical introgression acted differently on these markers—a common pattern in the region [59,60]. A recent genomic study has also suggested this split as MJ1 (northeastern Borneo) and MJ2 (western Borneo) [55]. However, this inference was based on a limited number of samples of known locality (two per group) and did not include samples from Java or southern Sumatra, which our study does. We therefore recommend that these three clusters (Borneo Island, Java Island, Sumatra-Indochina) be interpreted as partially differentiated lineages shaped by historical connectivity rather than as fully discrete, isolated populations, and that additional sampling within Borneo will be important to further resolve patterns of nuclear population structure in this region.

Taken together, our results suggest that while the Sunda pangolin can be partitioned into four geographically coherent genetic clusters, its population structure is best understood as a continuum with regional differentiation shaped by a mosaic of demographic processes, including IBD, geographic barriers, and localized admixture. These patterns are consistent with recent historical introgression across the Sundaland shelf during repeated Pleistocene land connections [42,55], but could also be attributable to sampling biases across different clusters [52,53] and to the potential influence of human-assisted migrations through trade [42]. It also reflects the complex evolutionary history of Southeast Asian pangolin species, which may have influenced the Sunda pangolin’s population structure [61]. To provide more informed conservation units, we underscore the need to account for historical connectivity and sub-regional demographic processes as well as for additional geo-referenced sampling in the north-eastern parts of the range (Cambodia, Vietnam, Laos) and key transitional zones like western Java to southern Sumatra, Sumatra to Peninsular Malaysia, and eastern Thailand to Cambodia.

With more samples of known origin for the Chinese pangolin than previous studies, our work provides an overview of the species’ phylogeography across its entire range, suggesting three clusters that align with major biogeographic regions: Sino-Himalayan region (India, Nepal, Myanmar), Chinese-Indochinese region (Thailand, Vietnam, China), and Taiwan (Taiwan, also including samples from long-range markets in eastern China). The IBD pattern indicates that observed genomic variation is partly spatially continuous across the species’ range, but with major geographic and historical barriers. Our clusters confirm and expand on the previously identified separation of the western [Sino-Himalayan cluster [MPB: [39]/[CPC: [41]] and central [Chinese-Indochinese cluster [MPA: 39]/[CPA/CPB: 41]] regions of the species’ range. We confirm Hu, Hao [39] MPA cluster of seized samples suggested to have come from southern China and Thailand, but extend it to include Vietnam and the north-western and eastern provinces of China (Yunnan, Fujian, Zhejiang). Because this lineage aligns with the Indochina biogeographic region and the Chinese transition zone, we refer to it as the Chinese-Indochinese cluster [62,63]. Previous studies proposed that the MPB [39] and CPC [41] seizure clusters likely originated from Myanmar, but lacked reference samples from the region to confirm these hypotheses. By incorporating samples from Nepal and India, our study provides supporting evidence for this delineation. Our nuclear DNA data confirm the existence of a Sino-Himalayan cluster [62,64], which was recently proposed as a distinct phylogenetic species, *Manis indoburmanica*, based on mitochondrial data [23]. This lineage has further been proposed for re-erection as a distinct species, *Manis aurita* (Hodgson, 1836 stat. nov.), based on morphological and genome-wide SNP analyses [65]. We found a substantial amount of variance (38.5%) separating this group on the first component of the PCA, which supports the hypothesis of an incipient or distinct lineage.

Like Hu, Hao [39] and Wei, Fan [42], we did not identify a separate cluster in eastern Guangdong [41], and contradict the author’s grouping of Taiwan with western Guangdong and Yunnan (CPB), instead of the more proximal eastern Guangdong (CPA). The authors suggested that the two clusters (CPA and CPB)—that we identify as a single cluster—diverged very recently (c. 2–4 kya), and those that we were able to separate (CPA/CPB *versus* CPC) diverged earlier, around 25–40 kya [41]. The MPC cluster of Wei, Fan [42] was allocated to Vietnam and Laos without any geo-referenced samples of this cluster in the region, which we contradict. In addition, we identified potential substructure within the major clusters based on PCA and detected a distinct cluster of unknown origin from seizures in Yunnan, which was not suggested in previous studies [39,41]. We hypothesize that it originates from southern Myanmar, specifically within the Northeastern Montane subregion of the Sino-Himalayan cluster.

Across all three species, we call for more detailed information on sampling locations and types (i.e., seizure or field sample), as well as a common population cluster/lineage naming system that relies on biogeographic regions in future studies. This will avoid confusion in population distinction, which could hinder conservation management. This is particularly pertinent for the Chinese pangolin, whereby the Sino-Himalayan cluster (this study) is named the MPB [39], CPC [41], MPC [42], and MpenC [66] clusters from previous studies, despite these studies having a subset of their data stemming from Hu, Hao [39]. Despite using a reduced marker set, our gene-capture approach had sufficient resolution to distinguish major population clusters previously identified in genome-wide studies [39,41,42]. However, some finer-scale subclusters, such as MJB1–3 [39] and CPA–B [41], were only partially resolved at higher *K* values or remained indistinct. Across all three species, IBD and triangle-plot patterns indicate that some genetic clusters may reflect segments along continuous spatial gradients or genuine admixture, rather than discrete populations, particularly in regions with uneven sampling [67]. Additional sampling of georeferenced populations is needed to fill these transitional zones and key critical gaps for the white-bellied (Republic of Congo and Angola), Sunda (Cambodia, Laos, Vietnam, and Sumatra), and Chinese (north and eastern China, and southern range) pangolins. This is especially important given that our study identified several population clusters with unresolved geographic origins—such as the unknown Chinese seizure cluster probably stemming from southern Myanmar—which may prove critical for informing future conservation strategies. While our study provides an accurate reassessment of population delineation within pangolins, additional and independent lines of evidence—such as morphological data, ecology and population demography—are still needed to achieve the ultimate goal of identifying robust conservation units [6].

### Accuracy of geographic assignment predictions

The accuracy of origin tracing in our study—and more broadly—depends on the number and geographic coverage of reference samples used to train the model, with better performances achieved when populations are strongly structured in space, and markers are informative of this structure [44]. When implementing the same LOOCV approach on our white-bellied pangolin geo-referenced samples, we attained a lower mean accuracy (180.1 km compared to 236.1 km) than the previous traceability study on the species [38]. Following the author’s limit of samples correctly assigned within 500 km of their known origin, our approach correctly localized 96% of our samples compared to their 87.4% [38], with only seven out of 171 samples surpassing the 500 km limit. The higher accuracy can be attributed to our bolstered geographic representation of the geo-referenced DNA database and the added statistical power of integrating more SNPs within the gene-capture approach (up to 8,520) compared to Tinsman, Gruppi [38] 96 SNP assay. Previous DNA-based trade tracing for Sunda and Chinese pangolins has focused on population-level assignments [39,40]. Here, we present the first empirical assessment of individual-level geographic assignment accuracy for both species. While limited geo-referenced samples—particularly for the Chinese pangolin—reduced assignment precision of Asian pangolins compared to the white-bellied pangolin, our method is still more accurate than prior population-based approaches (Sunda: 44.44% within 500 km, 100% within 1,000 km; Chinese: 33.33% within 500 km, 66.67% within 1,000 km), offering improved resolution of trade dynamics.

In systems dominated by clines or broad contact zones, as was the case for the Sunda pangolin, population-level assignment will inherently show lower accuracy. Continuous-space methods such as those implemented in our study (i.e., Locator) are therefore more suitable for tracing individuals to regions of origin, provided that these clinal regions are well sampled. Our method also permitted the detection of inaccuracies in the provided geolocations of reference samples by identifying regions with higher hold-out predictive error. For instance, the DRC for the white-bellied pangolin had a higher mean prediction error, which we posit may be the consequence of lower sampling representation and geolocation uncertainties in early museum collections due to town and region naming ambiguities in parts of the country [68]. Using LOOCV, we also detected variability in predictive error across samples, with clear outliers observed across all three species. This may also result from variation in the precision and reliability of reported geographic information across reference samples and their site-types, thereby affecting prediction accuracy. By layering multiple sample site-types (fresh field, restricted-range market, and museum samples; see S1 Text), we were able to identify and mitigate site-type biases. However, additional sampling in regions dominated by a single site-type, missing reference samples, and reference samples with high predictive error as a result of unreliable reporting of geographic information will help strengthen future predictive power.

### Tracing the trade of the three most trafficked pangolin species

We provide, for the first time, a global snapshot-in-time of trafficking hotspots across the ranges of the three most trafficked pangolin species using an empirical, individual-level geographic assignment approach [44]. Our study is unique in the sense that we distinguished data from local sources (field sites, roadside, and village vendors), regional domestic markets (bushmeat trade hubs), and international seizures. This offers insight into the dynamics between domestic trade for mainly consumptive purposes, such as the bushmeat trade [25], and international trade in scales driven by large traditional medicine industries and often destined for China and Vietnam [47]. We found that major long-range markets in both Africa and Asia are exclusively regionally sourced. An expected outcome given that these often openly tolerated markets sell bushmeat to local clientele for sustenance [69], limiting the financial incentives for transportation over vast geographic distances. In contrast, sourcing for international seizures was more widely distributed and followed a regional procurement pattern prior to shipping, suggesting considerable connectivity between traffickers operating in different countries [38,70]. Until now, it remained unclear whether these represent interconnected supply chains or distinct networks with separate actors [25]. Our dense sampling of the white-bellied pangolin in Cameroon—a major hub for pangolin trafficking [38]—revealed that both forms of trade (domestic and international) draw from the same source regions. This overlap is also found, albeit less pronounced, in the two Asian species and suggests a shared supply chain and common actors at the point of pangolin harvest, and possibly beyond. Without a comprehensive understanding of how local trade feeds international networks, legal provisions aimed at enabling local ‘sustainable’ use of pangolins [71] could unintentionally bolster the same trafficking networks driving their declines and undermine current conservation efforts.

Our study confirms previous broader-scale population-level assignments of the white-bellied pangolin, whereby the Lower Guinea cluster (eastern bank of Niger to the western bank of Congo River) is a major contributor to the international trade [33,34,38]. Using individual-level geographic assignment of the international trade, we confirm that southwestern Cameroon in particular, is the major global hotspot for trafficking white-bellied pangolins [38]. We identify key sourcing regions around Campo Ma’an National Park and Ebolowa’s restricted-range market (sourcing from surrounding forests), which are known sources for large bushmeat markets [25,35,72]. The Littoral Region in western Cameroon was an additional area feeding the international trade, particularly around the Ebo Forest Massif (Yabassi), where illegal forestry and its connections with wildlife trade have been reported [35,72].

Domestic traditional medicine and bushmeat market hubs selling white-bellied pangolins were highly localized in their sourcing of pangolin products (averaging 136 km between predicted source and market), a pattern not necessarily common for other species in the region [70,73]. Yaoundé, the largest regional bushmeat trade hub in Cameroon, sourced the majority of its pangolin products from the same areas as the international trade (southwestern and western Cameroon), reflecting previous research on the bushmeat trade using microsatellites [35]. We found sourcing from Cameroon’s Centre and Littoral Regions, but no evidence of cross-border trade from Equatorial Guinea [35]. The Douala bushmeat market, Cameroon’s second-largest, appeared more geographically confined, primarily sourcing from around the Ebo Forest Massif. In both markets, Din Dipita, Missoup [35] suggested similar trends but at a broader geographic scale, likely due to the lower resolution of microsatellites. Yaoundé may be using subsidiary markets in Sangmelima, Ebolowa, Eseka, Yabassi, and potentially Douala as part of its pangolin supply chain, considering the common sourcing areas across these markets. The interplay between regional markets (large cities and crossroads) and localized sources (roadside and village markets near forested areas) suggests that local hunting is partly driven by broader economic incentives from the domestic bushmeat trade. Such patterns should provide insight for population and hunting management at local to country-wide scales [74,75].

We identified several main regions feeding the large urban markets across Africa, including central Côte d’Ivoire, western Ghana, eastern Togo, and central Benin, which may constitute geographic hotspots of the pangolin trade (also see: [36,37]). Cross-border trade was detected between several neighboring countries, possibly facilitated by shared ethnic ties and established trade networks (e.g., Côte d’Ivoire-Ghana, Togo-Benin; see: [36,37]). We also found evidence of maritime trade from Cameroon to Bioko Island (Equatorial Guinea), supporting the hypothesis of a “commercial extinction” of white-bellied pangolins on the island, where populations may have been significantly depleted in areas accessible to hunters [25].

Regarding Sunda pangolins, large markets in Sumatra and Java indicate substantial intra-island sourcing, particularly those in Medan, Jember, and Surabaya. We confirm that Java, especially the large ports near Jakarta and Surabaya, serves as a major regional distribution hub, primarily receiving pangolins from Borneo [40], and mirroring patterns observed in other traded species [76,77]. Our findings also highlight the transnational nature of trade into China, with most individuals in Chinese seizures originating outside the country, particularly from Myanmar and the broader continental Southeast Asia. The widespread sourcing associated with seizures in Guangzhou contrasts with the more regionally focused patterns in Yunnan, suggesting differences in market demand, supply networks, or enforcement pressure between these regions. The apparent role of the Sino-Burmese border, especially the Lincang area, as both a sourcing and transit point underscores its strategic importance in trafficking flows. This aligns with prior accounts of the area serving as a key point for wildlife trade from Thailand and India into China [78].

We did not find Sunda pangolin individuals from Indonesian and Malaysian islands in the Chinese seizures we had access to, despite the majority of Indonesian-Malaysian sourcing said to be destined for China [47,79]. It is plausible that major Chinese cities we could not sample—such as Bozhou, Xiamen, Fuzhou, and Hong Kong—serve as key endpoints for Sunda pangolins sourced from Indonesia and Malaysia, given their links to traditional medicine and shipping networks. However, both Indonesia and Malaysia also maintain domestic demand through their own bushmeat and traditional medicine markets, necessitating further research on supply chain dynamics within and beyond these countries.

Despite more limited resolution in tracing Chinese pangolin trade, our results identify the significance of the species’ far western range—particularly India, Nepal, and Myanmar—as a source for Yunnan and the Sino-Burmese border area. This supports earlier evidence of established cross-border trafficking routes around the Sino-Burmese border with the Sunda pangolin and other species [80,81]. Assam, India, emerges as a notable source, with multiple individuals likely smuggled via Myanmar’s Sagaing Region, a known transit corridor [81]. Seizures in Kolkata and Jalpaiguri, India, indicate the same source region along the border with Myanmar, suggesting they could be end-user markets or transit points for China. There is also an indication of within-country sourcing (mainly around Yunnan), particularly from seizures in Guangdong, both of which are said to be important trade regions [79]. Previous studies using traditional trade tracing approaches (interviews and trade seizure shipping data) indicated transit links alongside the border of southwestern China (Guangxi and Hunan), and key south-eastern provinces as trade hubs (Fujian and Zhejiang), which we did not identify due to a lack of access to seizures in these regions [29,79,81]. These traditional trade-tracing studies cannot always clarify which pangolin species were involved; therefore, we question whether these are Chinese pangolin-specific trends.

A key limitation of this and previous studies is the sparse spatiotemporal sampling of international seizures, which constrains our understanding of global trade dynamics. We also did not analyze seizures over time, meaning these predicted hotspots are snapshots of trade dynamics that may already have evolved as traffickers seek new source regions to maintain supply. Accessing seizures from across the globe requires extensive collaborative networks, logistics, and resources. To better identify trade routes and track their evolution, we therefore advocate for the global implementation of standardized genotyping protocols for further development of geo-referenced DNA databases targeting field-based samples for all pangolin species. Achieving this will require sustained collaboration among pangolin trade tracing initiatives worldwide, with the aim of transferring existing protocols and co-developing new ones with range-state authorities.

## Materials and methods

### Bait design for gene-capture across African and Asian pangolins

The bait design procedure aimed to generate RNA probes capable of capturing all eight currently recognized pangolin species across a wide range of DNA qualities. It also needed to target genomic regions that would provide high-resolution tracing. To do this, a bioinformatics workflow was designed and implemented to produce a short-list of the most informative loci (*n* = 671) accounting for relative evolutionary rates (RER, *n* = 414), and biological relevance (BR, *n* = 257; functionally informed genes that may have geographically linked signatures) using Geneious 9.1.8 (https://www.geneious.com; S17 Fig and S1 Text). This resulted in a custom myBaits kit v5 (Daicel Arbor Biosciences) of 671 loci with 40 K probes at 3× tiling (every 27nt) and 70mer baits (*ca.* 1.2mb with 627,432 bp for Asian and 607,461 bp for African clades). The kit was designed on a reference species from each continent (white-bellied pangolin for Africa and Sunda pangolin for Asia) and tested both *in silico* (read-mapping to baits) and in vitro (capture) on all eight species. This meant that the probes would be able to target species from both continental clades in a single reaction mix (the two references), be capable of hybridizing with highly degraded museum samples (70mer baits), have limited gaps in coverage across the loci of interest (3× tiling), and target fast-evolving and geographically relevant loci for fine-tuned tracing. For more details on the bait design and probe development, see S1 Text. The baits’ references can be found in the Zenodo archive (https://doi.org/10.5281/zenodo.19286247).

### Sample collection and metadata

A total of 711 samples were collected from 15 museums (*n* = 122) and tissue collections (*n* = 589), distributed into 558, 118, and 35 samples for white-bellied (*P. tricuspis)*, Sunda (*M. javanica)*, and Chinese (*M. pentadactyla*) pangolins, respectively. Tissue collection samples were collected from various projects within the collaborative network, as part of ongoing or previous projects [35–37,40,50,82], for which details on sample collection are available. Museum samples were stored dry at room temperature, whereas all tissue collection samples were stored in 90% EtOH at 4 °C. We curated a database of sample metadata that included all information related to each sample from its source (S1 Appendix). In short, we collected information on sample localities and their coordinates, site-type (field site, restricted-range market, long-range market, seizure, unknown), and whether it came from a museum (more details can be found in S1 Text). Differentiation between site-types are as follows: (i) field sites are isolated localities away from major settlements or transport routes; (ii) restricted-range markets are small towns where trade is likely supplied locally from surrounding forests; (iii) long-range markets are large cities or transport hubs connected by major routes and associated with regional to cross-border trade; (iv) seizures are samples confiscated by enforcement authorities or collected outside species’ native ranges, indicating international trafficking; and (vi) unknown are samples lacking sufficient locality information for reliable geographic assignment. Both field sites and restricted-range markets were used as geographic references for provenance testing of the other three categories (see below). These were distributed as follows: trade seizures (*n* = 106 samples), long-range markets (*n* = 348), restricted-range markets (*n* = 161), field sites (*n* = 74), and unknown (*n* = 22).

### DNA extraction, gene-capture, and sequencing

Tissues were extracted using the NucleoSpin Tissue Mini Kit (Macherey-Nagel, Hoerdt, France) or DNeasy Blood & Tissue Kit (Qiagen, Hilden, Germany) from 4–25 mg of material, with the final elution step performed twice using 50 µL TE buffer. Extractions from museum bones and skins were carried out in a dedicated, physically isolated laboratory at the Pôle Biologie Moléculaire et Microbiologie facility (B2M, CRBE). Museum skins were rehydrated in 1× TE buffer and extracted following a chloroform-isoamyl alcohol protocol (CTAB), including dithiothreitol (DTT) [83]. Bones were reduced to powder (40–120 mg) using a liquid nitrogen–cooled mortar (Bel-Art, Wayne, NJ), extracted in an EDTA-based buffer, and purified using the QIAquick PCR Purification Kit (Qiagen) following Bennett, Massilani [84]. A negative control was included in each extraction series. We used Qubit 2.0 (fluorometric quantitation of dsDNA) and Agilent D5400 Fragment Analyzer (DNA size profiling) to measure DNA quality and yield after extractions for all samples. All DNA extracts were stored at −20 °C until further processing.

Genomic DNA libraries were prepared using the Illumina TruSeq Nano HT kit, whereby each sample was indexed using Illumina’s unique dual indexes. Variations in bead-based sizing and purification, as well as the number of cycles for enrichment PCR steps, were dependent on the level of DNA degradation. However, we aimed for a minimum DNA fragment length of 70 bp, which fitted the bait size (70mer baits) and allowed for the inclusion of heavily degraded libraries with small fragment sizes. Qubit 2.0 and Agilent D5400 Fragment Analyzer were again used to measure library yield and complexity before pooling samples at equimolar concentrations by 4–17 samples per pool for gene-capture. Samples that were heavily degraded were pooled in pools of 1–3 samples. A preliminary gene-capture run with four museum samples was used to test the best bait hybridization and washing temperatures. The temperatures were 63 °C (10%–15% bait-target divergence) and 65 °C (10% bait-target divergence), whereby 65 °C was chosen based on a higher capture efficiency (83.57% compared to 64.57% average capture efficiency—percent of reads sequenced to reads mapped to the target region).

We then used the myBaits v5 (Daicel Arbor Biosciences) standard protocol to capture our regions of interest at the aforementioned bait hybridization and washing temperatures. Incubation times for bait hybridization (16–24 hours) and capture PCR amplification cycles (12×–16×) varied depending on library concentrations. Post-capture PCR enrichment was performed with KAPA HiFi HotStart ReadyMix and xGen Library Amplification Primers (IDT), whereby each post-capture pool was aliquoted into two parts for enrichment and then re-pooled after enrichment in order to increase DNA yields per capture reaction without high levels of PCR duplication [18]. Enriched, post-captured pools were purified using cleanNGS (CleanNA) purification beads at a 1:1 bead-sample ratio. Qubit 2.0 and KAPA SYBR FAST qPCRs were used to measure post-capture pool yield before pooling captured libraries by equimolar concentrations for paired-end sequencing with Illumina MiSeq Nano/ MiSeq 2 next-generation sequencers (150/250 bp read lengths depending on initial sample quality). To guide further laboratory processing and sequencing efforts, we employed a simple pipeline aimed at obtaining summary statistics and testing pooling limits (see S1 Text for more details). Capture efficiency and depth of coverage were not affected by pooling of up to 12 samples per capture reaction (S1D and S1E Fig). Due to limited gene-capture reactions and pooling of libraries upstream of capture reactions, technical replicates were not generated, preventing replicate-based benchmarking.

All gene-captured raw sequences have been deposited in the European Nucleotide Archive (ENA) at EMBL-EBI under accession number PRJEB93883 (https://www.ebi.ac.uk/ena/browser/view/PRJEB93883), whilst the two bait references have been deposited in the following Zenodo Archive (https://doi.org/10.5281/zenodo.19286247).

### Gene-capture sequencing processing with EAGER

For the following bioinformatic analyses, we used the bait design loci of *M. javanica* (for *M. javanica* and *M. pentadactyla* samples) and *P. tricuspis* (for *P. tricuspis* samples) as fasta references, whereby each locus is treated as an independent reference sequence to facilitate mapping and variant calling. These references were indexed with SAMtools v1.10 *faidx* [85]. A mitochondrial genome was added to each reference (KT445979 for *M. javanica* and KP306514 for *P. tricuspis*) as an additional independent reference sequence to obtain mitogenomes from mtDNA amplicons that were sequenced as a by-product [86,87].

For sequencing processing, we employed the nf-core platform’s EAGER v2.4.5 pipeline [88,89], a comprehensive bioinformatics pipeline for read pre-processing, mapping, and genotyping that is tailored to ancient DNA [90]. We applied the pipeline to the point before genotyping, after which we followed our genotyping protocols (see below). Pipelines were run separately for Asian (*M. javanica* baits reference) and African (*P. tricuspis* baits reference) species and differed depending on whether samples were from museums or not. We used a TSV (tab-separated values) input for each run, which meant merging sequencing data across lanes, sequencing runs, sequencing configuration types, and samples where necessary. Each of the steps performed is described briefly below.

Sequencing quality was assessed with FastQC v0.119 before and after undergoing cleaning and read merging (collapsing) with fastp v0.20.1 [91] and AdapterRemoval v2.3.2. Cleaned reads were mapped to their respective bait reference using the BWA-MEM algorithm of BWA v0.7.17 [85], followed by filtering with SAMtools v1.12 at a mapping quality threshold of >30 Phred. Removal of PCR amplicon duplicates was completed by Picard v2.26.0 *MarkDuplicates* and library complexity was estimated with Preseq v3.1.1 [92]. To assess DNA damage, DamageProfiler v0.4.9 [93] was implemented, whereby we set the length filter option (-l) to 70 bp due to the lower sizing bounds of our sequencing libraries. For museum samples, we included the option *run_mapdamage_rescaling* that implements mapDamage2 v2.2.1 [94] BAM rescaling (probabilistically reverts T’s to C’s depending on the likelihood of damage-induced reference-sample mismatch). SAMtools v1.12 *flagstat* was implemented before and after mapping filtering, while Qualimap v2.2.2 [95] was used to extract mapping statistics post-filtering and cleaning.

MTNucRatioCalculator v0.7 was used to calculate mtDNA to nDNA ratios per de-duplicated library, whilst EndorS.py v0.04 provided levels of endogenous DNA off flagstat outputs [88]. Finally, all aforementioned quality estimates and pipeline statistics were combined per run using MultiQC v1.13 (See S1 Appendix for basic statistics per sample) to help guide the influences of contamination and degradation on genomic insights [96].

### Published genome processing

In order to include all available genomic data on the three species, short-read data from previously published sources were downloaded. This included genomic data from *M. javanica* (*n* = 57), *M. pentadactyla* (*n* = 16), and *P. tricuspis* (*n* = 19), representing key, distinct populations previously identified [21,39,41,97] or recently shotgun sequenced by our collaborative network (*n* = 4). As with the collected samples, they were designated as follows based on information from previously published articles: trade seizures (*n* = 80), long-range markets (*n* = 3), restricted-range markets (*n* = 1), and field sites (*n* = 8; S1 Appendix). Refer to S1 Text for details on the different site types and information for each seizure.

Short read data from all species were cleaned with fastp v0.19.4 using default parameters and then mapped to the respective baits reference using BWA v0.7.15. We used SAMtools v1.10 *view* to discard unpaired reads (flag -f 1), mapped reads with a low mapping quality (>30 Phred using -q30), and unmapped, secondary, and supplementary mapped reads (flag -F 2308). The reason for implementing this filtering method is due to mapping whole genome data on a reduced representation of the genome (the target region of the baits reference). This can result in the possibility of reads half-mapping to the end of a locus boundary and then being inaccurately mapped to other parts of this same locus (supplementary alignments) and reads inaccurately mapping to more than one locus (secondary alignments). The removal of PCR amplicon duplicates was completed by Picard v2.26.0 *MarkDuplicates*.

### Mislabeled species and duplicate sample cleanup

With the possibility of species mislabeling and duplicated collection of samples, we conducted a pre-phylogeny and PCA for the three species with representatives from all eight recognized and the ninth unrecognized species [21,22], as well as Pairwise relatedness estimates using the KING algorithm [98]. An individual from each pairwise relatedness comparison above 0.354 (duplicate/monozygotic twin) was removed from further analyses, whilst mislabeled individuals were correctly re-assigned to their species based on the continent-specific phylogenies and PCAs. Further details on these methods can be found in S1 Text.

### Genotyping and filtering for per-species analyses

Once sample duplicates and mislabeling were corrected, BCFtools v1.8 was again used to determine per-species genotype likelihoods from all cleaned bam files using *mpileup*, call for variants using *call*, and normalize those variants using *norm*. Each species-specific resultant vcf/SNP file was indexed with BCFtools v1.8, before extracting various variant statistics (mean depth per site, allelic frequencies, site missingness, and individual missingness) with VCFtools v0.1.15 [99]. These estimates were plotted with ggplot2 [100] in order to guide filtering with VCFtools v0.1.15. This included removing indels, sites under 8× average depth, a species-specific maximum site depth (35×–75×), sites with more than 30% missing data, variant quality below Phred 20, and a minor allele count of 3. Variants under selection (non-neutral) from the resultant per-species filtered SNP files were identified using Tajima’s D estimates (>2 & >−2) in VCFtools v0.1.15. These files were then split into two partitions: (i) nDNA loci with neutral SNPs only, and (ii) nDNA loci with all SNPs (neutral and non-neutral). Both the neutral and full nDNA SNP files for each species underwent linkage disequilibrium pruning with PLINK v2.0 [101] by removing SNPs within a 50 SNP sliding window, shifting 10 SNPs per step, that had *r*^2^ > 0.2. During this step, all individuals with more than 75% missing data were excluded.

Although the bait panel was designed from both RER and BR loci, subsequent genotype filtering (depth, missingness, MAC, variant quality, and LD pruning) was applied uniformly across all loci, meaning that the final SNP dataset does not retain clean RER/BR partitions. For this reason, downstream population and assignment analyses were performed using the full set of retained SNPs partitioned as neutral and non-neutral. This framework was chosen to maximize robustness across degraded samples and maintain compatibility with methods such as ADMIXTURE and Locator.

### Population stratification

The linkage pruned files containing all the nDNA SNPs (neutral and non-neutral) of each species were used to create PCAs using PLINK v2.0 and ggplot2. All individuals with more than 75% missing data were excluded as an initial filter as PLINK v2.0 corrects for missingness when estimating the genetic relationship matrix. All SNPs were used as the idea was to stratify the populations as much as possible by including both neutral genetic distance (neutral SNPs) and potential geographical adaptation (non-neutral SNPs).

The linkage-pruned files containing solely bi-allelic (filtered with PLINK v2.0), and neutral nDNA SNPs for each species were used for population structuring with ADMIXTURE v1.3 [102]. This is due to ADMIXTURE’s assumptions of structuring through neutral loci (Hardy–Weinberg equilibrium and random mating) and our aim of determining a fixed population structure. Here, each *K* (1–10) was computed 10 times, with a cross-validation of 10 iterations, to test for variability among replicates for each *K* with CLUMPAK [103]. CLUMPAK found no genuinely distinct modes for each *K* of each species, and the best *K* per species was determined from cross-validation (Evanno estimates). Admixture/structure plots were created in Rstudio v4.2.1 following a custom R script (see the Zenodo Archive) after merging sample metadata with the qmatrix from ADMIXTURE. The following map layers and their sources were used for mapping the genotypes: global ecological land units [104], global elevation (GlobalSolarAtlas-v2; https://globalsolaratlas.info), and 1:50m scaled water bodies and country boundaries (Natural Earth—https://www.naturalearthdata.com/). Species range polygon base layers were estimated from georeferenced occurrence records of the three species (https://data.nhm.ac.uk/dataset/natalie-cooper; [43]) using concave hull (alpha hull) modeling following removal of geographic outliers and restriction to terrestrial landmasses (S1 Text).

Using the ADMIXTURE SNP dataset for each species, IBD was assessed with a Mantel test (1,000 permutations) implemented in the R package adegenet v2.1.10 [105]. This was used to test the relationship between pairwise Euclidean genetic distances derived from allele frequencies and geographic distances based on sampling coordinates, which were determined using the R package fossil [106]. Before analyses, seizure and long-range market samples were excluded to avoid confounding results from inaccurate coordinates.

To distinguish between continuous genetic variation driven by IBD and discrete admixture between divergent populations, triangle plot analyses were performed using the R package triangulaR v.0.0.1 following package defaults [107]. These analyses were performed using highly differentiated SNPs (allele frequency difference ≥0.9) between predefined reference populations (from PCA and ADMIXTURE analyses) representing genetic extremes within each species.

### Geographic origin assignment

To determine the geographic origins of samples of unknown locality (traded individuals), Locator [44] was implemented using the combined full nDNA (neutral and non-neutral SNPs) and mtDNA vcf files for each species. Locator is a deep-learning approach whereby it analyzes the genotypes and geo-referenced locations for individuals of known localities to train a neural network to approximate the relationship between them. Training is conducted by randomly splitting samples of known localities into a training set (model parameter fitting) and a validation set (used to tune the parameters and evaluate errors after training). Once optimized, it then predicts locations for the set samples held out from the training, the samples of unknown localities.

Samples from field sites and restricted-range markets were used as reference samples (the known localities), whereby trade tracing was conducted on the samples from long-range markets, seizures, and samples of unknown origins due to missing sampling information (the unknown localities). Details on sampling in restricted-range and long-range markets can be found in previous studies [35–37], which report on informal interviews with market vendors regarding information on sources (see also S1 Appendix). Details of each seizure are provided in S1 Text. Training (known locality) and prediction (unknown locality) sample sets were split as follows for each species: *P. tricuspis* (171 training, 304 predicted), *M. javanica* (22 training, 129 predicted), *M. pentadactyla* (16 training, 27 predicted). We used the most accurate method for Locator on our samples by running 100 bootstrap replicates (running an entirely new model for each replicate and measuring inter-model accuracy between predicted locations). To prevent overfitting, training was performed with dropout regularization (dropout = 0.25) and early stopping (training halted if validation loss did not improve for 100 epochs). To provide distance-based accuracy estimates (see: S1 Table), we used three complementary approaches. Firstly, we used a modified version of Locator’s Rscript (plot_locator.R; see the Zenodo Archive) [44] to measure the distance between the 100 bootstrapped predicted centroids per sample. This provides an estimate of prediction variability in kilometers. The other two approaches fall under a hold-out type of analysis, whereby reference samples are withheld from the training dataset (known localities) and treated as an unknown during prediction (prediction dataset) to measure the geographic distance between a sample’s true sampling location and its predicted location. This provided an estimate of the error in the prediction power in kilometers. The first hold-out analysis involved placing around one-third of the samples of known localities from across the range of each species in the unknown locality set (to predict), running the Locator model through 100 bootstrap replicates, and measuring the distance between the predicted centroid and the known sampling locality of each of the samples (named the one-third hold-out analysis). The second hold-out analysis was a LOOCV. Here, we iteratively held out each referenced sample (known locality) and re-trained the Locator model each time until all reference samples were withheld per run. Due to the computational intensity of the task, we did not perform 100 bootstrap retrainings per held-out sample run.

The one-third hold-out predictive error approach provides a measure of robustness to overall data loss and is less sensitive to single-sample bias, but provides a coarse estimate of accuracy [108]. The LOOCV predictive error is sensitive to local error patterns by providing a measure per reference sample, but can be strongly affected by sparse regional sampling [108].

Using a custom Rscript (see the Zenodo Archive), the 100 predicted locations for each sample were averaged to identify its centroid, which was then used to plot a traceability map connecting the point of seizure to a predicted, centroidal location for each sample using ggplot2 [100]. We used the *stat_density2d* command in ggplot2 for 2D kernel density estimation of all 100 bootstrapped runs across all samples for each species as a means of providing a visual representation of model accuracy at a per-species level. The following map layers and their sources were used to enrich the maps: protected areas (World Database on Protected Areas—https://www.protectedplanet.net), 1:10m scaled roads, ports, and country boundaries (Natural Earth—https://www.naturalearthdata.com/), and the modeled species’ range from occurrence data previously collated (https://data.nhm.ac.uk/dataset/natalie-cooper; [43]; S1 Text). To calculate distances between the predicted, centroidal location and the sampling location, we used the *distHaversine* command in the geosphere package [109]. This provides an estimate of the trade distances per species in kilometers.

## Supporting information

S1 Fig Gene-capture summary statistics. Box and whisker plots of capture efficiency across **(A)** species and **(B)** quality (museum vs. fresh) with X representing the mean and outliers shown as points. **(C)** A scatter-line plot of the proportion of gene-capture target region covered at various depths of coverage for each species (lines indicate the mean and shaded areas indicate 95% confidence intervals). Scatter plots comparing the number of individuals pooled in a single capture reaction to **(D)** capture efficiency and **(E)** sequencing depth. The underlying numerical data are provided in S1 Data.
(TIF)

S2 Fig Reference database map. Sampling locations of both samples from this study and previously published studies for all three species. Reference samples are field-site and restricted-range market samples used as the reference set for trade tracing (known localities). Non-reference samples are those from unknown localities, including long-range markets, international trade seizures, and samples with missing sampling locality information. These samples represent individuals that were gene-captured, sequenced, and SNP-called, and do not necessarily correspond to the subsets used in downstream analyses, which applied method-specific filtering thresholds. Base layers include country data from Natural Earth (http://www.naturalearthdata.com) and the modeled species’ range from occurrence data previously collated (https://data.nhm.ac.uk/dataset/natalie-cooper; [43]). See S1 Appendix for detailed information on each sample (and underlying numerical data) and S1 Text for a breakdown of different site types.
(TIF)

S3 Fig White-bellied pangolin (*Phataginus tricuspis*) clustering bar graphs of all *K*s (*K* = 2–10). Samples belonging to countries are clumped, and each site type is labeled (8,312 SNPs, 456 individuals). Samples are ordered from the South-East to the North-West in each country. The underlying numerical data are provided in S1 Data.
(TIF)

S4 Fig Principal component analysis (PCA) of the white-bellied pangolin (*Phataginus tricuspis*) zoomed in on the top two axes of differentiation. Points are colored by country and shaped by type of site from which the sample was collected (8,520 SNPs, 427 individuals). These represent the three major clades of the species **(A)** central-western Africa, **(B)** central Africa, and **(C)** western Africa. The underlying numerical data are provided in S1 Data.
(TIF)

S5 Fig Principal component analysis (PCA) of the white-bellied pangolin (*Phataginus tricuspis*) for the top four axes of differentiation. Points are colored by country and shaped by type of site from which the sample was collected (8,520 SNPs, 427 individuals). The underlying numerical data are provided in S1 Data.
(TIF)

S6 Fig Isolation-by-distance and triangle-plot analyses. Isolation-by-distance (IBD) plots comparing pairwise genetic divergence (Euclidean distance) with pairwise geographic distance (km) across all individuals from each of the three pangolin species: **(A)** white-bellied (*Phataginus tricuspis*), **(C)** Sunda (*Manis javanica*), and **(E)** Chinese (*Manis pentadactyla*) pangolins. Triangle plots used for discriminating IBD-like clines from genuine admixture across the species’ range for **(B)** white-bellied (*Phataginus tricuspis*), **(D)** Sunda (*Manis javanica*), and **(F)** Chinese (*Manis pentadactyla*) pangolins. Reference populations represent the extremes of each species’ range. The underlying numerical data are provided in S1 Data.
(TIF)

S7 Fig White-bellied pangolin (*Phataginus tricuspis*) colorblind friendly version of Fig 1. Samples belonging to countries are clumped, and each site type is labeled (8,312 SNPs, 456 individuals). Samples are ordered from the South-East to the North-West in each country. A color key is provided to distinguish between the colors and populations indicated in the main text and this figure. Base layers include country and river data from Natural Earth (http://www.naturalearthdata.com; darker blue indicates larger rivers), elevation from GlobalSolarAtlas-v2 (https://globalsolaratlas.info, in deep red), and the modeled species’ range from occurrence data previously collated (https://data.nhm.ac.uk/dataset/natalie-cooper; [43]). The underlying numerical data are provided in S1 Data.
(TIF)

S8 Fig Principal component analysis (PCA) of the Sunda pangolin (*Manis javanica*). This is from 4,418 SNPs and 149 individuals for the **(A–C)** top four axes of differentiation. Points are colored by country and shaped by type of site from which the sample was collected. **(D)** PCA with the top two axes of differentiation in which points are colored by previously identified clusters [39,40] and shaped by type of site from which the sample was collected (NA represent samples that come from this study and thus did not yet have a cluster). The underlying numerical data are provided in S1 Data.
(TIF)

S9 Fig Sunda pangolin (*Manis javanica*) clustering bar graphs of all *K*s (*K* = 2–10). Samples belonging to countries are clumped, and each site type is labeled (4,162 SNPs, 148 individuals). Samples are ordered from South-East to North-West in each country. The underlying numerical data are provided in S1 Data.
(TIF)

S10 Fig Sunda pangolin (*Manis javanica*) colorblind friendly version of Fig 2. Samples belonging to countries are clumped, and each site type is labeled (4,162 SNPs, 148 individuals). Samples are ordered from South-East to North-West in each country. A color key is provided to distinguish between the colors and populations indicated in the main text and this figure. Base layers include country and river data from Natural Earth (http://www.naturalearthdata.com; darker blue indicates larger rivers), elevation from GlobalSolarAtlas-v2 (https://globalsolaratlas.info, in deep red) and the modeled species’ range from occurrence data previously collated (https://data.nhm.ac.uk/dataset/natalie-cooper; [43]). The underlying numerical data are provided in S1 Data.
(TIF)

S11 Fig Chinese pangolin (*Manis pentadactyla*) clustering bar graphs of all *K*s (*K* = 2–10). Samples belonging to countries are clumped, and each site type is labeled (816 SNPs, 39 individuals). Samples are ordered from South-East to North-West in each country. The underlying numerical data are provided in S1 Data.
(TIF)

S12 Fig Chinese pangolin (*Manis pentadactyla*) colorblind friendly version of Fig 3. Samples belonging to countries are clumped, and each site type is labeled (816 SNPs, 39 individuals). Samples are ordered from South-East to North-West in each country. A color key is provided to distinguish between the colors and populations indicated in the main text and this figure. Base layers include country and river data from Natural Earth (http://www.naturalearthdata.com; darker blue indicates larger rivers), elevation from GlobalSolarAtlas-v2 (https://globalsolaratlas.info, in deep red) and the modeled species’ range from occurrence data previously collated (https://data.nhm.ac.uk/dataset/natalie-cooper; [43]). The underlying numerical data are provided in S1 Data.
(TIF)

S13 Fig Violin and boxplots of accuracy of origin tracing per species across accuracy metrics. **(A)** White-bellied (*Phataginus tricuspis*), **(B)** Sunda (*Manis javanica*), and **(C)** Chinese (*Manis pentadactyla*) pangolins. Violin plots show the distribution of prediction distances (km) across three metrics: leave-one-out cross-validation (LOOCV) predictive error, one-third hold-out predictive error, and prediction variability (bootstrap centroid-based dispersion). The median is indicated by the central line, and the interquartile range (IQR) by the boxplot. Individual points represent per-sample distances (shown for hold-out and LOOCV only due to the large sample size for prediction variability across the 100 bootstraps). The underlying numerical data are provided in S1 Data.
(TIF)

S14 Fig Locator predictive error maps after removing around one-third of the geo-referenced database. **(A)** White-bellied (*Phataginus tricuspis*), **(B)** Sunda (*Manis javanica*), and **(C)** Chinese (*Manis pentadactyla*) pangolins. Black circles indicate the most likely centroidal origin across 100 bootstrap replicates of each sample, with the gray line connecting it to its true origin location (where it was collected). Training sample locations (field collected) are indicated by red circles (for each case, the larger the circle size, the more training samples). See S1 Table for more details. The base layer country data is from Natural Earth (http://www.naturalearthdata.com). The underlying numerical data are provided in S1 Data.
(TIF)

S15 Fig Boxplots of distances between seized and sourced locations predicted by Locator. **(A)** White-bellied pangolin (*Phataginus tricuspis*) by cluster. **(B)** White-bellied pangolin (*P. tricuspis*) different site-types (seizures are not an accurate representation as these actually come from Europe and Asia). **(C)** Sunda pangolin (*Manis javanica*) different site-types. **(D)** Chinese pangolin (*Manis pentadactyla*) different site-types. The underlying numerical data are provided in S1 Data.
(TIF)

S16 Fig Origin tracing of traded white-bellied pangolins (*Phataginus tricuspis*) across the species’ range. Training locations (red diamonds); seized samples (black circles) linked to their inferred origins (gray crosses) via gray (domestic) lines. Protected areas (green), roads (orange), and species range (purple shading) are shown for context. The heatmap highlights likely sourcing density, with red indicating hotspots and heatmap bounds reflecting bootstrap confidence. Base layers include country and port (blue triangles) data from Natural Earth (http://www.naturalearthdata.com), protected areas (green) from the World Database on Protected Areas (https://www.protectedplanet.net), and the modeled species’ range from occurrence data previously collated (https://data.nhm.ac.uk/dataset/natalie-cooper; [43]). The underlying numerical data are provided in S1 Data.
(TIF)

S17 Fig The pipeline designed and implemented to create the final list of loci necessary for gene-capture. Based on the *Manis javanica* (GCF_001685135.1) and *Phataginus tricuspis* (GCA_004765945.1) reference assemblies, as well as OrthoMaM v10 database of orthologous genes.
(TIF)

S1 Table Accuracy of origin tracing per species. Model validation performance statistics are the model outputs from Locator, which provides the correlation between predicted and true locations for the validation samples used in model training (this is a measure of model fit). The lower the mean error (close to 0), the closer the validated sample predictions are from the training samples, and the higher the *R*^2^ values (*x* = longitude and *y* = latitude), the less the prediction will collapse towards the mean of each axis. Prediction variability is the distance between the centroid of all 100 bootstrap predictions and each bootstrapped predicted location when using all reference samples (the smaller the distance, the less variability in predictions, and thus the higher the confidence). Predictive error (or hold-out analysis) is defined as the geographic distance between a sample’s true sampling location and its predicted location when that reference sample is excluded from model training and treated as an unknown during prediction (smaller distances indicate higher spatial precision when reference data are withheld). We conducted two complementary hold-out approaches to measure predictive error: (i) a one-third hold-out, in which approximately one-third of reference samples distributed across the species’ range were removed in a single run and predicted, and (ii) Leave-One-Out Cross-Validation (LOOCV), in which each reference sample was iteratively removed per run, the model retrained, and the prediction error recorded for each sample until all reference samples have been removed at least once. Ref hold-out refers to the number of reference samples held out when conducting the one-third hold-out analyses of predictive error.
(DOCX)

S2 Table Trade distances in kilometers (km) for each species and site type. Distances are calculated between the centroidal location of 100 bootstraped predictions and the sampling location. Sampling locations are separated into site types: long-range markets (regional to cross-country commercial trade linked by major routes and within major cities), seizures (stated as seizures by authorities or those collected outside home-range countries whereby they formed part of an international trade), unknown localities (did not have accurate enough sampling localities to be given a field-site type, usually museum specimens). Seizure estimates are not accurate for the white-bellied pangolin (*Phataginus tricuspis*) since they were either in Europe or China.
(DOCX)

S1 Appendix Sample metadata for individuals used in this study. The spreadsheet is divided into two sheets: one detailing the samples collected, extracted, gene-captured and sequenced in this study, and the other detailing the whole-genome sequencing data used from previous studies. Metadata includes sample IDs, species, sampling information, localities, collectors or previous study authors, and basic gene-capture statistics for each sample.
(XLSX)

S1 Text Additional information on materials and methods. (DOCX)

S1 Data Underlying numerical data for figures. The underlying data has been broken up across multiple sheets (numbered as figure numbers) in an Excel Workbook. The README sheet explains the connections between sheets and their respective figures, as well as any key functions used to produce the figure from the datasheet. Coding scripts used to produce these data, along with their key figures, have been included in the Zenodo Archive (https://doi.org/10.5281/zenodo.19286247).
(XLSX)

## Abbreviations

IBD: isolation-by-distance
IWT: illegal wildlife trade
LOOCV: Leave-One-Out Cross-Validation
NGS: Next-Generation Sequencing
PCA: Principal Component Analysis
SNP: Single-Nucleotide Polymorphism

## References

[1] UNODC. World wildlife crime report 2024: trafficking in protected species. Vienna: United Nations Office on Drugs and Crime; 2024.

[2] Hammer S, Watson R. The challenge of managing Spix Macaws (*Cyanopsitta spixii*) at Qatar – an eleven-year retrospection. Der Zoologische Garten. 2012;81(2–3):81–95. doi: 10.1016/j.zoolgart.2012.05.005

[3] O’Hanlon SJ, Rieux A, Farrer RA, Rosa GM, Waldman B, Bataille A, et al. Recent Asian origin of chytrid fungi causing global amphibian declines. Science. 2018;360(6389):621–7. doi: 10.1126/science.aar1965 29748278 PMC6311102

[4] Westphal MI, Browne M, MacKinnon K, Noble I. The link between international trade and the global distribution of invasive alien species. Biol Invasions. 2007;10(4):391–8. doi: 10.1007/s10530-007-9138-5

[5] Chowdhury EK, Khan II, Dhar BK. Catastrophic impact of Covid‐19 on the global stock markets and economic activities. Business Soc Rev. 2021;127(2):437–60. doi: 10.1111/basr.12219

[6] Allendorf FW. Genetics and the conservation of natural populations: allozymes to genomes. Mol Ecol. 2017;26(2):420–30. doi: 10.1111/mec.13948 27933683

[7] Hohenlohe PA, Funk WC, Rajora OP. Population genomics for wildlife conservation and management. Mol Ecol. 2021;30(1):62–82. doi: 10.1111/mec.15720 33145846 PMC7894518

[8] Mussmann SM, Douglas MR, Anthonysamy WJB, Davis MA, Simpson SA, Louis W, et al. Genetic rescue, the greater prairie chicken and the problem of conservation reliance in the Anthropocene. R Soc Open Sci. 2017;4(2):160736. doi: 10.1098/rsos.160736 28386428 PMC5367285

[9] Willoughby JR, Christie MR. Long-term demographic and genetic effects of releasing captive-born individuals into the wild. Conserv Biol. 2019;33(2):377–88. doi: 10.1111/cobi.13217 30168872

[10] Ogden R, Linacre A. Wildlife forensic science: a review of genetic geographic origin assignment. Forensic Sci Int Genet. 2015;18:152–9. doi: 10.1016/j.fsigen.2015.02.008 25795277

[11] Wasser SK, Brown L, Mailand C, Mondol S, Clark W, Laurie C, et al. CONSERVATION. Genetic assignment of large seizures of elephant ivory reveals Africa’s major poaching hotspots. Science. 2015;349(6243):84–7. doi: 10.1126/science.aaa2457 26089357 PMC5535781

[12] Martinsohn JTh, Ogden R. FishPopTrace—developing SNP-based population genetic assignment methods to investigate illegal fishing. Forensic Sci Int Genet Suppl Ser. 2009;2(1):294–6. doi: 10.1016/j.fsigss.2009.08.108

[13] Ng CH, Ng KKS, Lee SL, Zakaria N-F, Lee CT, Tnah LH. DNA databases of an important tropical timber tree species *Shorea leprosula* (Dipterocarpaceae) for forensic timber identification. Sci Rep. 2022;12(1):9546. doi: 10.1038/s41598-022-13697-x 35680966 PMC9184630

[14] Nakahama N. Museum specimens: an overlooked and valuable material for conservation genetics. Ecol Res. 2020;36(1):13–23. doi: 10.1111/1440-1703.12181

[15] Andrews KR, De Barba M, Russello MA, Waits LP. Advances in using non-invasive, archival, and environmental samples for population genomic studies. Population Genomics. Springer International Publishing. 2018. p. 63–99. doi: 10.1007/13836_2018_45

[16] Eaton MJ, Meyers GL, Kolokotronis S-O, Leslie MS, Martin AP, Amato G. Barcoding bushmeat: molecular identification of Central African and South American harvested vertebrates. Conserv Genet. 2009;11(4):1389–404. doi: 10.1007/s10592-009-9967-0

[17] Derkarabetian S, Benavides LR, Giribet G. Sequence capture phylogenomics of historical ethanol-preserved museum specimens: unlocking the rest of the vault. Mol Ecol Resour. 2019;19(6):1531–44. doi: 10.1111/1755-0998.13072 31448547

[18] White LC, Fontsere C, Lizano E, Hughes DA, Angedakin S, Arandjelovic M, et al. A roadmap for high-throughput sequencing studies of wild animal populations using noninvasive samples and hybridization capture. Mol Ecol Resour. 2019;19(3):609–22. doi: 10.1111/1755-0998.12993 30637963

[19] Gooley RM, Tamazian G, Castañeda-Rico S, Murphy KR, Dobrynin P, Ferrie GM, et al. Comparison of genomic diversity and structure of sable antelope (*Hippotragus niger*) in zoos, conservation centers, and private ranches in North America. Evol Appl. 2020;13(8):2143–54. doi: 10.1111/eva.12976 32908610 PMC7463370

[20] Aylward ML, Sullivan AP, Perry GH, Johnson SE, Louis EEJ. An environmental DNA sampling method for aye-ayes from their feeding traces. Ecol Evol. 2018;8(18):9229–40. doi: 10.1002/ece3.4341 30377496 PMC6194247

[21] Gu T-T, Wu H, Yang F, Gaubert P, Heighton SP, Fu Y, et al. Genomic analysis reveals a cryptic pangolin species. Proc Natl Acad Sci U S A. 2023;120(40):e2304096120. doi: 10.1073/pnas.2304096120 37748052 PMC10556634

[22] Heighton SP, Allio R, Murienne J, Salmona J, Meng H, Scornavacca C, et al. Pangolin genomes offer key insights and resources for the world’s most trafficked wild mammals. Mol Biol Evol. 2023;40(10):msad190. doi: 10.1093/molbev/msad190 37794645 PMC10551234

[23] Wangmo LK, Ghosh A, Dolker S, Joshi BD, Sharma LK, Thakur M. Indo-Burmese pangolin (*Manis indoburmanica*): a novel phylogenetic species of pangolin evolved in Asia. Mamm Biol. 2025;105(5):691–8. doi: 10.1007/s42991-024-00475-7

[24] Shairp R, Veríssimo D, Fraser I, Challender D, MacMillan D. Understanding urban demand for wild meat in Vietnam: implications for conservation actions. PLoS One. 2016;11(1):e0134787. doi: 10.1371/journal.pone.0134787 26752642 PMC4709058

[25] Ingram DJ, Cronin DT, Challender DWS, Venditti DM, Gonder MK. Characterising trafficking and trade of pangolins in the Gulf of Guinea. Global Ecol Conserv. 2019;17:e00576. doi: 10.1016/j.gecco.2019.e00576

[26] Ingram DJ, Coad L, Abernethy KA, Maisels F, Stokes EJ, Bobo KS, et al. Assessing Africa‐wide pangolin exploitation by scaling local data. Conserv Lett. 2017;11(2). doi: 10.1111/conl.12389

[27] Challender DWS, Heinrich S, Shepherd CR, Katsis LKD. International trade and trafficking in pangolins, 1900–2019. Pangolins. Elsevier. 2020. p. 259–76. doi: 10.1016/b978-0-12-815507-3.00016-2

[28] Heinrich S, Wittmann TA, Prowse TAA, Ross JV, Delean S, Shepherd CR, et al. Where did all the pangolins go? International CITES trade in pangolin species. Global Ecol Conserv. 2016;8:241–53. doi: 10.1016/j.gecco.2016.09.007

[29] Omifolaji JK, Hughes AC, Ibrahim AS, Zhou J, Zhang S, Ikyaagba ET, et al. Dissecting the illegal pangolin trade in China: an insight from seizures data reports. NC. 2022;46:17–38. doi: 10.3897/natureconservation.45.57962

[30] Heighton SP, Gaubert P. A timely systematic review on pangolin research, commercialization, and popularization to identify knowledge gaps and produce conservation guidelines. Biol Conserv. 2021;256:109042. doi: 10.1016/j.biocon.2021.109042

[31] Gaubert P, Njiokou F, Ngua G, Afiademanyo K, Dufour S, Malekani J, et al. Phylogeography of the heavily poached African common pangolin (Pholidota, *Manis tricuspis*) reveals six cryptic lineages as traceable signatures of Pleistocene diversification. Mol Ecol. 2016;25(23):5975–93. doi: 10.1111/mec.13886 27862533

[32] Sitam FT, Salgado-Lynn M, Denel A, Panjang E, McEwing R, Lightson A, et al. Phylogeography of the *Sunda pangolin*, *Manis javanica*: implications for taxonomy, conservation management and wildlife forensics. Ecol Evol. 2023;13(8):e10373. doi: 10.1002/ece3.10373 37593756 PMC10427774

[33] Ewart KM, Lightson AL, Sitam FT, Rovie-Ryan J, Nguyen SG, Morgan KI, et al. DNA analyses of large pangolin scale seizures: species identification validation and case studies. Forensic Sci Int Anim Environ. 2021;1:100014. doi: 10.1016/j.fsiae.2021.100014

[34] Zhang H, Ades G, Miller MP, Yang F, Lai K, Fischer GA. Genetic identification of African pangolins and their origin in illegal trade. Global Ecol Conserv. 2020;23:e01119. doi: 10.1016/j.gecco.2020.e01119

[35] Din Dipita A, Missoup AD, Aguillon S, Lecompte E, Momboua BR, Chaber A-L, et al. Genetic tracing of the illegal trade of the white-bellied pangolin (*Phataginus tricuspis*) in western Central Africa. Sci Rep. 2024;14(1):13131. doi: 10.1038/s41598-024-63666-9 38849460 PMC11161582

[36] Gossé KJ, Gonedelé-Bi S, Dufour S, Danquah E, Gaubert P. Conservation genetics of the white-bellied pangolin in West Africa: a story of lineage admixture, declining demography, and wide sourcing by urban bushmeat markets. Ecol Evol. 2024;14(3):e11031. doi: 10.1002/ece3.11031 38435007 PMC10905243

[37] Zanvo S, Djagoun CAMS, Azihou AF, Djossa B, Afiademanyo K, Olayemi A, et al. Can DNA help trace the local trade of pangolins? Conservation genetics of white-bellied pangolins from the Dahomey Gap (West Africa). BMC Ecol Evol. 2022;22(1):16. doi: 10.1186/s12862-022-01971-5 35164675 PMC8842964

[38] Tinsman JC, Gruppi C, Bossu CM, Prigge T-L, Harrigan RJ, Zaunbrecher V, et al. Genomic analyses reveal poaching hotspots and illegal trade in pangolins from Africa to Asia. Science. 2023;382(6676):1282–6. doi: 10.1126/science.adi5066 38096373

[39] Hu J-Y, Hao Z-Q, Frantz L, Wu S-F, Chen W, Jiang Y-F, et al. Genomic consequences of population decline in critically endangered pangolins and their demographic histories. Natl Sci Rev. 2020;7(4):798–814. doi: 10.1093/nsr/nwaa031 34692098 PMC8288997

[40] Nash HC, Wirdateti, Low GW, Choo SW, Chong JL, Semiadi G, et al. Conservation genomics reveals possible illegal trade routes and admixture across pangolin lineages in Southeast Asia. Conserv Genet. 2018;19(5):1083–95. doi: 10.1007/s10592-018-1080-9

[41] Wang Q, Lan T, Li H, Sahu SK, Shi M, Zhu Y, et al. Whole-genome resequencing of Chinese pangolins reveals a population structure and provides insights into their conservation. Commun Biol. 2022;5(1):821. doi: 10.1038/s42003-022-03757-3 36008681 PMC9411537

[42] Wei S, Fan H, Zhou W, Huang G, Hua Y, Wu S, et al. Conservation genomics of the critically endangered Chinese pangolin. Sci China Life Sci. 2024;67(10):2051–61. doi: 10.1007/s11427-023-2540-y 38970727

[43] Buckingham E, Curry J, Emogor C, Tomsett L, Cooper N. Using natural history collections to investigate changes in pangolin (Pholidota: Manidae) geographic ranges through time. PeerJ. 2021;9:e10843. doi: 10.7717/peerj.10843 33614289 PMC7882139

[44] Battey CJ, Ralph PL, Kern AD. Predicting geographic location from genetic variation with deep neural networks. Elife. 2020;9:e54507. doi: 10.7554/eLife.54507 32511092 PMC7324158

[45] Portik DM, Smith LL, Bi K. An evaluation of transcriptome-based exon capture for frog phylogenomics across multiple scales of divergence (Class: Amphibia, Order: Anura). Mol Ecol Resour. 2016;16(5):1069–83. doi: 10.1111/1755-0998.12541 27241806

[46] Schott RK, Panesar B, Card DC, Preston M, Castoe TA, Chang BSW. Targeted capture of complete coding regions across divergent species. Genome Biol Evol. 2017;9(2):398–414. doi: 10.1093/gbe/evx005 28137744 PMC5381602

[47] Heinrich S, Wittman T, Ross J, Shepherd C, Challender D, Cassey P. The global trafficking of pangolins: A comprehensive summary of seizures and trafficking routes from 2010–2015. Selangor, Malaysia: TRAFFIC Southeast Asia; 2017.

[48] Allendorf FW, Hohenlohe PA, Luikart G. Genomics and the future of conservation genetics. Nat Rev Genet. 2010;11(10):697–709. doi: 10.1038/nrg2844 20847747

[49] Droissart V, Dauby G, Hardy OJ, Deblauwe V, Harris DJ, Janssens S, et al. Beyond trees: biogeographical regionalization of tropical Africa. J Biogeogr. 2018;45(5):1153–67. doi: 10.1111/jbi.13190

[50] Bernáthová I, Swiacká M, Bath Shéba Vitel LC, Tinsman JC, Hulva P, Černá Bolfíková B. Population structure and demographic history of two highly-trafficked species of pangolin in the Congo Basin. Sci Rep. 2024;14(1):22177. doi: 10.1038/s41598-024-68928-0 39333261 PMC11437027

[51] Lawson DJ, van Dorp L, Falush D. A tutorial on how not to over-interpret STRUCTURE and ADMIXTURE bar plots. Nat Commun. 2018;9(1):3258. doi: 10.1038/s41467-018-05257-7 30108219 PMC6092366

[52] Perez MF, Franco FF, Bombonato JR, Bonatelli IAS, Khan G, Romeiro‐Brito M, et al. Assessing population structure in the face of isolation by distance: are we neglecting the problem?. Divers Distrib. 2018;24(12):1883–9. doi: 10.1111/ddi.12816

[53] Luo S-J, Zhang Y, Johnson WE, Miao L, Martelli P, Antunes A, et al. Sympatric Asian felid phylogeography reveals a major Indochinese-Sundaic divergence. Mol Ecol. 2014;23(8):2072–92. doi: 10.1111/mec.12716 24629132

[54] Patel RP, Wutke S, Lenz D, Mukherjee S, Ramakrishnan U, Veron G, et al. Genetic structure and phylogeography of the leopard cat (*Prionailurus bengalensis*) inferred from mitochondrial genomes. J Hered. 2017;108(4):349–60. doi: 10.1093/jhered/esx017 28498987

[55] Li B, Li H, Shi M, Wang Q, Li H, Guo C, et al. Population genomics reveals deep diversification in Malayan pangolins. Mol Biol Evol. 2026;43(2):msag016. doi: 10.1093/molbev/msag016 41543495 PMC12888049

[56] Chan HK. Phylogeography and cryptic diversity of Occidozyga lima (Gravenhorst 1829). The University of Hong Kong; 2013.

[57] Banterng N, Ewart K, Sitam FT, Ogden R. Mitogenomic analysis of Thai *Sunda pangolins* reveals regional phylogeography and informs conservation management. Sci Rep. 2025;15(1):14067. doi: 10.1038/s41598-025-97182-1 40269012 PMC12018953

[58] Mason VC, Helgen KM, Murphy WJ. Comparative phylogeography of forest-dependent mammals reveals paleo-forest corridors throughout Sundaland. J Hered. 2019;110(2):158–72. doi: 10.1093/jhered/esy046 30247638

[59] Driller C, Merker S, Perwitasari-Farajallah D, Sinaga W, Anggraeni N, Zischler H. Stop and go – waves of tarsier dispersal mirror the genesis of Sulawesi island. PLoS One. 2015;10(11):e0141212. doi: 10.1371/journal.pone.0141212 26559527 PMC4641617

[60] Kunde MN, Barlow A, Klittich AM, Yakupova A, Patel RP, Fickel J, et al. First mitogenome phylogeny of the sun bear *Helarctos malayanus* reveals a deep split between Indochinese and Sundaic lineages. Ecol Evol. 2023;13(4):e9969. doi: 10.1002/ece3.9969 37082317 PMC10111171

[61] Wong PY-H, Chen Y, Prigge T-L, Zhang H, Rose-Jeffreys L, Ades G, et al. Seizure samples reveal complex evolutionary dynamics among Southeast Asian pangolins. Heredity (Edinb). 2026;:10.1038/s41437-026-00826–9. doi: 10.1038/s41437-026-00826-9 41703095 PMC13103069

[62] Holt BG, Lessard J-P, Borregaard MK, Fritz SA, Araújo MB, Dimitrov D. An update of Wallace’s zoogeographic regions of the world. Science. 2013;339(6115):74–8. doi: 10.1126/science.1228282 23258408

[63] Kreft H, Jetz W. A framework for delineating biogeographical regions based on species distributions. J Biogeogr. 2010;37(11):2029–53. doi: 10.1111/j.1365-2699.2010.02375.x

[64] Cai T, Fjeldså J, Wu Y, Shao S, Chen Y, Quan Q, et al. What makes the Sino‐Himalayan mountains the major diversity hotspots for pheasants?. J Biogeogr. 2017;45(3):640–51. doi: 10.1111/jbi.13156

[65] Koju NP, Zeng Z, Zhang G, Huang X, Yao Z, Wang X. Revalidation of *Manis aurita*, based on integrative morphological and genomic evidence. bioRxiv. 2025. https://www.biorxiv.org/content/10.1101/2025.07.05.663294v4 10.1038/s42003-026-10314-9 PMC13323723 42387092

[66] Lan T, Tian Y, Shi M, Liu B, Lin Y, Xia Y, et al. Enhancing inbreeding estimation and global conservation insights through chromosome-level assemblies of the Chinese and Malayan pangolin. Gigascience. 2025;14:giaf003. doi: 10.1093/gigascience/giaf003 39947250 PMC11825179

[67] Wiens BJ, Colella JP. That’s not a hybrid: how to distinguish patterns of admixture and isolation by distance. Mol Ecol Resour. 2025;25(3):e14039. doi: 10.1111/1755-0998.14039 39467042

[68] Marcer A, Haston E, Groom Q, Ariño AH, Chapman AD, Bakken T, et al. Quality issues in georeferencing: from physical collections to digital data repositories for ecological research. Divers Distrib. 2020;27(3):564–7. doi: 10.1111/ddi.13208

[69] Wilkie DS, Wieland M, Boulet H, Le Bel S, van Vliet N, Cornelis D, et al. Eating and conserving bushmeat in Africa. Afr J Ecol. 2016;54(4):402–14. doi: 10.1111/aje.12392

[70] Wasser SK, Wolock CJ, Kuhner MK, Brown JE 3rd, Morris C, Horwitz RJ, et al. Elephant genotypes reveal the size and connectivity of transnational ivory traffickers. Nat Hum Behav. 2022;6(3):371–82. doi: 10.1038/s41562-021-01267-6 35165434 PMC10693927

[71] Challender DWS, Embolo LE, Keboy Mov Linkey Iflankoy C, Mouafo ADT, Simo FT, Ullmann T, et al. Incentivizing pangolin conservation: decisions at CITES CoP19 may reduce conservation options for pangolins. Conservat Sci Prac. 2024;6(5). doi: 10.1111/csp2.13117

[72] Assembe-Mvondo S, Kan A. An overview of interactions between wildlife and forest illegalities in Cameroon. int forest rev. 2022;24(4):459–68. doi: 10.1505/146554822836282491

[73] Koutchoro AM, Amahowe OI, Houessou LG, Lougbegnon TO. Role of local markets in illegal wildlife trade and conservation efforts for trafficked species. Global Ecol Conserv. 2024;54:e03110. doi: 10.1016/j.gecco.2024.e03110

[74] Finch KN, Cronn RC, Ayala Richter MC, Blanc-Jolivet C, Correa Guerrero MC, De Stefano Beltrán L, et al. Predicting the geographic origin of Spanish Cedar (*Cedrela odorata* L.) based on DNA variation. Conserv Genet. 2020;21(4):625–39. doi: 10.1007/s10592-020-01282-6

[75] McNamara J, Kusimi JM, Rowcliffe JM, Cowlishaw G, Brenyah A, Milner-Gulland EJ. Long-term spatio-temporal changes in a West African bushmeat trade system. Conserv Biol. 2015;29(5):1446–57. doi: 10.1111/cobi.12545 26104770 PMC4745032

[76] Chng SC, Eaton JA, Krishnasamy K, Shepherd CR, Nijman V. In the market for extinction: an inventory of Jakarta’s bird markets. Traffic Report. 2015:31.

[77] Nijman V, Spaan D, Nekaris KA-I. Large-scale trade in legally protected marine mollusc shells from Java and Bali, Indonesia. PLoS One. 2015;10(12):e0140593. doi: 10.1371/journal.pone.0140593 26717021 PMC4696778

[78] Nijman V, Zhang MX, Shepherd CR. Pangolin trade in the Mong La wildlife market and the role of Myanmar in the smuggling of pangolins into China. Global Ecol Conserv. 2016;5:118–26. doi: 10.1016/j.gecco.2015.12.003

[79] Xi F, Chao X, Wu S, Zhang F. Curbing the trade in pangolin scales in China by revealing the characteristics of the illegal trade network. Sci Rep. 2025;15(1):2685. doi: 10.1038/s41598-025-87183-5 39838170 PMC11751142

[80] Omifolaji JK, Ikyaagba ET, Jimoh SO, Ibrahim AS, Ahmad S, Luan X. The emergence of Nigeria as a staging ground in the illegal pangolin exportation to South East Asia. Forensic Sci Int Reports. 2020;2:100138. doi: 10.1016/j.fsir.2020.100138

[81] Zhang M, Gouveia A, Qin T, Quan R, Nijman V. Illegal pangolin trade in northernmost Myanmar and its links to India and China. Global Ecol Conserv. 2017;10:23–31. doi: 10.1016/j.gecco.2017.01.006

[82] Aguillon S, Din Dipita A, Lecompte E, Missoup AD, Tindo M, Gaubert P. Development and characterization of 20 polymorphic microsatellite markers for the white-bellied pangolin *Phataginus tricuspis* (Mammalia, Pholidota). Mol Biol Rep. 2020;47(6):4827–33. doi: 10.1007/s11033-020-05511-6 32419053 PMC7230135

[83] Gaubert P, Zenatello M. Ancient DNA perspective on the failed introduction of mongooses in Italy during the XXth century. J Zool. 2009;279(3):262–9. doi: 10.1111/j.1469-7998.2009.00614.x

[84] Bennett EA, Massilani D, Lizzo G, Daligault J, Geigl EM, Grange T. Library construction for ancient genomics: single strand or double strand?. BioTechn. 2014;56(6):289–300. doi: 10.2144/000114176 24924389

[85] Li H, Durbin R. Fast and accurate short read alignment with Burrows-Wheeler transform. Bioinformatics. 2009;25(14):1754–60. doi: 10.1093/bioinformatics/btp324 19451168 PMC2705234

[86] Hari R, Siew Woh C. Complete mitogenome of *Manis javanica* and *Manis pentadactyla*; 2015 [cited 2025 July 1]. GenBank accession: KT445979. Available from: https://www.ncbi.nlm.nih.gov/nuccore/KT445979.1

[87] Hassanin A, Hugot J-P, van Vuuren BJ. Comparison of mitochondrial genome sequences of pangolins (Mammalia, Pholidota). C R Biol. 2015;338(4):260–5. doi: 10.1016/j.crvi.2015.02.003 25746396

[88] Peltzer A, Jäger G, Herbig A, Seitz A, Kniep C, Krause J, et al. EAGER: efficient ancient genome reconstruction. Genome Biol. 2016;17:60. doi: 10.1186/s13059-016-0918-z 27036623 PMC4815194

[89] Ewels PA, Peltzer A, Fillinger S, Patel H, Alneberg J, Wilm A, et al. The nf-core framework for community-curated bioinformatics pipelines. Nat Biotechnol. 2020;38(3):276–8. doi: 10.1038/s41587-020-0439-x 32055031

[90] von Takach B, Ranjard L, Burridge CP, Cameron SF, Cremona T, Eldridge MDB, et al. Population genomics of a predatory mammal reveals patterns of decline and impacts of exposure to toxic toads. Mol Ecol. 2022;31(21):5468–86. doi: 10.1111/mec.16680 36056907 PMC9826391

[91] Chen S, Zhou Y, Chen Y, Gu J. fastp: an ultra-fast all-in-one FASTQ preprocessor. Bioinformatics. 2018;34(17):i884–90. doi: 10.1093/bioinformatics/bty560 30423086 PMC6129281

[92] Daley T, Smith AD. Predicting the molecular complexity of sequencing libraries. Nat Methods. 2013;10(4):325–7. doi: 10.1038/nmeth.2375 23435259 PMC3612374

[93] Neukamm J, Peltzer A, Nieselt K. DamageProfiler: fast damage pattern calculation for ancient DNA. Bioinformatics. 2021;37(20):3652–3. doi: 10.1093/bioinformatics/btab190 33890614

[94] Ginolhac A, Rasmussen M, Gilbert MTP, Willerslev E, Orlando L. mapDamage: testing for damage patterns in ancient DNA sequences. Bioinformatics. 2011;27(15):2153–5. doi: 10.1093/bioinformatics/btr347 21659319

[95] Okonechnikov K, Conesa A, García-Alcalde F. Qualimap 2: advanced multi-sample quality control for high-throughput sequencing data. Bioinformatics. 2016;32(2):292–4. doi: 10.1093/bioinformatics/btv566 26428292 PMC4708105

[96] Ewels P, Magnusson M, Lundin S, Käller M. MultiQC: summarize analysis results for multiple tools and samples in a single report. Bioinformatics. 2016;32(19):3047–8. doi: 10.1093/bioinformatics/btw354 27312411 PMC5039924

[97] Choo SW, Rayko M, Tan TK, Hari R, Komissarov A, Wee WY, et al. Pangolin genomes and the evolution of mammalian scales and immunity. Genome Res. 2016;26(10):1312–22. doi: 10.1101/gr.203521.115 27510566 PMC5052048

[98] Manichaikul A, Mychaleckyj JC, Rich SS, Daly K, Sale M, Chen W-M. Robust relationship inference in genome-wide association studies. Bioinformatics. 2010;26(22):2867–73. doi: 10.1093/bioinformatics/btq559 20926424 PMC3025716

[99] Danecek P, Auton A, Abecasis G, Albers CA, Banks E, DePristo MA, et al. The variant call format and VCFtools. Bioinformatics. 2011;27(15):2156–8. doi: 10.1093/bioinformatics/btr330 21653522 PMC3137218

[100] Wickham H. ggplot2. WIREs Comput Stat. 2011;3(2):180–5. doi: 10.1002/wics.147

[101] Chang CC, Chow CC, Tellier LC, Vattikuti S, Purcell SM, Lee JJ. Second-generation PLINK: rising to the challenge of larger and richer datasets. Gigascience. 2015;4:7. doi: 10.1186/s13742-015-0047-8 25722852 PMC4342193

[102] Alexander DH, Novembre J, Lange K. Fast model-based estimation of ancestry in unrelated individuals. Genome Res. 2009;19(9):1655–64. doi: 10.1101/gr.094052.109 19648217 PMC2752134

[103] Kopelman NM, Mayzel J, Jakobsson M, Rosenberg NA, Mayrose I. Clumpak: a program for identifying clustering modes and packaging population structure inferences across K. Mol Ecol Resour. 2015;15(5):1179–91. doi: 10.1111/1755-0998.12387 25684545 PMC4534335

[104] Sayre R, Dangermond J, Frye C, Vaughan R, Aniello P, Breyer S. A new map of global ecological land units—an ecophysiographic stratification approach. Washington, DC: Association of American Geographers; 2014.

[105] Jombart T, Ahmed I. adegenet 1.3-1: new tools for the analysis of genome-wide SNP data. Bioinformatics. 2011;27(21):3070–1. doi: 10.1093/bioinformatics/btr521 21926124 PMC3198581

[106] Vavrek MJ. Fossil: palaeoecological and palaeogeographical analysis tools. Palaeontol Electron. 2011;14(1):16.

[107] Wiens BJ, DeCicco LH, Colella JP. triangulaR: an R package for identifying AIMs and building triangle plots using SNP data from hybrid zones. Heredity (Edinb). 2025;134(5):251–62. doi: 10.1038/s41437-025-00760-2 40216927 PMC12056084

[108] Risk C, James PMA. Optimal cross‐validation strategies for selection of spatial interpolation models for the Canadian forest fire weather index system. Earth Space Sci. 2022;9(2). doi: 10.1029/2021ea002019

[109] Hijmans RJ, Williams E, Vennes C, Hijmans MRJ. Package ‘geosphere’. Spherical Trigonometry. 2017;1(7):1–45.

