# Supplementary figures and images for: Targeted sequencing enhances detection of pangolin trafficking hotspots and dynamics of both domestic and global trade markets
Source: PLoS Biol. 2026 May 7;24(5):e3003762. doi: 10.1371/journal.pbio.3003762 (PMC13152146; doi:10.1371/journal.pbio.3003762)

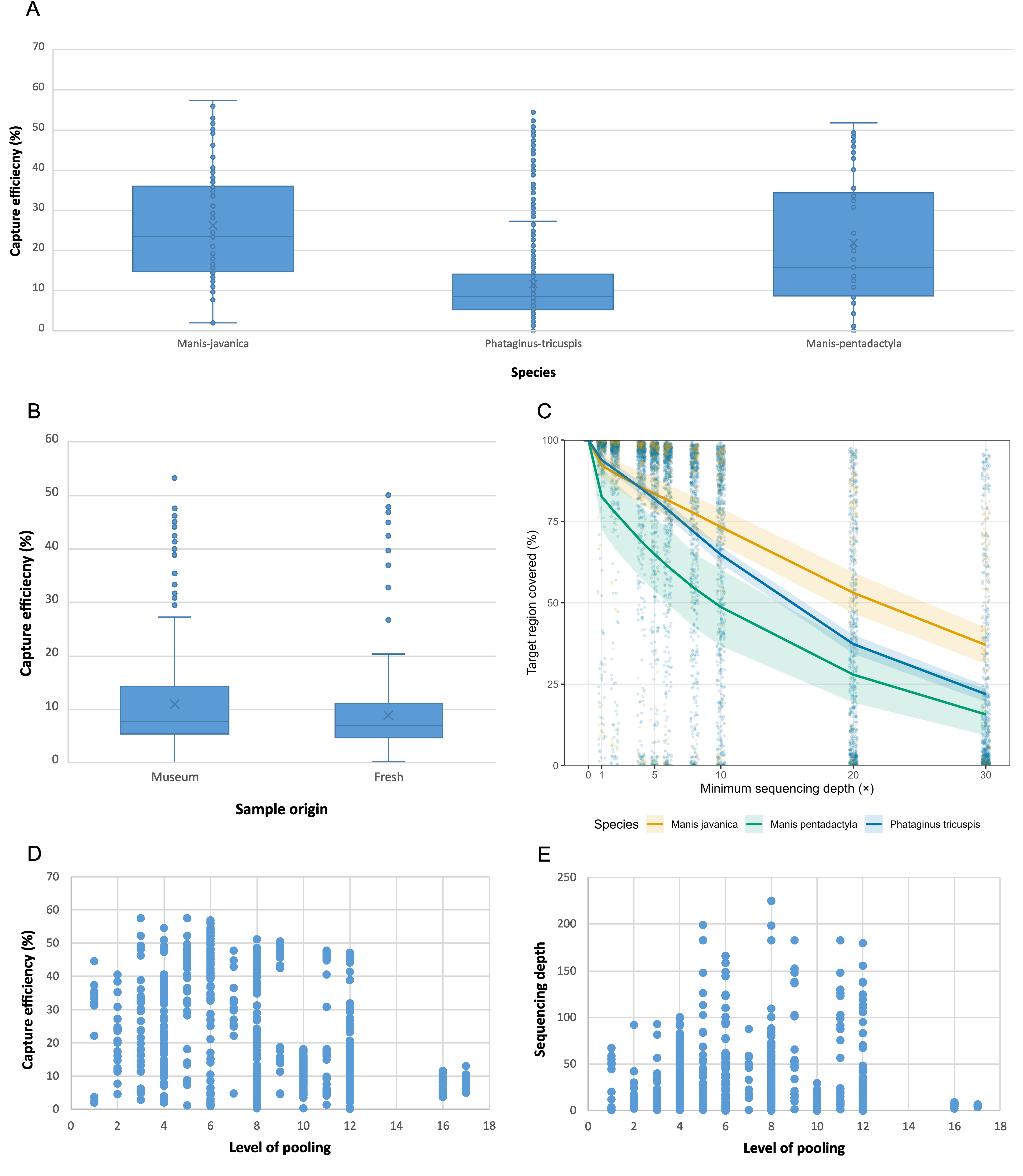

Supplement: S1 Fig — Box and whisker plots of capture efficiency across (A) species and (B) quality (museum vs. fresh) with X representing the mean and outliers shown as points. (C) A scatter-line plot of the proportion of gene-capture target region covered at various depths of coverage for each species (lines indicate the mean and shaded areas indicate 95% confidence intervals). Scatter plots comparing the number of individuals pooled in a single capture reaction to (D) capture efficiency and (E) sequencing depth. The underlying numerical data are provided in S1 Data. (TIF) [file pbio.3003762.s001.tif]

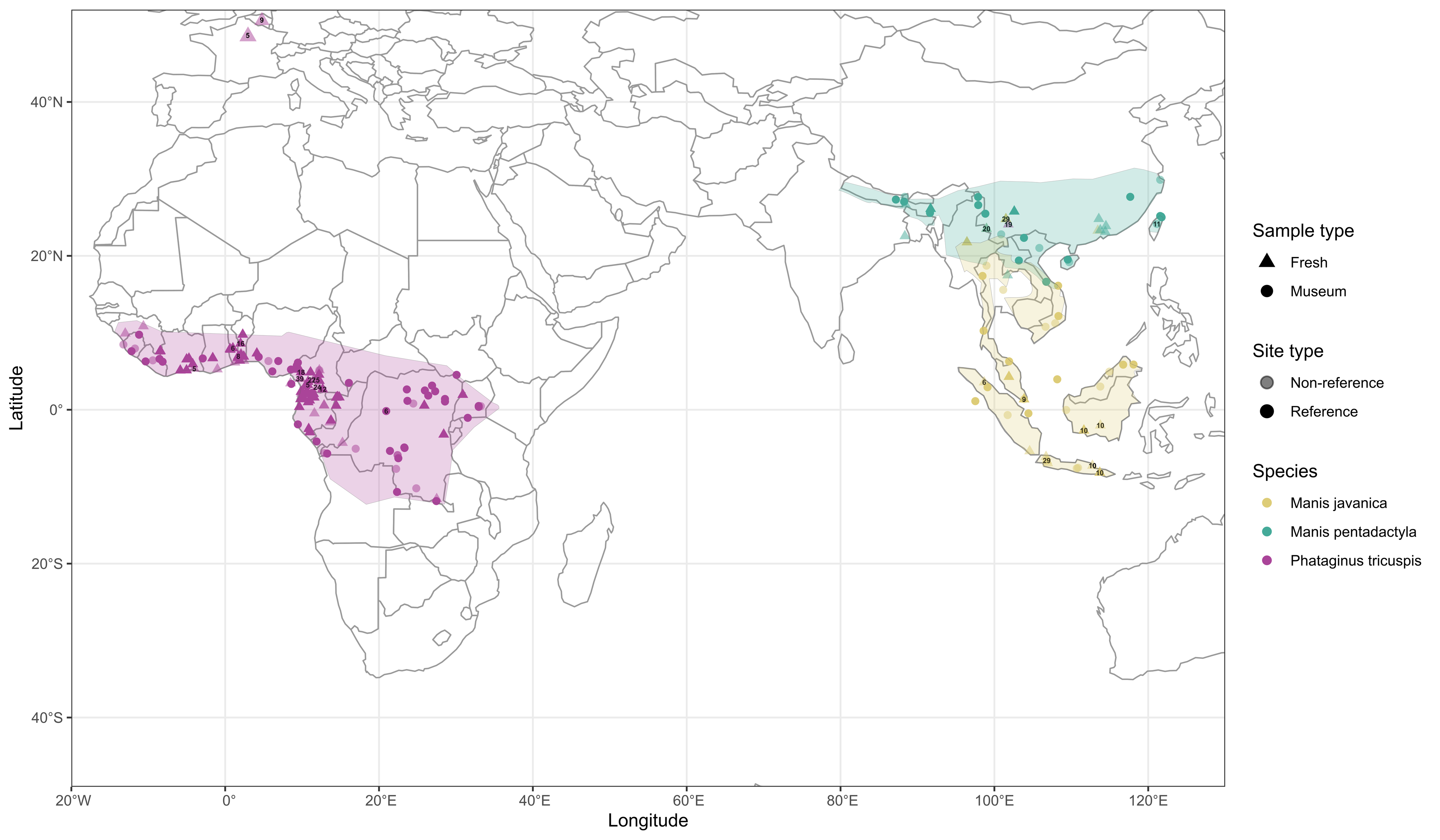

Supplement: S2 Fig — Sampling locations of both samples from this study and previously published studies for all three species. Reference samples are field-site and restricted-range market samples used as the reference set for trade tracing (known localities). Non-reference samples are those from unknown localities, including long-range markets, international trade seizures, and samples with missing sampling locality information. These samples represent individuals that were gene-captured, sequenced, and SNP-called, and do not necessarily correspond to the subsets used in downstream analyses, which applied method-specific filtering thresholds. Base layers include country data from Natural Earth (http://www.naturalearthdata.com) and the modeled species’ range from occurrence data previously collated (https://data.nhm.ac.uk/dataset/natalie-cooper; [43]). See S1 Appendix for detailed information on each sample (and underlying numerical data) and S1 Text for a breakdown of different site types. (TIF) [file pbio.3003762.s002.tif]

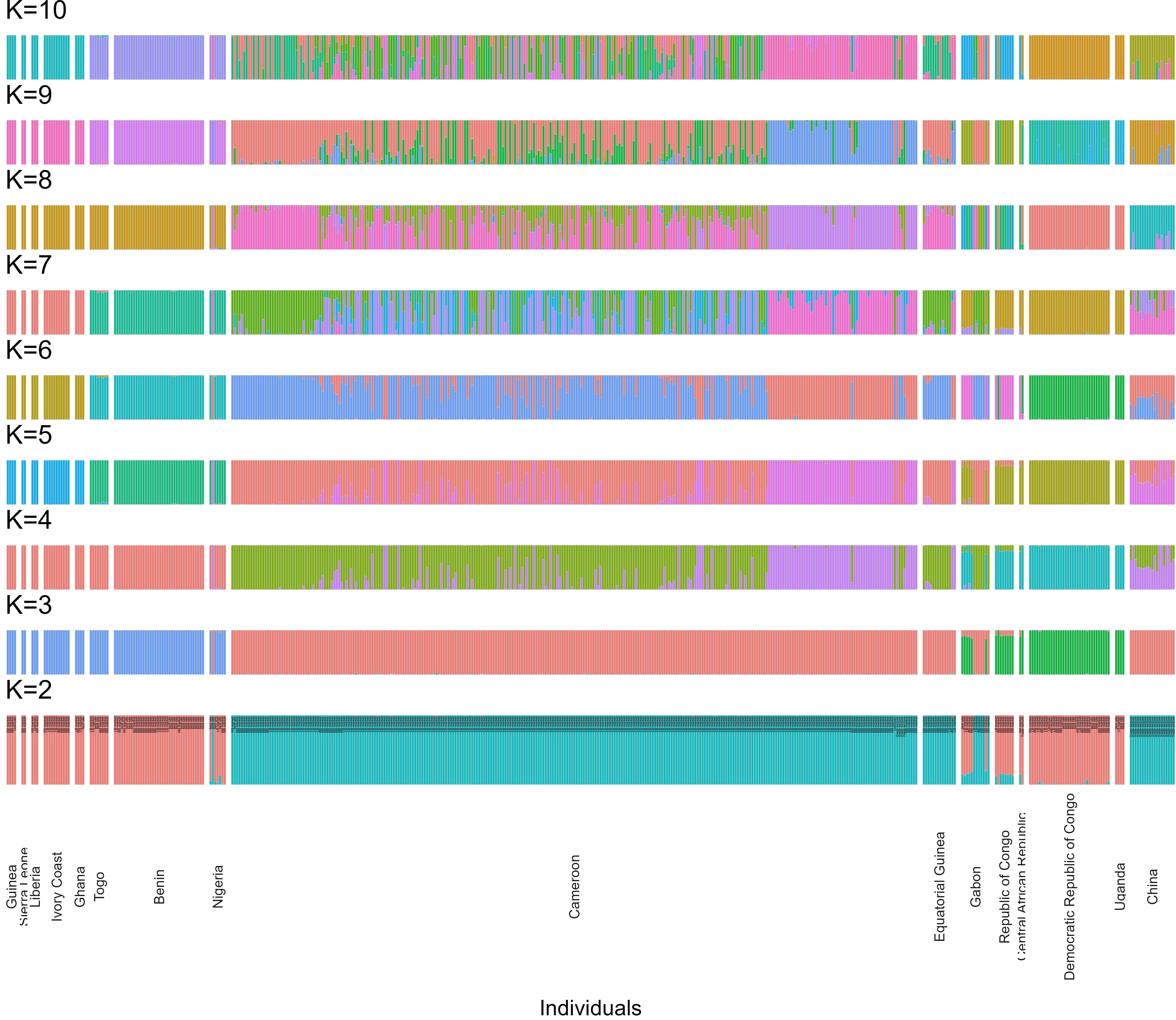

Supplement: S3 Fig — Samples belonging to countries are clumped, and each site type is labeled (8,312 SNPs, 456 individuals). Samples are ordered from the South-East to the North-West in each country. The underlying numerical data are provided in S1 Data. (TIF) [file pbio.3003762.s003.tif]

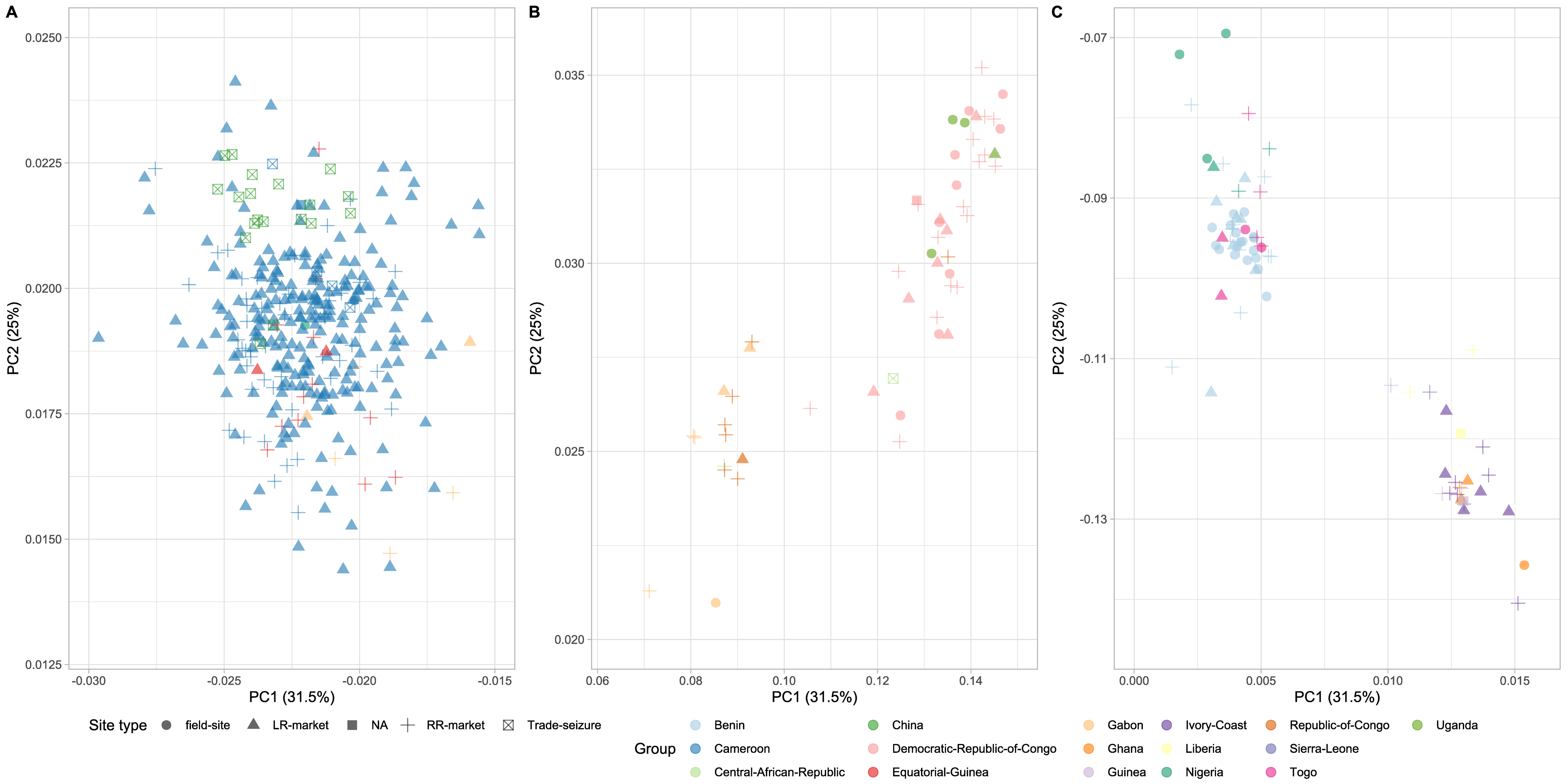

Supplement: S4 Fig — Points are colored by country and shaped by type of site from which the sample was collected (8,520 SNPs, 427 individuals). These represent the three major clades of the species (A) central-western Africa, (B) central Africa, and (C) western Africa. The underlying numerical data are provided in S1 Data. (TIF) [file pbio.3003762.s004.tif]

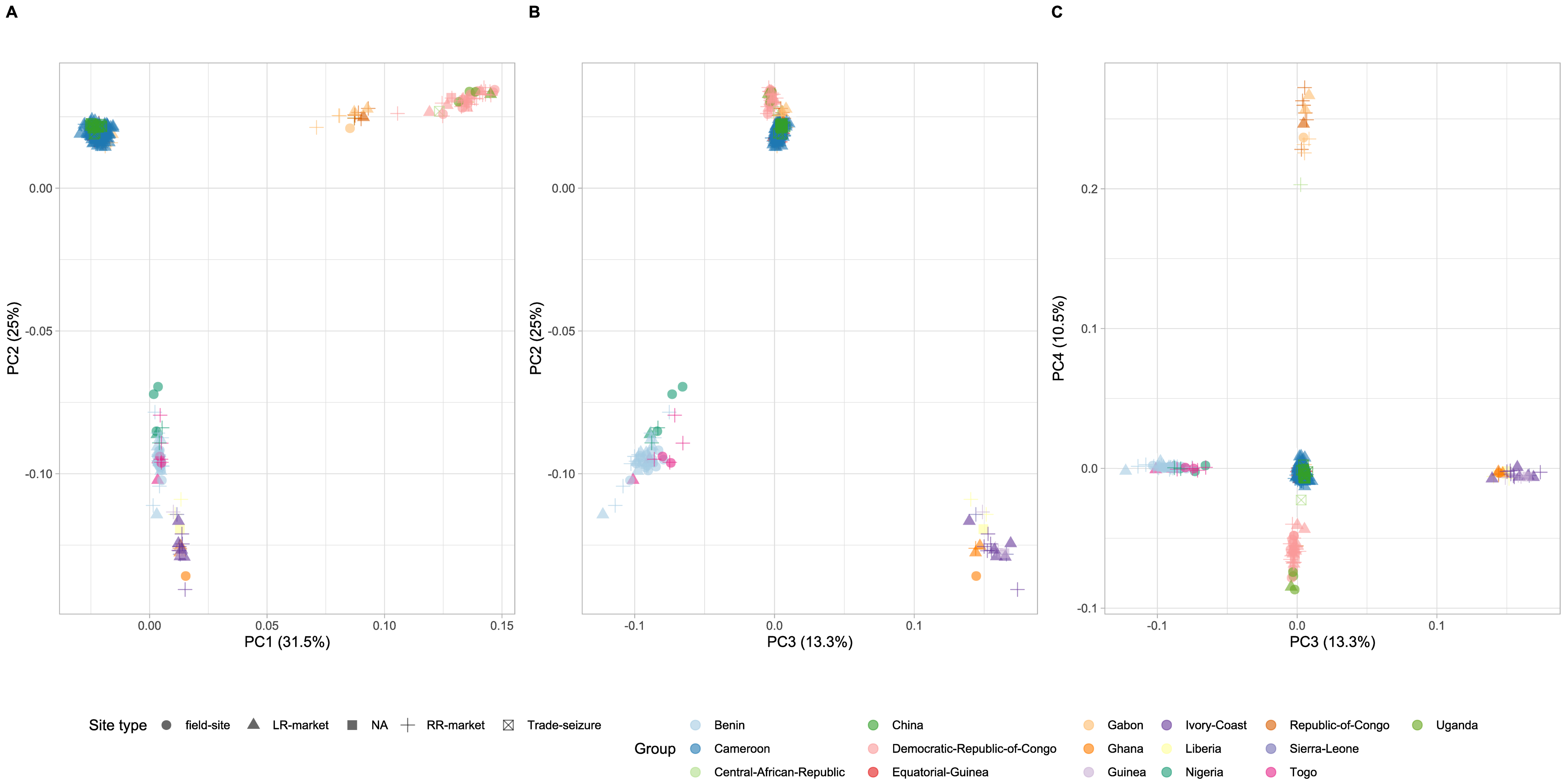

Supplement: S5 Fig — Points are colored by country and shaped by type of site from which the sample was collected (8,520 SNPs, 427 individuals). The underlying numerical data are provided in S1 Data. (TIF) [file pbio.3003762.s005.tif]

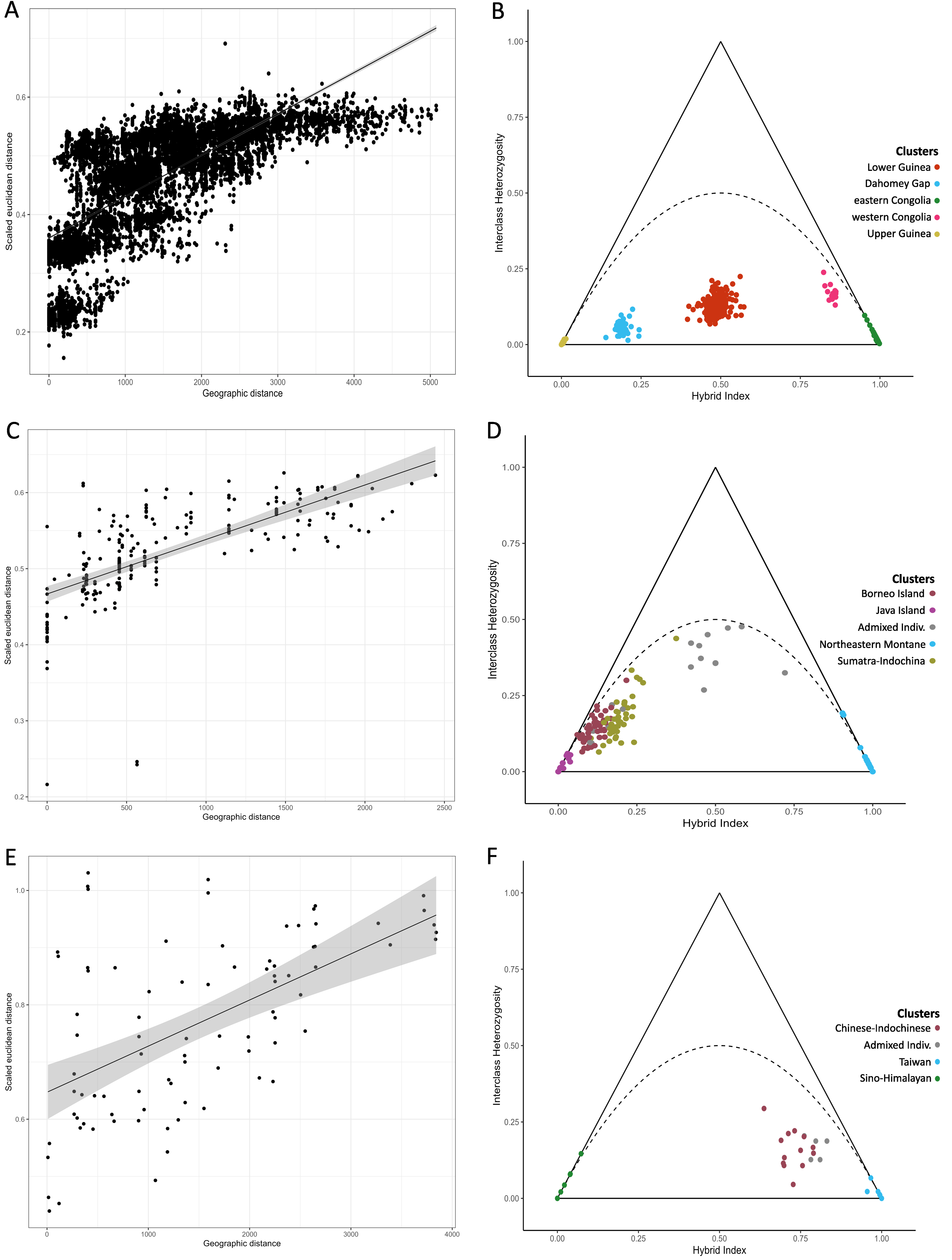

Supplement: S6 Fig — Isolation-by-distance (IBD) plots comparing pairwise genetic divergence (Euclidean distance) with pairwise geographic distance (km) across all individuals from each of the three pangolin species: (A) white-bellied (Phataginus tricuspis), (C) Sunda (Manis javanica), and (E) Chinese (Manis pentadactyla) pangolins. Triangle plots used for discriminating IBD-like clines from genuine admixture across the species’ range for (B) white-bellied (Phataginus tricuspis), (D) Sunda (Manis javanica), and (F) Chinese (Manis pentadactyla) pangolins. Reference populations represent the extremes of each species’ range. The underlying numerical data are provided in S1 Data. (TIF) [file pbio.3003762.s006.tif]

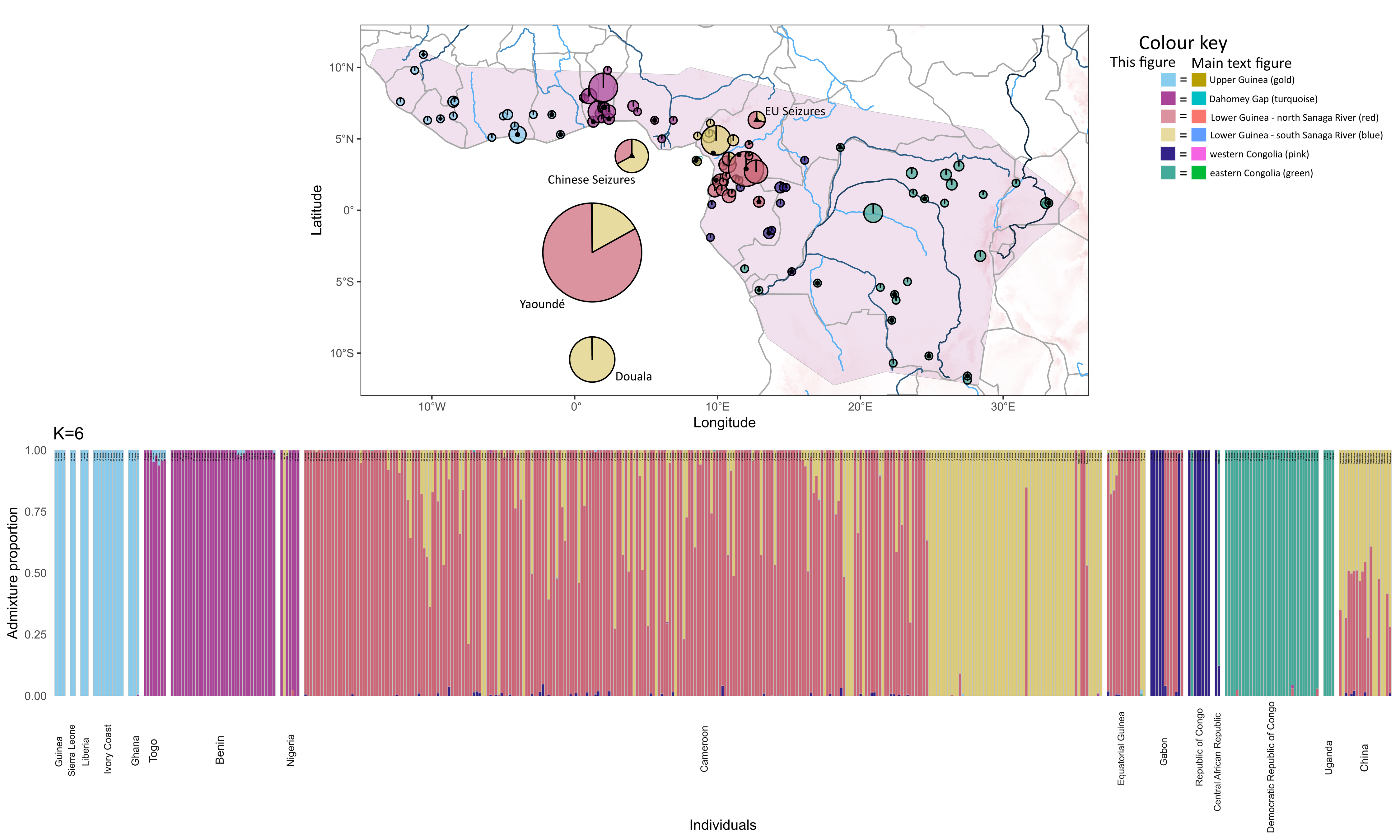

Supplement: S7 Fig — Samples belonging to countries are clumped, and each site type is labeled (8,312 SNPs, 456 individuals). Samples are ordered from the South-East to the North-West in each country. A color key is provided to distinguish between the colors and populations indicated in the main text and this figure. Base layers include country and river data from Natural Earth (http://www.naturalearthdata.com; darker blue indicates larger rivers), elevation from GlobalSolarAtlas-v2 (https://globalsolaratlas.info, in deep red), and the modeled species’ range from occurrence data previously collated (https://data.nhm.ac.uk/dataset/natalie-cooper; [43]). The underlying numerical data are provided in S1 Data. (TIF) [file pbio.3003762.s007.tif]

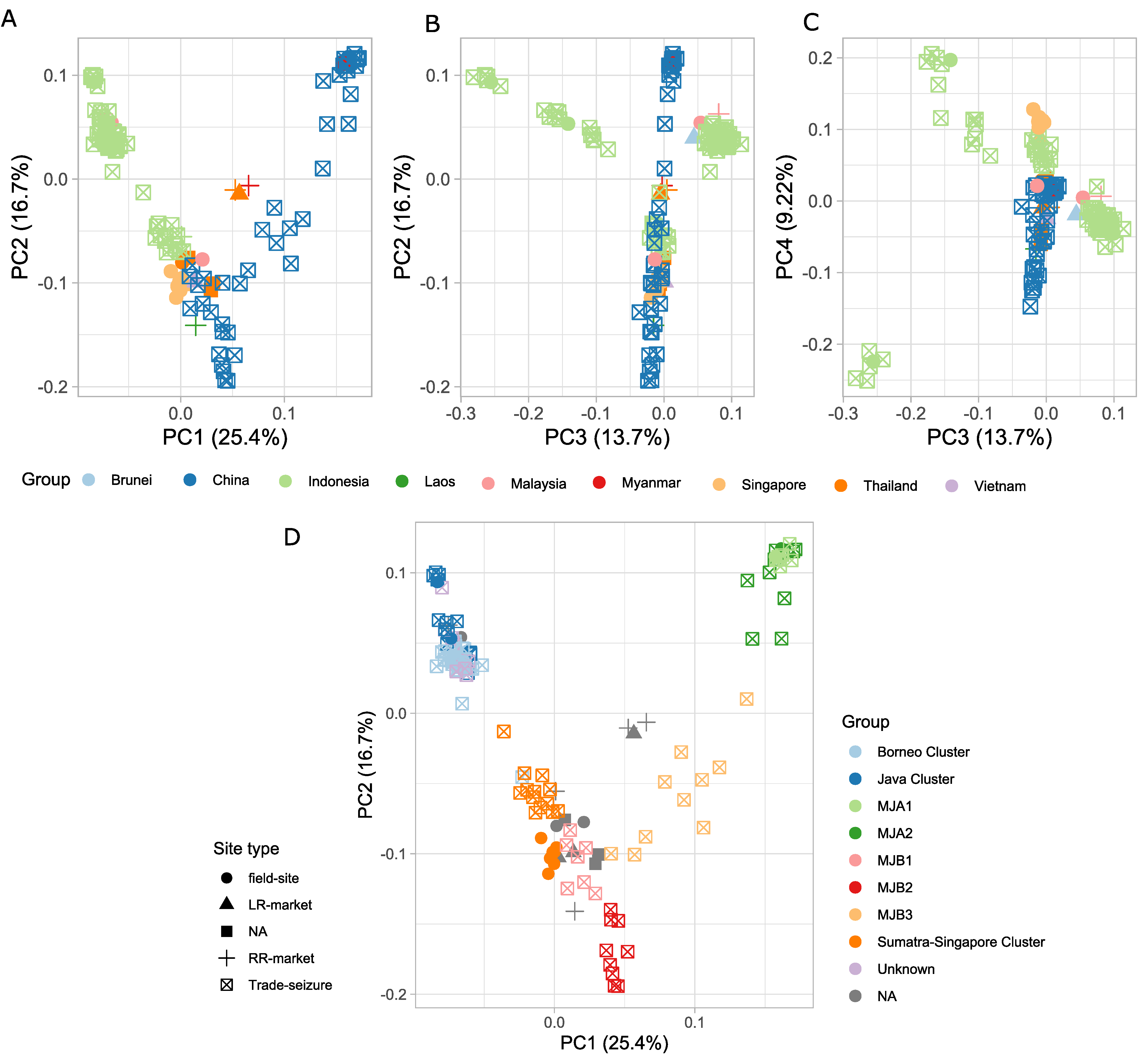

Supplement: S8 Fig — This is from 4,418 SNPs and 149 individuals for the (A–C) top four axes of differentiation. Points are colored by country and shaped by type of site from which the sample was collected. (D) PCA with the top two axes of differentiation in which points are colored by previously identified clusters [39,40] and shaped by type of site from which the sample was collected (NA represent samples that come from this study and thus did not yet have a cluster). The underlying numerical data are provided in S1 Data. (TIF) [file pbio.3003762.s008.tif]

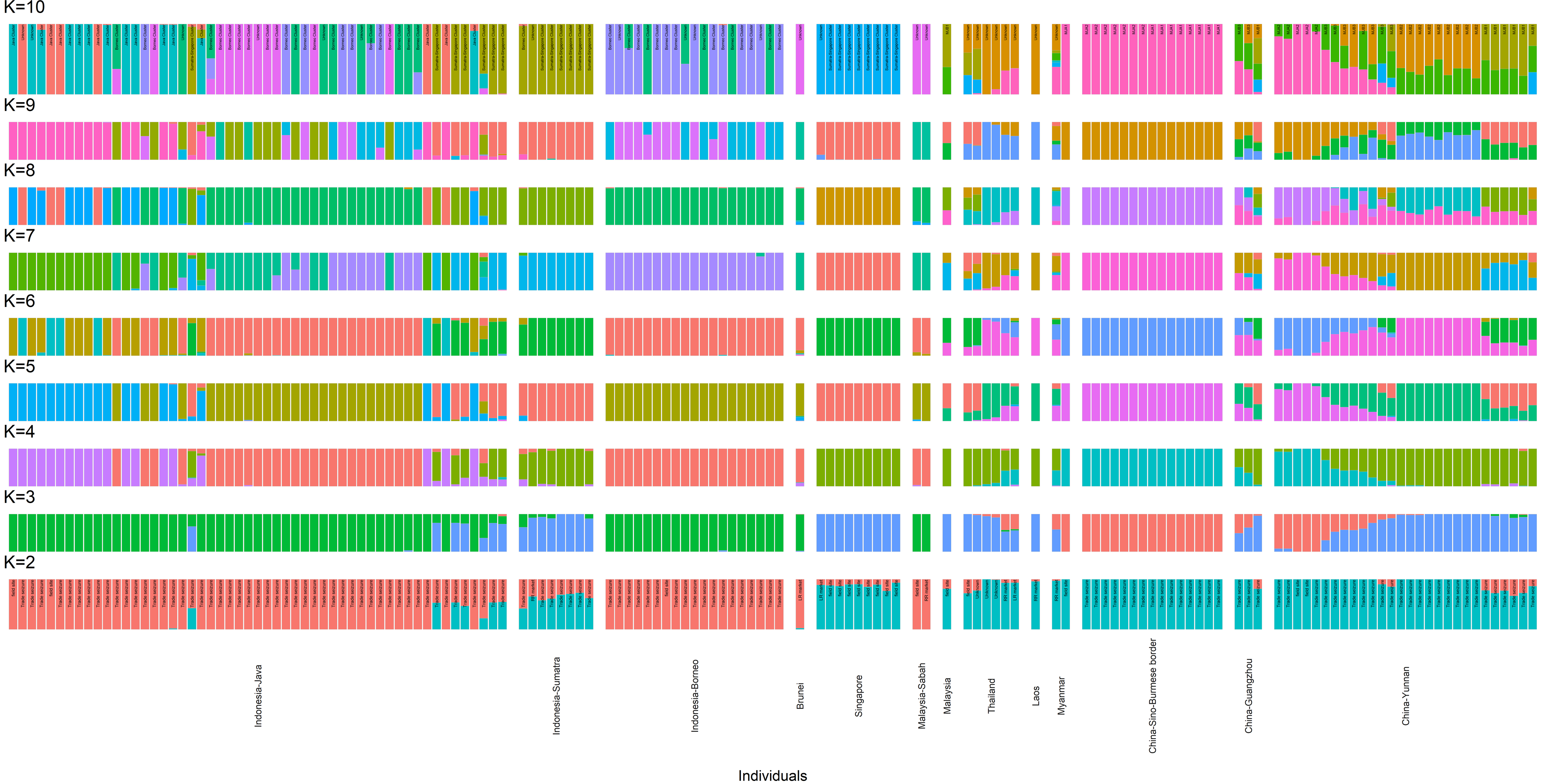

Supplement: S9 Fig — Samples belonging to countries are clumped, and each site type is labeled (4,162 SNPs, 148 individuals). Samples are ordered from South-East to North-West in each country. The underlying numerical data are provided in S1 Data. (TIF) [file pbio.3003762.s009.tif]

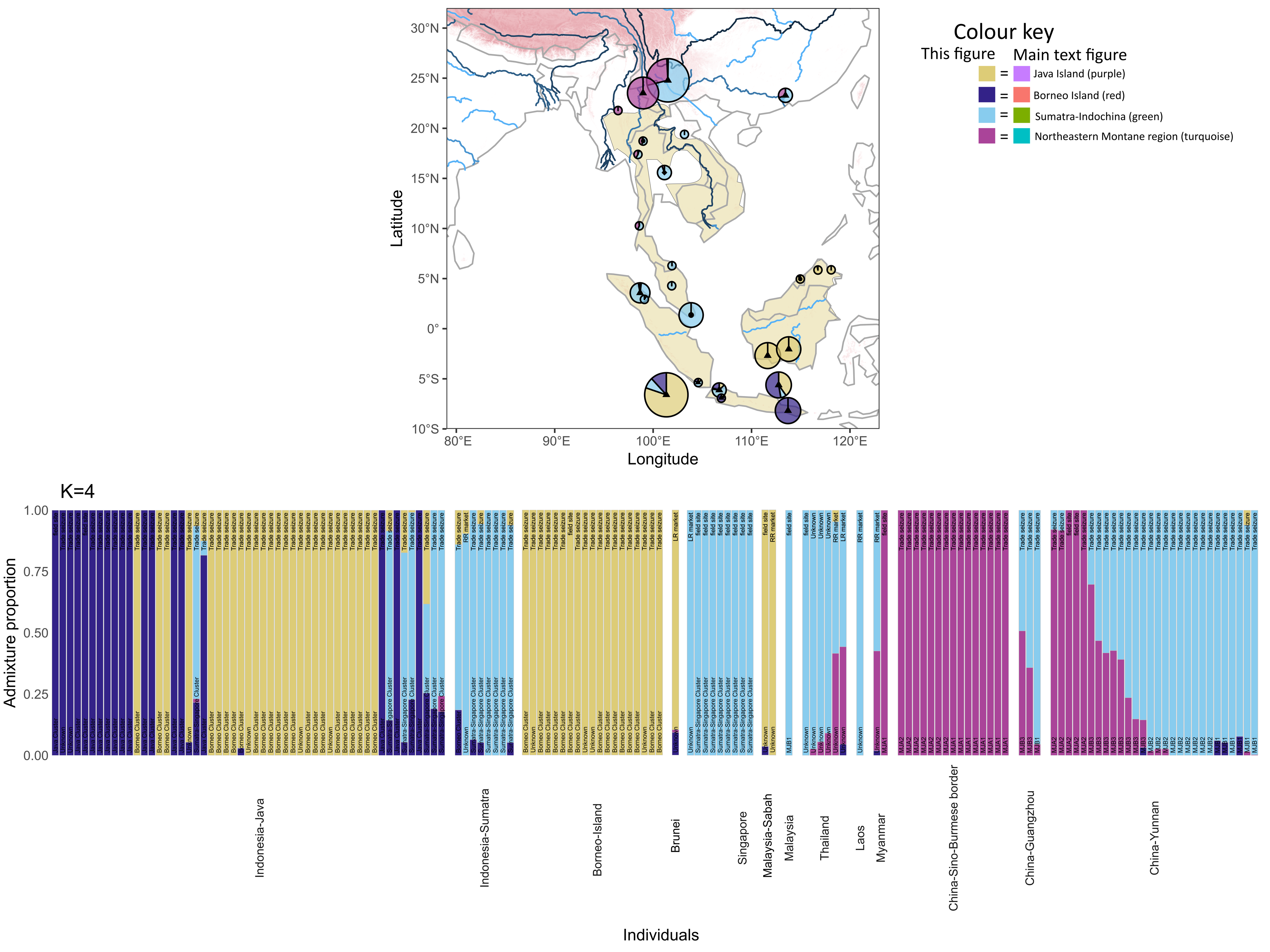

Supplement: S10 Fig — Samples belonging to countries are clumped, and each site type is labeled (4,162 SNPs, 148 individuals). Samples are ordered from South-East to North-West in each country. A color key is provided to distinguish between the colors and populations indicated in the main text and this figure. Base layers include country and river data from Natural Earth (http://www.naturalearthdata.com; darker blue indicates larger rivers), elevation from GlobalSolarAtlas-v2 (https://globalsolaratlas.info, in deep red) and the modeled species’ range from occurrence data previously collated (https://data.nhm.ac.uk/dataset/natalie-cooper; [43]). The underlying numerical data are provided in S1 Data. (TIF) [file pbio.3003762.s010.tif]

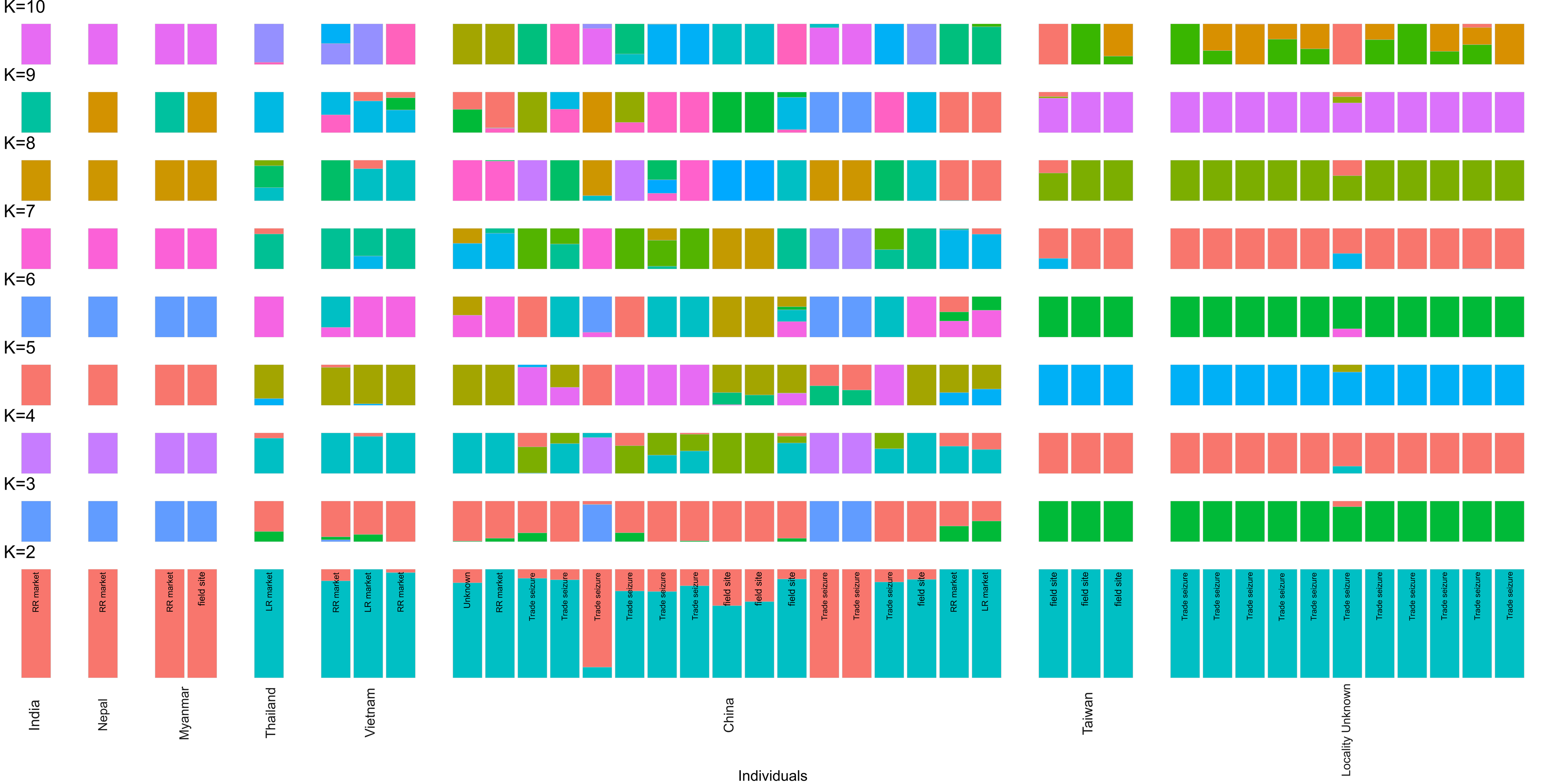

Supplement: S11 Fig — Samples belonging to countries are clumped, and each site type is labeled (816 SNPs, 39 individuals). Samples are ordered from South-East to North-West in each country. The underlying numerical data are provided in S1 Data. (TIF) [file pbio.3003762.s011.tif]

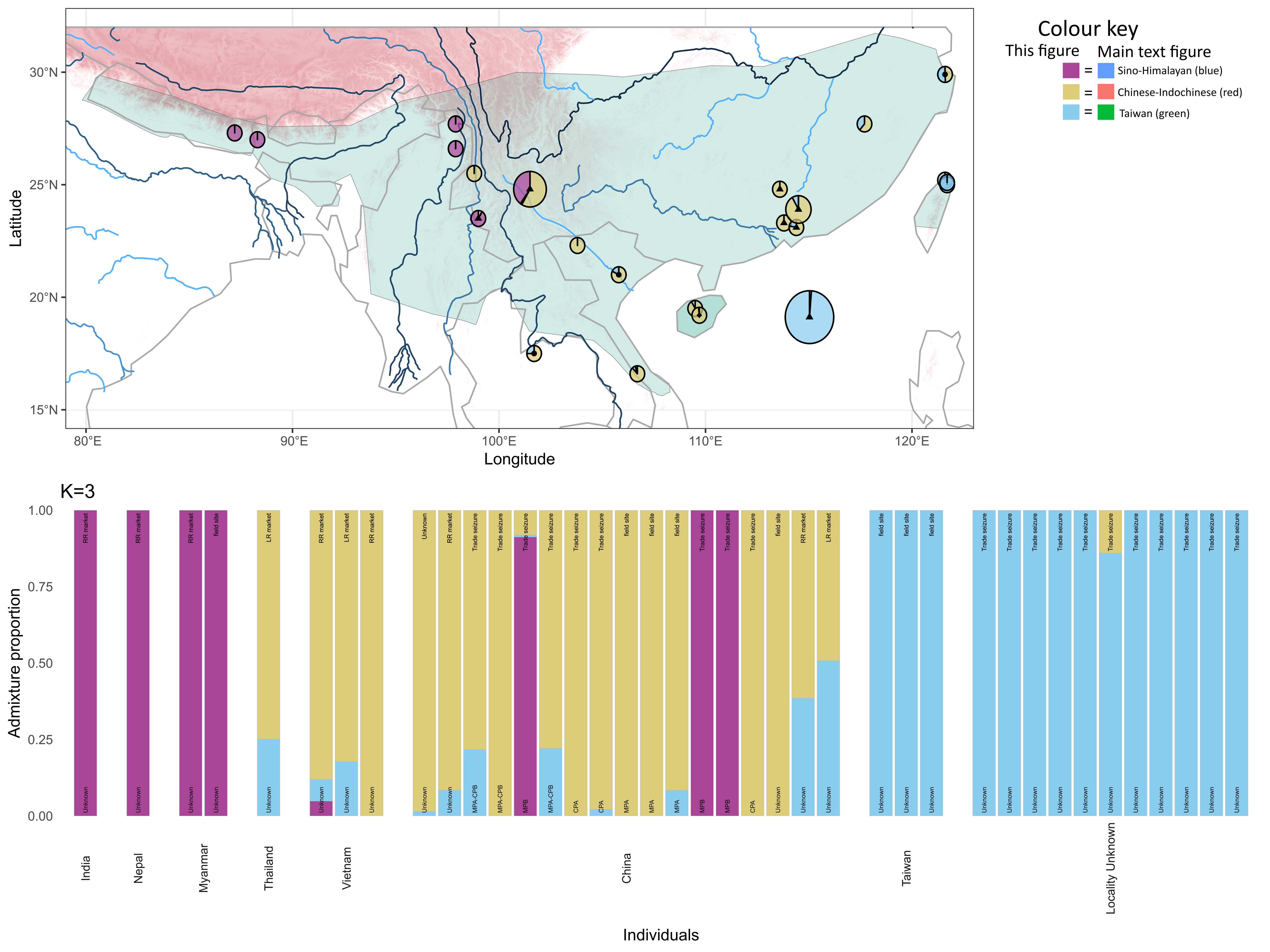

Supplement: S12 Fig — Samples belonging to countries are clumped, and each site type is labeled (816 SNPs, 39 individuals). Samples are ordered from South-East to North-West in each country. A color key is provided to distinguish between the colors and populations indicated in the main text and this figure. Base layers include country and river data from Natural Earth (http://www.naturalearthdata.com; darker blue indicates larger rivers), elevation from GlobalSolarAtlas-v2 (https://globalsolaratlas.info, in deep red) and the modeled species’ range from occurrence data previously collated (https://data.nhm.ac.uk/dataset/natalie-cooper; [43]). The underlying numerical data are provided in S1 Data. (TIF) [file pbio.3003762.s012.tif]

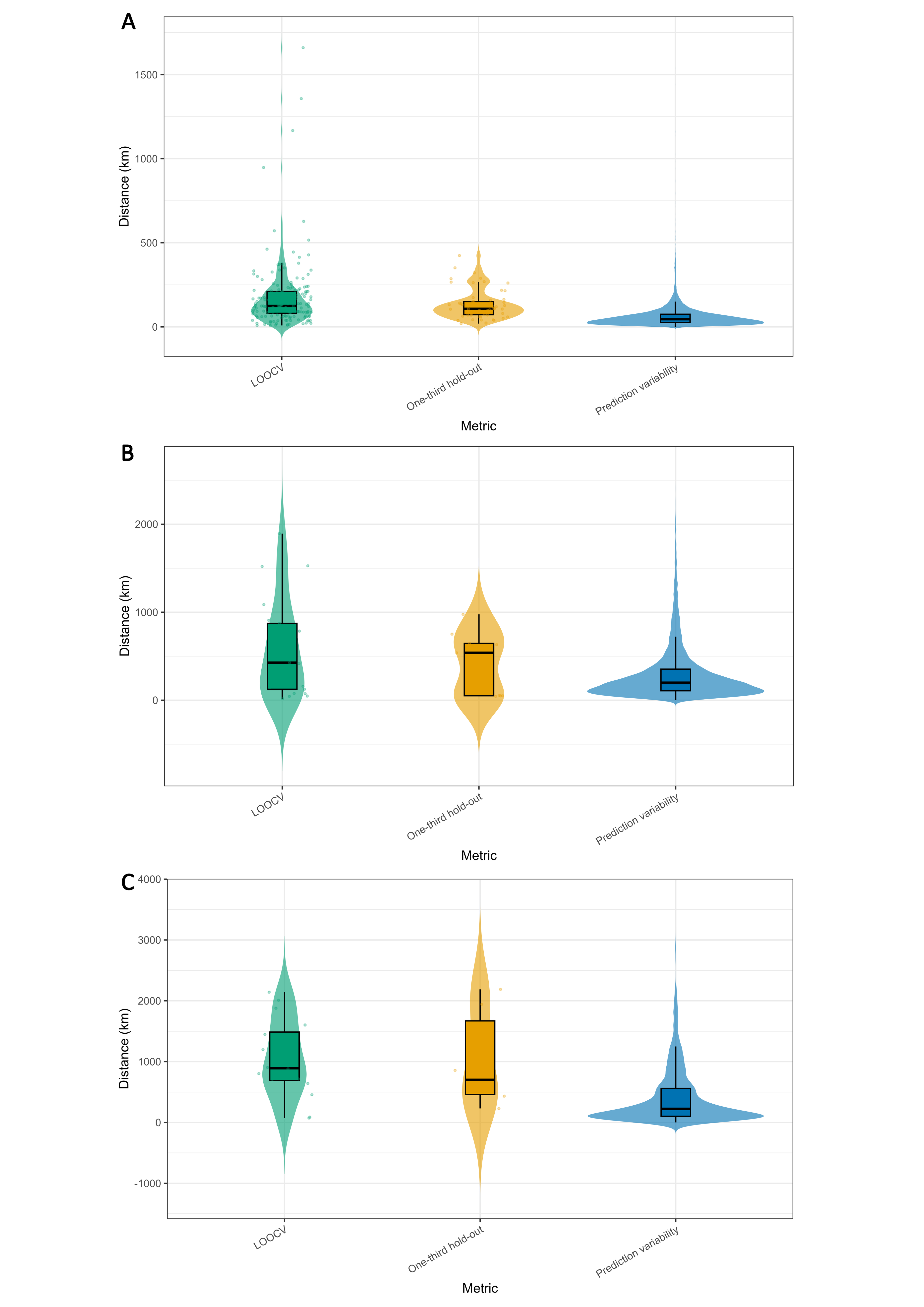

Supplement: S13 Fig — (A) White-bellied (Phataginus tricuspis), (B) Sunda (Manis javanica), and (C) Chinese (Manis pentadactyla) pangolins. Violin plots show the distribution of prediction distances (km) across three metrics: leave-one-out cross-validation (LOOCV) predictive error, one-third hold-out predictive error, and prediction variability (bootstrap centroid-based dispersion). The median is indicated by the central line, and the interquartile range (IQR) by the boxplot. Individual points represent per-sample distances (shown for hold-out and LOOCV only due to the large sample size for prediction variability across the 100 bootstraps). The underlying numerical data are provided in S1 Data. (TIF) [file pbio.3003762.s013.tif]

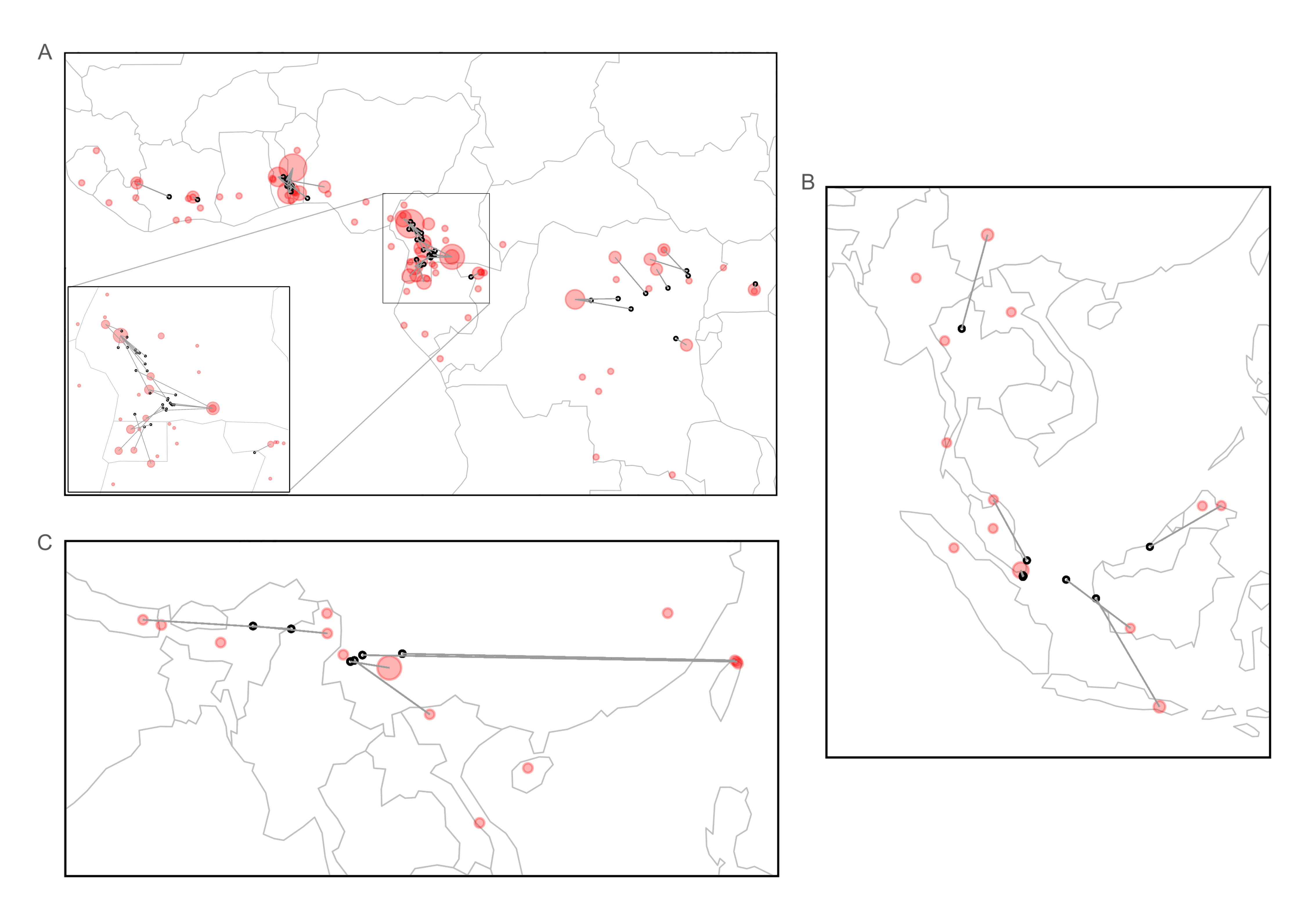

Supplement: S14 Fig — (A) White-bellied (Phataginus tricuspis), (B) Sunda (Manis javanica), and (C) Chinese (Manis pentadactyla) pangolins. Black circles indicate the most likely centroidal origin across 100 bootstrap replicates of each sample, with the gray line connecting it to its true origin location (where it was collected). Training sample locations (field collected) are indicated by red circles (for each case, the larger the circle size, the more training samples). See S1 Table for more details. The base layer country data is from Natural Earth (http://www.naturalearthdata.com). The underlying numerical data are provided in S1 Data. (TIF) [file pbio.3003762.s014.tif]

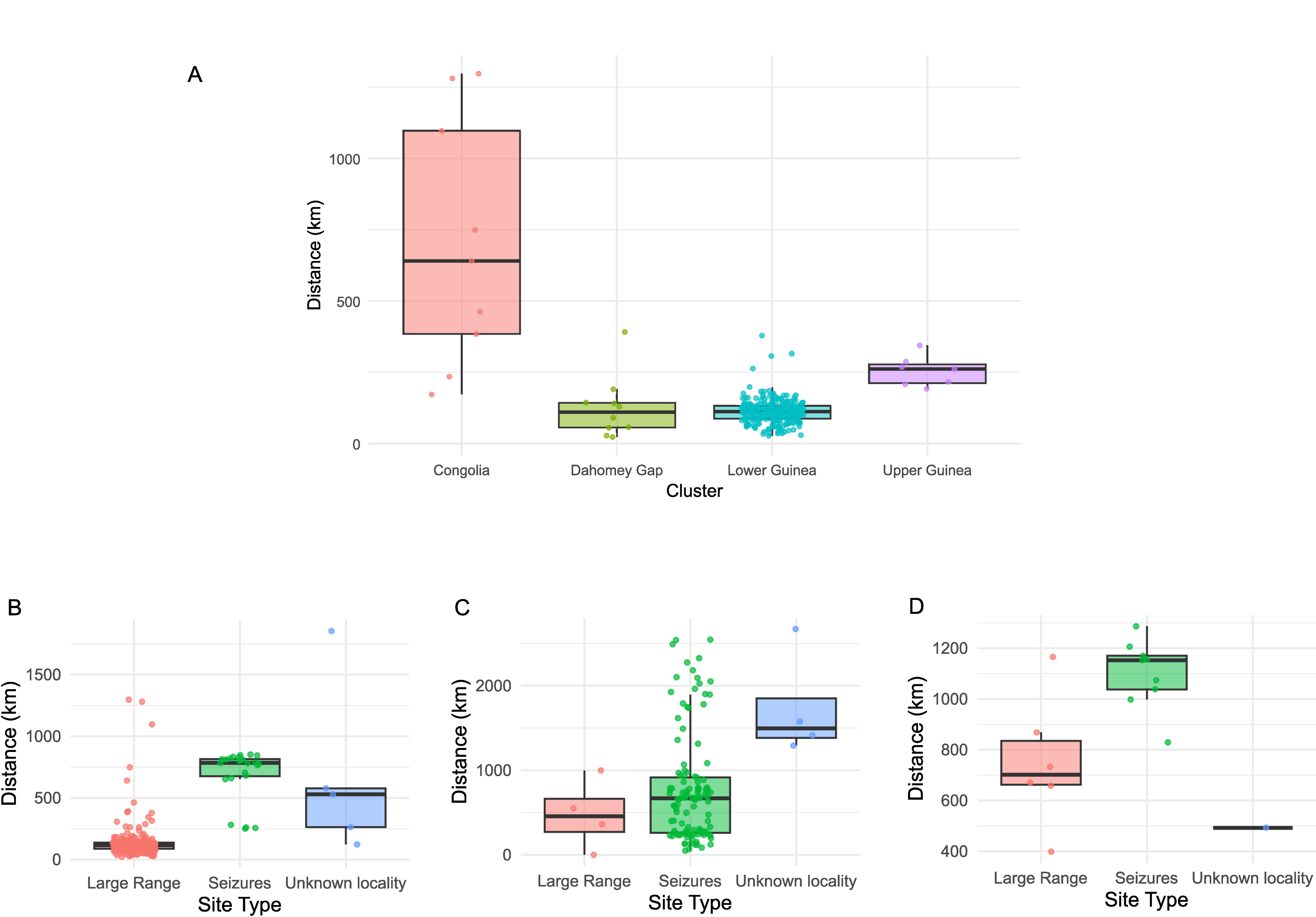

Supplement: S15 Fig — (A) White-bellied pangolin (Phataginus tricuspis) by cluster. (B) White-bellied pangolin (P. tricuspis) different site-types (seizures are not an accurate representation as these actually come from Europe and Asia). (C) Sunda pangolin (Manis javanica) different site-types. (D) Chinese pangolin (Manis pentadactyla) different site-types. The underlying numerical data are provided in S1 Data. (TIF) [file pbio.3003762.s015.tif]

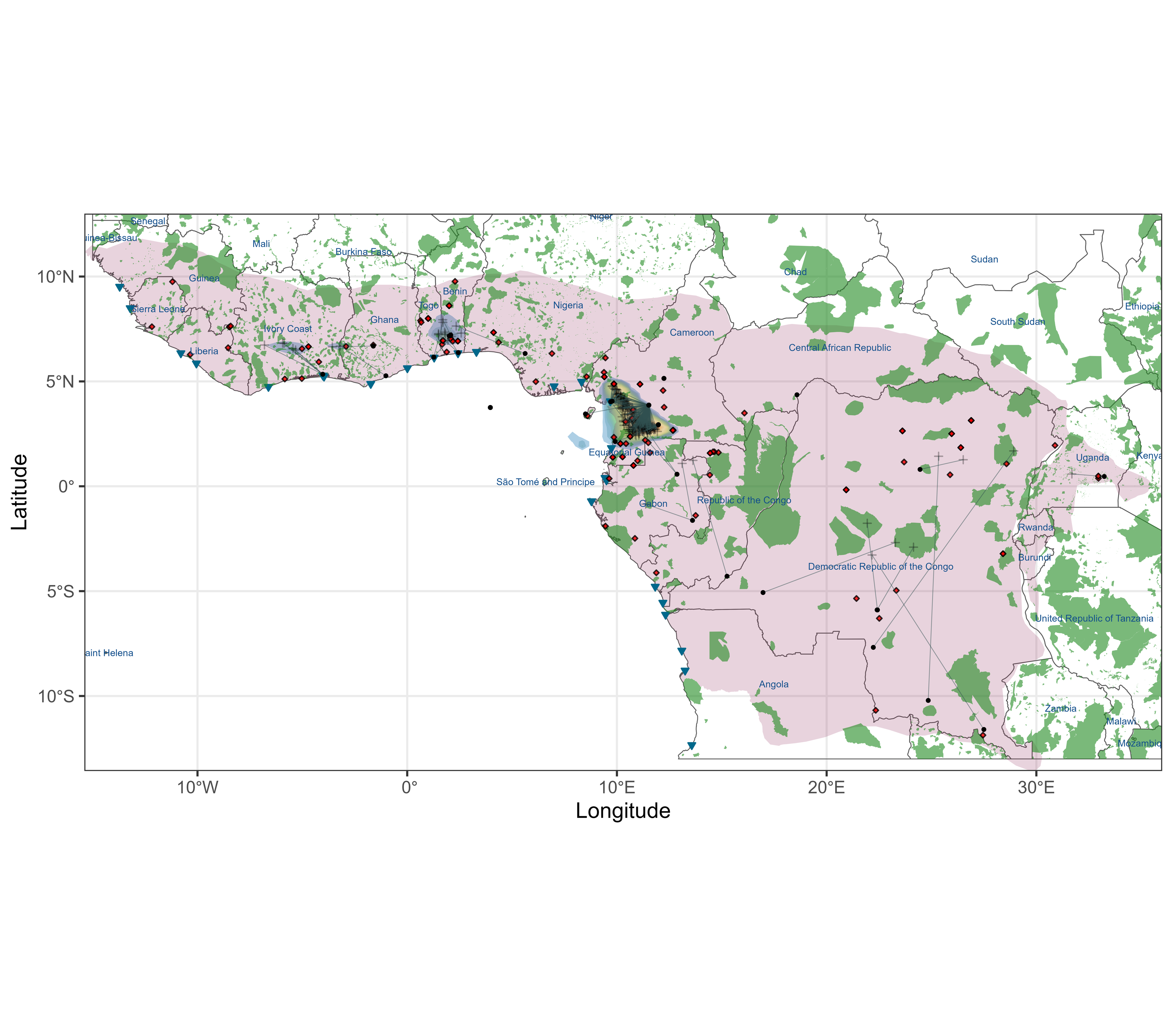

Supplement: S16 Fig — Training locations (red diamonds); seized samples (black circles) linked to their inferred origins (gray crosses) via gray (domestic) lines. Protected areas (green), roads (orange), and species range (purple shading) are shown for context. The heatmap highlights likely sourcing density, with red indicating hotspots and heatmap bounds reflecting bootstrap confidence. Base layers include country and port (blue triangles) data from Natural Earth (http://www.naturalearthdata.com), protected areas (green) from the World Database on Protected Areas (https://www.protectedplanet.net), and the modeled species’ range from occurrence data previously collated (https://data.nhm.ac.uk/dataset/natalie-cooper; [43]). The underlying numerical data are provided in S1 Data. (TIF) [file pbio.3003762.s016.tif]

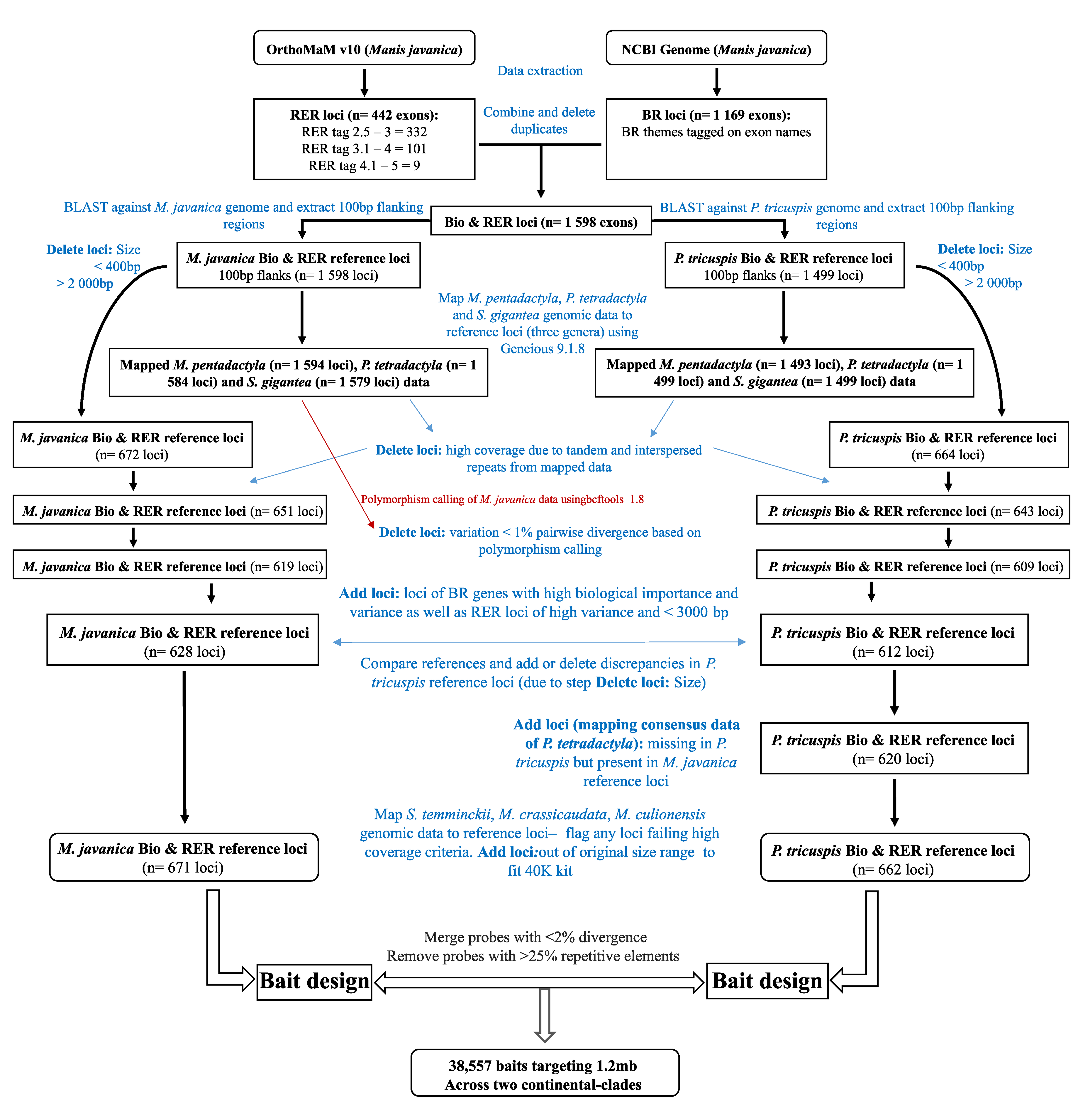

Supplement: S17 Fig — Based on the Manis javanica (GCF_001685135.1) and Phataginus tricuspis (GCA_004765945.1) reference assemblies, as well as OrthoMaM v10 database of orthologous genes. (TIF) [file pbio.3003762.s017.tif]
