# Supplementary material for: Targeted sequencing enhances detection of pangolin trafficking hotspots and dynamics of both domestic and global trade markets
Source: PLoS Biol. 2026 May 7;24(5):e3003762. doi: 10.1371/journal.pbio.3003762 (PMC13152146; doi:10.1371/journal.pbio.3003762)
Supplement: S1 Table — Model validation performance statistics are the model outputs from Locator, which provides the correlation between predicted and true locations for the validation samples used in model training (this is a measure of model fit). The lower the mean error (close to 0), the closer the validated sample predictions are from the training samples, and the higher the R2 values (x = longitude and y = latitude), the less the prediction will collapse towards the mean of each axis. Prediction variability is the distance between the centroid of all 100 bootstrap predictions and each bootstrapped predicted location when using all reference samples (the smaller the distance, the less variability in predictions, and thus the higher the confidence). Predictive error (or hold-out analysis) is defined as the geographic distance between a sample’s true sampling location and its predicted location when that reference sample is excluded from model training and treated as an unknown during prediction (smaller distances indicate higher spatial precision when reference data are withheld). We conducted two complementary hold-out approaches to measure predictive error: (i) a one-third hold-out, in which approximately one-third of reference samples distributed across the species’ range were removed in a single run and predicted, and (ii) Leave-One-Out Cross-Validation (LOOCV), in which each reference sample was iteratively removed per run, the model retrained, and the prediction error recorded for each sample until all reference samples have been removed at least once. Ref hold-out refers to the number of reference samples held out when conducting the one-third hold-out analyses of predictive error. (DOCX) [file pbio.3003762.s018.docx]

**S1 Table. Accuracy of origin tracing per species.**

|  | | **Model validation performance statistics** | | **Prediction variability (km)** | | | | **Predictive error – one-third hold out (km)** | | | | | **Predictive error – LOOCV hold out (km)** | | | |
| --- | --- | --- | --- | --- | --- | --- | --- | --- | --- | --- | --- | --- | --- | --- | --- | --- |
| **Species** | **Input data** | **Mean error** | **R^2^**  **(x-y)** | **Mean** | **Median** | **95% CI** | **Min-Max** | **Ref**  **hold-out** | **Mean** | **Median** | **95% CI** | **Min-Max** | **Mean** | **Median** | **95% CI** | **Min-Max** |
| White-bellied pangolin (*Phataginus tricuspis*) | Samples  =475  Reference  =171  Genotypes  =16 510 | 1.95 | x=0.95  y=0.71 | 60.19 | 45.92 | 8.72-154.78 | 0.31-1538.55 | 58 | 132.35 | 107.4 | 39.38-293.99 | 19.69-424.09 | 180.1 | 124.49 | 20.45-436.56 | 8.67-1660.35 |
| Sunda pangolin (*Manis javanica*) | Samples  =151 Reference=22  Genotypes=8 567 | 1.15 | x=0.99  y=1.0 | 294.94 | 197.97 | 37.55-948.61 | 0.57-2407.43 | 9 | 415.17 | 538.18 | 45.50-885.09 | 43.03-974.97 | 575.52 | 425.58 | 43.40-1527.27 | 16.51-1893.17 |
| Chinese pangolin (*Manis pentadactyla*) | Samples  =43  Reference=16  Genotypes=3 030 | 1.57 | x=1.0  y=1.0 | 411.03 | 223.67 | 34.14-1366.97 | 0.42-3014.88 | 6 | 1032.03 | 700.65 | 280.71-2126.19 | 230.58-2187.78 | 1055.72 | 892.23 | 82.16-2040.77 | 70.89-2141.50 |

Model validation performance statistics are the model outputs from Locator, which provides the correlation between predicted and true locations for the validation samples used in model training (this is a measure of model fit). The lower the mean error (close to 0), the closer the validated sample predictions are from the training samples, and the higher the R^2^ values (x=longitude and y=latitude), the less the prediction will collapse towards the mean of each axis. Prediction variability is the distance between the centroid of all 100 bootstrap predictions and each bootstrapped predicted location when using all reference samples (the smaller the distance, the less variability in predictions, and thus the higher the confidence). Predictive error (or hold-out analysis) is defined as the geographic distance between a sample’s true sampling location and its predicted location when that reference sample is excluded from model training and treated as an unknown during prediction (smaller distances indicate higher spatial precision when reference data are withheld). We conducted two complementary hold-out approaches to measure predictive error: (i) a one-third hold-out, in which approximately one-third of reference samples distributed across the species’ range were removed in a single run and predicted, and (ii) Leave-One-Out Cross-Validation (LOOCV), in which each reference sample was iteratively removed per run, the model retrained, and the prediction error recorded for each sample until all reference samples have been removed at least once. Ref hold-out refers to the number of reference samples held out when conducting the one-third hold-out analyses of predictive error.
