# Supplementary material for: Targeted sequencing enhances detection of pangolin trafficking hotspots and dynamics of both domestic and global trade markets
Source: PLoS Biol. 2026 May 7;24(5):e3003762. doi: 10.1371/journal.pbio.3003762 (PMC13152146; doi:10.1371/journal.pbio.3003762)
Supplement: S2 Table — Distances are calculated between the centroidal location of 100 bootstraped predictions and the sampling location. Sampling locations are separated into site types: long-range markets (regional to cross-country commercial trade linked by major routes and within major cities), seizures (stated as seizures by authorities or those collected outside home-range countries whereby they formed part of an international trade), unknown localities (did not have accurate enough sampling localities to be given a field-site type, usually museum specimens). Seizure estimates are not accurate for the white-bellied pangolin (Phataginus tricuspis) since they were either in Europe or China. (DOCX) [file pbio.3003762.s019.docx]

**S2 Table. Trade distances in kilometres (km) for each species and site type.**

| **Species** | **Site type** | **Number of samples** | **Mean** | **Median** | **95% CI - lower** | **95% CI - upper** | **Minimum** | **Maximum** |
| --- | --- | --- | --- | --- | --- | --- | --- | --- |
| White-bellied pangolin (*Phataginus tricuspis*) | Long-range markets | 275 | 136 | 117 | 49.7 | 265 | 22.9 | 1297 |
|  | Seizures | 24 | 696 | 785 | 257 | 847 | 251 | 852 |
|  | Unknown locality | 5 | 669 | 529 | 151 | 1598 | 123 | 1854 |
| Sunda pangolin (*Manis javanica*) | Long-range markets | 4 | 478 | 456 | 54.8 | 932 | 0.659 | 999 |
|  | Seizures | 121 | 769 | 669 | 130 | 2101 | 49.8 | 2545 |
|  | Unknown locality | 4 | 1738 | 1496 | 1310 | 2507 | 1292 | 2670 |
| Chinese pangolin (*Manis pentadactyla*) | Long-range markets | 6 | 749 | 702 | 464 | 1091 | 398 | 1165 |
|  | Seizures | 20 | 1101 | 1152 | 896 | 1255 | 829 | 1287 |
|  | Unknown locality | 1 | 492 | 492 | 492 | 492 | 492 | 492 |

Distances are calculated between the centroidal location of 100 bootstraped predictions and the sampling location. Sampling locations are separated into site types: long-range markets (regional to cross-country commercial trade linked by major routes and within major cities), seizures (stated as seizures by authorities or those collected outside home-range countries whereby they formed part of an international trade), unknown localities (did not have accurate enough sampling localities to be given a field-site type, usually museum specimens). Seizure estimates are not accurate for the white-bellied pangolin (*P. tricuspis*) since they were either in Europe or China.
