# Supplementary material for: Targeted sequencing enhances detection of pangolin trafficking hotspots and dynamics of both domestic and global trade markets
Source: PLoS Biol. 2026 May 7;24(5):e3003762. doi: 10.1371/journal.pbio.3003762 (PMC13152146; doi:10.1371/journal.pbio.3003762)
Supplement: S1 Text — (DOCX) [file pbio.3003762.s021.docx]

**Supplementary Text**

This contains additional details related to the materials and methods sections concerning the bait design workflow, sample metadata collection, capture efficiency estimates, and dealing with mislabelled or duplicated samples.

*Bait design for gene-capture across African and Asian pangolins in detail*

The bioinformatic workflow below was designed and implemented to produce a short-list of the most informative loci accounting for relative evolutionary rates (RER) and biological relevance (BR; genes that may have geographically linked signatures) using Geneious 9.1.8 ([https://www.geneious.com](http://www.geneious.com/); S10 Fig).

Loci forming the RER data were extracted from *M. javanica* in the OrthoMaM v10 database (<http://www.orthomam.univ-montp2.fr/orthomam_v10/>) of one-to-one orthologous genes encompassing 116 mammalian taxa [1], and were separated into three different categories tagged as: relative evolutionary rates of 2.5–3, 3.1–4 and 4.1–5 (maximum for v10 database). These RER estimates are based on a Super Distance Matrix procedure on highest-likelihood trees in the OrthoMaM pipeline, whereby higher figures relate to faster evolutionary rates [2]. Genes of biological relevance (BR; n = 112) were selected through a literature review of other animal models that exhibited geographic or physiological signatures that could be associated with pangolin-specific phenotypic or geoclimatic variation amongst species and populations. Our intent was not to identify direct phenotypic causation, but rather to include functionally informed loci with plausible links to environmental or geographic gradients that may generate spatially structured genetic variation. These themes were tagged on gene names and included climatic variability (temperature, moisture and altitude), external appearance [mainly scale, skin and hair colour but also keratin genes related to scale formation; see Choo *et al.* [3]], external reception (vision and taste), size and tail length, digestive pathway (chitinases and stomach acids), immunity, skull, and rostral morphology [based on variation found in pangolins by Ferreira-Cardoso *et al.* [4]], digging, circadian rhythm, behaviour, and pseudogenes.

These loci were blasted against the Sunda pangolin (*M. javanica*) RefSeq genome assembly [145x coverage - GCF_001685135.1; 3] and the white-bellied pangolin (*P. tricuspis*) genome assembly (30x coverage - GCA_004765945.1; unpublished) using Geneious 9.1.8 to extract 100bp flanking regions on either side of each locus (intronic and intergenic regions). It also accounted for missing data found in the OrthoMaM database due to the internal filtering step using trimAl [5] to remove false sequences and/or false codons, post-alignment [1], as these data were extracted from the exonic database. Initial filtering of loci was based on a size selection criterion whereby loci outside the 400bp – 2500bp range were removed. Genomic data from the Chinese [M. pentadactyla; GCA_000738955.1; 3], black-bellied [P. tricuspis; 6] *,* and giant [S. gigantea; 6] pangolins were mapped to the reference loci using Geneious 9.1.8 with default parameters. Polymorphisms were called using *bcftools mpileup* (parameter *-d/--max-depth* increased to 100 million) piped into *bcftools call* (*-c/--consensus-caller*) commands in BCFtools v1.8 [7]. This was only done for mapped data on the *M. javanica* reference loci owing to a high coverage of the reference genome and thus more accurate polymorphism estimation. Pairwise divergence values were calculated based on the number of polymorphisms per base pair length of loci, thus accounting for the relationship between locus size and number of polymorphisms. In the second filtering step, loci were removed based on too high coverage (>2x average coverage due to tandem or interspersed repeats) as well as pairwise divergence of lower than 1% between *M. javanica* reference loci and the three genera mapped to them (likely uninformative). Some loci were re-added if they were of potentially high geographical importance (two or more studies observing relevance), showed above the average variance, and were under 3000 bp.

Due to slight discrepancies in locus size between *P. tricuspis* and *M. javanica* reference loci, owing mainly to the low coverage of the *P. tricuspis* reference genome, these references were compared in order to rectify any missing loci deleted or added in *P. tricuspis* reference loci (these loci were double-checked against the coverage and variant criteria). One locus from the original *P. tricuspis* reference loci dataset was added, and another was taken from the consensus of the mapped *P. tetradactyla* genomic data to *M. javanica* reference loci, whilst the rest were flagged, but kept in the final *M. javanica* reference loci list (n=8). Lastly, the genomic data of *S. temminckii*, *M. culionensis,* and *M. crassicaudata* were mapped against the *M. javanica* and *P. tricuspis* reference loci for any additional loci to be flagged based on upper coverage (>2x average coverage due to tandem or interspersed repeats).

To reach the required 1.2mb of a 40K myBaits v5 (Daicel Arbor Biosciences) custom kit, we added 49 loci that fitted the filtering requirements but were outside the initial sizing requirements (between 350-400bp or between 2000-5000bp). This list was sent to myBaits® for final probe design, whereby baits for loci, or parts thereof, that were within a 2% divergence between the African (*P. tricuspis*) and Asian (*M. javanica*) continental clades were merged into a single bait. Baits that matched a part of the genome that had >25% repetitive elements were removed.

*Sample collection and metadata in detail*

Sample localities were cross-checked by first reading through the geographic notes, locality names, or coordinates provided by collectors or found in databases, and then finding these locations in Google Maps. For museum specimens, samples with clear geographic locality descriptions whereby GPS co-ordinates could easily be attributed to the description were used as references for trade tracing (i.e. 1.5 miles east from a town), while those with insufficient information (i.e. southern region of a country or a province) were placed in unknown locality categories. Once sampling localities were identified, the following metadata were added: additional/new names of locality, decimal coordinates (unless already provided), museum/fresh sample, and site-type (field site, restricted-range market, long-range market, seizure, unknown). Site-type was determined either by the authors’ knowledge of the sample location or by using information surrounding the locality with Google Maps (roads, infrastructure, and terrain). If the latter was used, this is how the site-types were determined:

- Field site – an isolated locality not near a town, city, or major route. These are supposed to be linked to pangolins found in the field, but could sometimes be small villages where the sample most likely came from a walkable distance in the surrounding field.
- Restricted-range market – a site that forms part of a small town not near major crossroads or routes. These samples likely came from the surrounding field or villages nearby, but not from regional or cross-country areas.
- Long-range market – city market, large city, crossroads, or town linked by major routes. These samples are likely part of a larger regional to cross-country commercial trade linked by major routes and within major cities, whereby restricted-range markets are likely feeding these markets. They were also collected by the collaborative network or museum collectors and do not constitute a seizure.
- Seizure – samples either stated as seizures by enforcement authorities or those collected outside home-range countries, whereby they formed part of an international trade. Points of seizure include entry points such as airports, seaports, and border control points, or places along the smuggling routes.
- Unknown – samples that did not have accurate enough sampling localities to be given a field-site type (information given by collectors at a continental–regional level - i.e. Africa/West Africa).

For collected samples, these were distributed as follows: trade seizures (n = 106 samples), long-range markets (n = 348), restricted-range markets (n = 161), field sites (n = 74), unknown sample locations (n = 22) (S1 Appendix). Genomic data collected from previously published articles [3, 8-10] were also assigned sample types according to the details provided in those articles or by their corresponding authors. This resulted in the following: trade seizures (n = 61 samples), large-range markets (n = 3), restricted-range markets (n =1), and field sites (n = 8) (S1 Appendix).

*Seizures in detail*

Trade seizure samples were derived from both samples collected through the collaborative network and from previously published genomic data (see S1 Appendix for details of each sample).

Seizure samples collected through the collaborative network comprise 106 samples. Five *P. tricuspis* samples from seizures by authorities (Institut de Recherche Criminelle de la Gendarmerie Nationale) at Aéroport Paris-Charles de Gaulle (Roissy CDG), originating from Cameroon between 2011 and 2012, and one originating from Bangui, Central African Republic. Nine *P. tricuspis* samples from a seizure by authorities at Brussels Airport, Belgium, originating from flights from Cameroon [11]. Eleven *M. pentadactyla* samples from the Taiwanese authorities (Council of Agriculture and the National Museum of Natural Science, Taiwan), more details can be found in Hsieh et al. Hsieh, Lee (12). Eighty-one *M. javanica* samples were seized from throughout Indonesian ports of trade (Bogor, Jember, Medan, Nyaru menteng, Pangkalanbun, Sukabumi, Surabaya, Tanggamus lampung, Tegal alur Jakarta), collected by the Indonesian Institute of Science between 2008 and 2016, where more details can be found in Nash, Wirdateti (13).

Seizure sample genomic data representing 80 samples stem from previously published research by Hu, Hao (8), Wang, Lan (9) and Gu, Wu (10); more details are provided in both articles and S1 Appendix. Hu, Hao (8) collected *M. javanica* (n = 52) and *M. pentadactyla* (n = 3) samples from seizures conducted by authorities across Yunnan (including a Sino-Burmese border post) and Guangdong provinces, China, between 2003 and 2017. These were held by the Animal Branch of the Germplasm Bank of Wild Species, Chinese Academy of Sciences and the Guangzhou Wildlife Rescue Center). Wang, Lan (9) collected *M. pentadactyla* (n = 6) samples from seizures conducted by forestry police across Guangdong province, China, which were held at the Guangzhou Wildlife Rescue Center. Gu, Wu (10) collected *P. tricuspis* (n = 19) samples from seizures conducted by the Yunnan Provincial Forest Public Security Bureau across Yunnan Province, China, which were held at the Animal Branch of the Germplasm Bank of Wild Species of Chinese Academy of Sciences. These publications provided no information on potential sources or on how the seizures were conducted.

*Simple capture efficiency estimates to guide sequencing efforts*

To guide further laboratory processing and sequencing efforts, we employed a simple pipeline to clean newly sequenced raw reads (fastq), map them to the *M. javanica* baits reference (BWA), and undergo post-mapping cleaning with summary statistics (SAMtools, Qualimap, and custom scripts). These summary statistics included capture efficiency, average per-site depth, average percentage of the target regions covered with at least 10x depth, and mean Phred mapping quality. Capture efficiency was calculated by the percentage of reads mapped (post-mapping quality cleaning) compared to those that were sequenced (post-sequence quality cleaning). We used this to test pooling limits, whereby both capture efficiency and depth of coverage were not affected by pooling of up to 12 samples per capture reaction (S1C and S1D Fig)

*Mislabeled species and duplicate sample cleanup in detail*

We conducted a pre-phylogeny and principal components analysis (PCA) for the three species with representatives from all eight recognised and the ninth unrecognised species [6, 10]. These were split into two continental clades (African species for *P. tricuspis* and Asian species for *M. javanica* and *M. pentadactyla*). We used BCFtools v1.8 to obtain genotype likelihoods per continental clade and filtered them by depth (8x-350x), site quality (>20 Phred), site missingness (<30%), and variant type (removed indels) with VCFtools v0.1.15 [14]. VCF files were converted to Fasta alignments per continental clade with *vcf2phylip.py* [15], before undergoing Neighbour-Joining tree construction in MEGA X [16] using the Kimura 2-parameter model of evolution. The PCA was conducted on each continental clade using PLINK v2.0 [17], in which individuals above 95% missing data were removed. To remove potential sampling duplicates (especially for seizures), we used a kinship analysis. Pairwise relatedness estimates based on the original VCF call files were determined using the KING algorithm [18], which was run with *relatedness2* in VCFtools v0.1.15. An individual from each pairwise relatedness comparison above 0.354 (duplicate / monozygotic twin) was removed from further analyses, whilst mislabeled individuals were correctly re-assigned to their species based on the continent-specific phylogenies and PCAs.

*Species’ range estimates for figures*

Species range polygons were reconstructed using georeferenced occurrence records that were collated for each pangolin species by Buckingham, Curry (19) (<https://data.nhm.ac.uk/dataset/natalie-cooper>). For each species, occurrence datasets were filtered to remove obvious geographic outliers that were inconsistent with known species distributions [20]. Range polygons were generated using concave hull (alpha hull) modelling implemented in the *concaveman* R package [21] using a concavity threshold of 2 and applying a 50km buffer to smooth polygon boundaries. These were intersected with the terrestrial landmass base layer from Natural Earth (https://www.naturalearthdata.com/) to exclude marine range predictions and were refined using detailed distribution information for each species in Challender, Nash (20). These polygons represent approximate visualisations of species distributions generated for analytical and illustrative purposes and should not be interpreted as authoritative species range boundaries. We suggest following the IUCN Red List of Threatened Species for the most comprehensive expert-derived range assessments.

# S1 Text References

1. Scornavacca C, Belkhir K, Lopez J, Dernat R, Delsuc F, Douzery EJP, et al. OrthoMaM v10: Scaling-up orthologous coding sequence and exon alignments with more than one hundred mammalian genomes. Molecular Biology and Evolution. 2019;36(4):861-2. doi: 10.1093/molbev/msz015.

2. Ranwez V, Delsuc F, Ranwez S, Belkhir K, Tilak M-K, Douzery EJP. OrthoMaM: A database of orthologous genomic markers for placental mammal phylogenetics. BMC Evolutionary Biology. 2007;7(1):241. doi: 10.1186/1471-2148-7-241.

3. Choo SW, Rayko M, Tan TK, Hari R, Komissarov A, Wee WY, et al. Pangolin genomes and the evolution of mammalian scales and immunity. Genome Research. 2016;26(10):1312-22. doi: 10.1101/gr.203521.115.

4. Ferreira-Cardoso S, Billet G, Gaubert P, Delsuc F, Hautier L. Skull shape variation in extant pangolins (Pholidota: Manidae): allometric patterns and systematic implications. Zoological Journal of the Linnean Society. 2019;188(1):255-75. doi: 10.1093/zoolinnean/zlz096.

5. Capella-Gutiérrez S, Silla-Martínez JM, Gabaldón T. trimAl: a tool for automated alignment trimming in large-scale phylogenetic analyses. Bioinformatics. 2009;25(15):1972-3. doi: 10.1093/bioinformatics/btp348.

6. Heighton SP, Allio R, Murienne J, Salmona J, Meng H, Scornavacca C, et al. Pangolin Genomes Offer Key Insights and Resources for the World’s Most Trafficked Wild Mammals. Molecular Biology and Evolution. 2023;40(10). doi: 10.1093/molbev/msad190.

7. Danecek P, Bonfield JK, Liddle J, Marshall J, Ohan V, Pollard MO, et al. Twelve years of SAMtools and BCFtools. Gigascience. 2021;10(2). Epub 2021/02/17. doi: 10.1093/gigascience/giab008. PubMed PMID: 33590861; PubMed Central PMCID: PMCPMC7931819.

8. Hu J-Y, Hao Z-Q, Frantz L, Wu S-F, Chen W, Jiang Y-F, et al. Genomic consequences of population decline in critically endangered pangolins and their demographic histories. National Science Review. 2020;7(4):798-814. doi: 10.1093/nsr/nwaa031.

9. Wang Q, Lan T, Li H, Sahu SK, Shi M, Zhu Y, et al. Whole-genome resequencing of Chinese pangolins reveals a population structure and provides insights into their conservation. Communications Biology. 2022;5(1):821. doi: 10.1038/s42003-022-03757-3.

10. Gu T-T, Wu H, Yang F, Gaubert P, Heighton SP, Fu Y, et al. Genomic analysis reveals a cryptic pangolin species. Proceedings of the National Academy of Sciences. 2023;120(40):e2304096120. doi: doi:10.1073/pnas.2304096120.

11. Din Dipita A, Missoup AD, Aguillon S, Lecompte E, Momboua BR, Chaber A-L, et al. Genetic tracing of the illegal trade of the white-bellied pangolin (Phataginus tricuspis) in western Central Africa. Scientific Reports. 2024;14(1):13131. doi: 10.1038/s41598-024-63666-9.

12. Hsieh H-M, Lee JC-I, Wu J-H, Chen C-A, Chen Y-J, Wang G-B, et al. Establishing the pangolin mitochondrial D-loop sequences from the confiscated scales. Forensic Science International: Genetics. 2011;5(4):303-7. doi: <https://doi.org/10.1016/j.fsigen.2010.06.003>.

13. Nash HC, Wirdateti, Low GW, Choo SW, Chong JL, Semiadi G, et al. Conservation genomics reveals possible illegal trade routes and admixture across pangolin lineages in Southeast Asia. Conservation Genetics. 2018;19(5):1083-95. doi: 10.1007/s10592-018-1080-9.

14. Danecek P, Auton A, Abecasis G, Albers CA, Banks E, DePristo MA, et al. The variant call format and VCFtools. Bioinformatics. 2011;27(15):2156-8. doi: 10.1093/bioinformatics/btr330.

15. Ortiz E. vcf2phylip v2. 0: convert a VCF matrix into several matrix formats for phylogenetic analysis. URL <https://doi> org/105281/zenodo. 2019;2540861.

16. Kumar S, Stecher G, Li M, Knyaz C, Tamura K. MEGA X: Molecular Evolutionary Genetics Analysis across Computing Platforms. Molecular Biology and Evolution. 2018;35(6):1547-9. doi: 10.1093/molbev/msy096.

17. Chang CC, Chow CC, Tellier LC, Vattikuti S, Purcell SM, Lee JJ. Second-generation PLINK: rising to the challenge of larger and richer datasets. GigaScience. 2015;4(1). doi: 10.1186/s13742-015-0047-8.

18. Manichaikul A, Mychaleckyj JC, Rich SS, Daly K, Sale M, Chen W-M. Robust relationship inference in genome-wide association studies. Bioinformatics. 2010;26(22):2867-73. doi: 10.1093/bioinformatics/btq559.

19. Buckingham E, Curry J, Emogor C, Tomsett L, Cooper N. Using natural history collections to investigate changes in pangolin (Pholidota: Manidae) geographic ranges through time. PeerJ. 2021;9:e10843. doi: 10.7717/peerj.10843.

20. Challender DW, Nash HC, Waterman C. Pangolins: Science, Society and Conservation. London, UK: Academic Press; 2020.

21. Park J-S, Oh S-J. A New Concave Hull Algorithm and Concaveness Measure for n-dimensional Datasets. J inf sci eng. 2012;28(3):587-600.
